# The *Mexico SimSmoke* tobacco control policy model: Development of a simulation model of daily and nondaily cigarette smoking

**DOI:** 10.1371/journal.pone.0248215

**Published:** 2021-06-21

**Authors:** Luz María Sánchez-Romero, Luis Zavala-Arciniega, Luz Myriam Reynales-Shigematsu, Belén Sáenz de Miera-Juárez, Zhe Yuan, Yameng Li, Yan Kwan Lau, Nancy L. Fleischer, Rafael Meza, James F. Thrasher, David T. Levy

**Affiliations:** 1 Lombardi Comprehensive Cancer Center, Georgetown University, Washington, DC, United States of America; 2 Tobacco Research Department, National Institute of Public Health, Cuernavaca, México; 3 Department of Epidemiology, University of Michigan School Public Health, Ann Arbor, Michigan, United States of America; 4 Department of Economics, Autonomous University of Baja California Sur, La Paz, México; 5 Department of Health Promotion, Education & Behavior, Arnold School of Public Health, University of South Carolina, Columbia, South Carolina, United States of America; Universidade Federal de Sao Paulo, BRAZIL

## Abstract

**Background:**

Nondaily smoking has been on the rise, especially in Mexico. While Mexico has strengthened its tobacco control policies, their effects on nondaily smokers have gone largely unexamined. We developed a simulation model to estimate the impact of tobacco control policies on daily and nondaily smoking in Mexico.

**Methods:**

A previously validated *Mexico SimSmoke* model that estimated overall trends in smoking prevalence from 2002 through 2013 was extended to 2018 and adapted to distinguish daily and nondaily smoking prevalence. The model was then validated using data from Mexican surveys through 2016. To gauge the potential effects of policies, we compared the trends in smoking under current policies with trends from policies kept at their 2002 levels.

**Results:**

Between 2002 and 2016, *Mexico SimSmoke* underestimated the reduction in male and female daily smoking rates. For nondaily smoking, *SimSmoke* predicted a decline among both males and females, while survey rates showed increasing rates in both genders, primarily among ages 15–44. Of the total reduction in smoking rates predicted by the model by 2018, tax policies account for more than 55%, followed by health warnings, cessation treatment, smoke-free air laws, and tobacco control spending.

**Conclusions:**

Although *Mexico SimSmoke* did not successfully explain trends in daily and nondaily smoking, it helps to identify gaps in surveillance and policy evaluation for nondaily smokers. Future research should consider appropriate measures of nondaily smoking prevalence, trajectories between daily and nondaily smoking, and the separate impact of tobacco control policies on each group.

## Introduction

In 2004, Mexico ratified the World Health Organization Framework Convention on Tobacco Control (FCTC) treaty. Since then, Mexico has adopted stronger tobacco advertising restrictions, package health warnings and increased cigarette taxes [1]. Mexico’s prevalence of overall smoking has declined from 21.5% in 2007 to 19.0% in 2016. In particular, daily smoking prevalence decreased by 50% from 13.5% in 2002 to 7.0% in 2016. Meanwhile nondaily smoking prevalence increased by 35% from 8.8% in 2009 to 11.9% in 2016, comprising a large proportion of smokers in Mexico [2, 3]. This pattern of growth in the proportion of non-daily smokers is not only occurring in Mexico, but also in many middle-income countries, such as Brazil, India, Indonesia and Thailand [4], as well as in many high-income countries [5, 6].

Studies have suggested that the increase in nondaily smoking in Mexico is related to the increase in popularity of new tobacco products, particularly flavored capsule cigarettes [7], the increase in the sale of single cigarettes and multiproduct use in the population [7, 8], and the absence of cessation strategies targeted to nondaily smokers [9]. In addition, Mexico has implemented tobacco policies focused on daily cigarette smokers, which rarely consider nondaily and multiproduct use patterns [1, 10]. As nondaily smoking continues to grow and patterns of use continue to change (e.g., towards social users, infrequent and frequent nondaily users, and multiproduct users), it is important to evaluate the differential effect of current tobacco control policies on nondaily vs daily smokers.

To evaluate the potential effect of Mexico’s policies, the *Mexico SimSmoke* tobacco control simulation model was developed and validated through 2011. The model showed that overall smoking prevalence was reduced by 30% potentially due to policies implemented between 2002 and 2013 [11]. However, neither that model nor *SimSmoke* models developed for other countries have distinguished nondaily from daily smokers.

This study describes the update of the *Mexico SimSmoke*, to separately consider daily and nondaily smokers. This version builds upon the previously validated model [11, 12], extends policy level data to 2018 and estimates the potential role of implemented policies on daily and nondaily smoking prevalence.

## Methods

*Mexico SimSmoke* is built upon separate population, smoking prevalence, smoking-attributable deaths, and policy modules. The model begins in 2002 to take advantage of the large-scale National Addictions Survey (ENA). ENA 2002 precedes the implementation of most major tobacco policies in Mexico. The model assumes a discrete-time, first-order Markov process to project future age and gender-specific population growth, smoking rates, and deaths from 2002 through 2060. Briefly, total population growth evolves through births, deaths, and migration. Smoking prevalence evolves through smoking initiation, cessation, and relapse, and changes in tobacco control policies.

### Population

Population, mortality, and migration data for 2002–2050 by single age (0 to 85+) and gender are from the Mexico National Council of Population (CONAPO) [13]. The population from birth to age 14 is classified as never smokers since minimal smoking occurs before that age [11, 12], and then evolves through mortality and net migration. *Mexico SimSmoke* predicts the population size by age group through 2018 within 5% of the CONAPO estimates.

### Smoking prevalence

The baseline data for smoking prevalence are primarily from the 2002 National Addictions Survey (ENA) [14], a nationally representative multistage household survey of Mexicans aged 15 to 65. Current smokers are those who reported to have “*ever smoked tobacco in their lifetime*” and “*had smoked in the past-30 days*”. Current smokers were further classified as daily or nondaily smokers based on their answer to the question “*Approximately*, *how many cigarettes have you smoked daily in the past 30-days*?*”*. Nondaily users are those who reported that they *“do not smoke every day”*. Former smokers are classified by the number of years quit. In the previous *Mexico SimSmoke* [11, 12], we limited the modeling exercise to established smokers, restricted to those who reported that they had smoked at least 100 cigarettes in their lifetime [3, 15]. However, because a large percentage of both daily and especially nondaily smokers, including older adults, reported that they have not smoked 100 cigarettes in their lifetime [3], we do not apply the 100-cigarette criterion for the current model. Instead, we conduct a sensitivity analysis applying that criterion.

Because ENA 2002 only contained population data through age 65, smoking prevalence data for those above age 65 was obtained from the 2000 National Health Survey (ENSA). The ENA 2000 estimates for ages 65 and above are adjusted by the relative difference in estimates for the age 45–64 age group between ENA and ENSA.

Baseline initiation rates at each age, after age 14, are calculated as the difference between the 2002 baseline smoking rate at each age and the smoking rate at the previous age. Initiation occurs from ages 14 through 28, based on the ages at which we observed increases in daily and nondaily smoking prevalence.

The cessation rate is calculated as the number of former smokers who quit during the last year divided by total smokers the previous year. Although estimated cessation rates were slightly higher for nondaily than daily smokers, we apply the same cessation rates to both groups, since nondaily smoker cessation rates may include a higher percentage of short-term quitters that are more likely to relapse. Due to a lack of suitable Mexican relapse rates in 2002, we used US rates [16] for both daily and nondaily smokers differentiated by quit years.

### Tobacco control policies

Based on the World Health Organization MPOWER measures [17], *SimSmok*e evaluates the impact of cigarette excise taxes, smoke–free air laws, media campaigns, marketing bans, health warnings, cessation treatment policies, and youth access restriction. Although Mexico is an upper-middle-income country, we use the policy effects for high-income nations, because smoking trends indicate that Mexico is at later stages in the smoking epidemic.

Table 1 describes the policies and effect sizes. The effect sizes are applied as percentage reductions in smoking prevalence in the initial year and as reduced initiation and increased cessation rates in later years. Although effect sizes are based primarily on studies consisting mostly of daily smokers, the same policy effects are applied to daily and nondaily smokers. However, because nondaily smokers generally face less exposure to the impact of laws (e.g., due to less use), we conduct sensitivity analyses assuming half the policy effect sizes for nondaily smokers.

**Table 1:** Tobacco control policies, specifications and policy effect sizes*.

Policy	Description	Policy Effect Size**
**Cigarette Excise Taxes**		
**Cigarette price/tax**	The effect of taxes is directly incorporated through the average price after tax. The price elasticity is used to convert the % price changes into effect sizes.	Elasticities -0.4 for ages 14–17 -0.3 for ages 18–24 -0.2 for ages 25–34 -0.1 for ages 35–64 -0.2 for ages 65+
**Smoke-Free Air Laws**		
**Worksite smoking ban**	Ban in all indoor worksites, with strong enforcement of laws (reduced by 1/3 if allowed in ventilated areas and by 2/3 if allowed in common areas)	-6%
**Restaurant smoking ban**	Ban in all indoor restaurants (scaled for lower coverage), with strong enforcement of laws	-2%
**Pubs and bars smoking ban**	Ban in all indoor in pubs and bars (scaled for lower coverage), with strong enforcement of laws	-1%
**Other place bans**	Ban in 3 out of 4 government buildings (scaled for lower coverage), retail stores, public transportation, and elevators, with strong enforcement of laws	-1%
**Enforcement and Publicity**	Government agency enforces the laws and publicity via tobacco control campaigns	Effects reduced 50% absent publicity and enforcement
**Tobacco Control Campaigns**		
**High publicity media campaign**	Campaign publicized heavily on TV and at least some other media, with a social marketing approach	-6.5%
**Medium publicity media campaign**	Campaign publicized sporadically on TV and at least some other media	-3.25%
**Low publicity media campaign**	Campaign publicized only sporadically in newspaper, billboard, or some other media	-1.63%
**Marketing Restrictions**		
**Comprehensive marketing ban**	Ban is applied to television, radio, print, billboard, in-store displays, sponsorships and free samples (all indirect marketing)	-5% prevalence, -8% initiation, +4% cessation
**Moderate advertising ban**	Ban is applied to all media (television, radio, print, billboard) plus one indirect marketing medium	-3% prevalence, -4% initiation, +2% cessation
**Minimal advertising ban**	Ban is applied to some television, radio, print, and billboard	-1% prevalence and -1% initiation only
**Enforcement**	Government agency enforces the laws	Effects reduced 50% absent enforcement
**Health Warnings**		
**High**	Labels are large, bold and graphic, and cover at least 50% of pack	-4% prevalence, -6% initiation, +10% cessation
**Moderate**	Laws cover at least 30% of package, not bold or graphic	-2% prevalence, -2% initiation, +4% cessation
**Low**	Laws cover less than 30 of package, not bold or graphic	-1% prevalence, -1% initiation, +2% cessation
**Cessation Treatment Policies**		
**Availability of pharmacotherapies**	Legality of nicotine replacement therapy and/or Bupropion and Varenicline	-1% prevalence, +6% cessation
**Cessation treatment financial coverage**	Payments to cover pharmacotherapy and behavioral cessation treatment with high publicity (Effect size reduced by 12.5% with moderate publicity and 18.75% with low publicity)	-2.25% prevalence, +12% cessation
**Quit line**	Three quit line types: passive, proactive and active with follow-up. (Effect size reduced by 1/3 if quit line is proactive, reduced by 2/3 if quit line passive).	-0.75% prevalence, +7.5% cessation
**Brief interventions**	Advice by health care provider to quit and methods provided	-1% prevalence, +8% cessation
**All cessation policies combined**	Complete availability and reimbursement of pharmaco- and behavioral treatments, quit lines, and brief interventions	-5.15% prevalence, +42.7% cessation
**Youth Access Policies**		
**Strong enforcement & well publicized**	Compliance checks are conducted 4 times per year per outlet, penalties are potent and enforced with heavy publicity	-16% initiation and prevalence for ages 16–17 and -24% ages 14–15
**Moderate enforcement with some publicity**	Compliance checks are conducted regularly, penalties are potent, and publicity and merchant training are included	-8% initiation and prevalence for ages 16–17 and -12% ages 14–15
**Low enforcement**	Compliance checks are conducted sporadically, penalties are weak	-2% initiation and prevalence for ages 16–17 and -3% ages 14–15
**Vending machine restrictions**	Total ban, with strong enforcement	8%
**Self-service restrictions**	Total ban, with strong enforcement	4%

* References can be found in the text.

** Policy effect sizes are assumed to be the same in reducing prevalence rates (in the first year) and initiation rates (after the first year) and increasing cessation rates (after the first year) unless otherwise stated.

*Mexico SimSmoke* incorporates policy levels from 2002 to 2018, obtained from MPOWER Reports [17, 18], the Tobacco Control Report for the Region of the Americas 2013 and 2018 [19, 20], and communication with researchers from the Mexican National Institute of Public Health [1]. Mean annual real cigarette prices were obtained from the National Institute of Statistics and Geography (INEGI) [21].

Mexico had low-level smoke-free air laws for worksites except for health care and educational facilities from 2002 through 2007. In 2008, Mexico City and the state of Tabasco implemented laws for smoke-free workplaces, restaurants, and bars [12, 22]. Similar laws were adopted by other states since 2011. As a percent of the population, high-level worksite bans were 10% in 2008–2009, increasing to 18% in 2010–2011, and to 45% in 2014–2016. Restaurant and bar bans increased from 0% in 2002 to 60% for 2010–2012, and 70% from 2013 onwards. Based on the literature [17, 18, 23], the enforcement level is estimated at four on a ten-point scale for all years.

Before 2005, Mexico had limited tobacco control media campaigns. A national media campaign began in 2005, with a subsequent campaign in 2013 [10]. Mexico has a national tobacco control agency and in 2008 they reported a tobacco control expenditure of $0.00013 USD per capita for age 15+ [24], increasing to $0.08 USD in 2017 [25]. Tobacco control campaign levels increased from no campaign to a low-level campaign in 2005 [26] and remained at that level for all years.

Mexico had minimal restrictions on tobacco advertising in 2002. Advertising was banned for most hours on TV and radio in 2004 [26], and was expanded to all television and radio hours and billboards and some types of sponsorships in 2009 [27]. However, data from the 2009 Global Adult Tobacco Survey (GATS) and the National Survey of Drugs, Alcohol, and Tobacco Consumption (ENCODAT) indicated that more than 30% of the population still noticed retail advertisements [28, 29]. Advertising bans begin at a 50% low level in 2002–2003, increasing to 100% low-level in 2004 and to 75% moderate and 25% low-level in 2009, while enforcement is set at 5 out of 10 for all years [18, 29].

In 2002, health warnings covered a small portion of the pack (low-level). In 2004, they were increased to 50% of the package back (moderate-level) and then modified to 30% of the front with pictorial warnings and 100% of the back with only text warnings (high-level) in 2010 [30].

Cessation treatment policies consider the availability of treatment, including quit line and health care worker involvement. Nicotine replacement therapy was available without prescriptions from 2002 to 2018 [31]. From 2006 to 2012, a national program provided behavioral therapy, but with few referrals [12]. Cessation support has been partially covered in most primary care facilities, some hospitals, and communities since 2007 and has been available in some other places and offices since 2010. However, the use of pharmacotherapy as a cessation method decreased from 6.1% in 2009 to 3.5% in 2015 [32]. A quit line has been in place since 2008, but with minimal referrals [1], and is considered at a low level. Data from 2009 GATS [28] and 2016 ENCODAT [29] indicate that brief interventions were provided to about 25% of smokers with minimal advice to quit. We set the brief intervention index to a 20% level for all years.

Regarding youth access policies, Mexico has banned the sale of cigarettes to youth under age 18, but with minimal enforcement. According to the 2009 GATS, over 60% of youth were allowed cigarette purchase [33], and the 2016 ENCODAT [29] showed that 75% of adolescent smokers bought single cigarettes and 88% were not requested to show proof of age, which is considered low-level enforcement.

### Model validation and projection

The model is validated by comparing predicted gender-specific daily and nondaily smoking prevalence rates from *SimSmoke* to estimates from five Mexican national representative surveys: 2011 ENA, 2012 National Health and Nutrition Survey (ENSANUT) [34], 2009 and 2015 GATS, and 2016 ENCODAT. Since 2009, Mexican national health surveys estimate the prevalence of daily and nondaily smoking based on the standardized question “Do you currently smoke tobacco on a daily basis, less than daily, or not at all?” Nondaily users are defined as those who answer, “less than daily”. We focus our validation on the 2016 ENCODAT, the most recent survey with the same design as the 2002 ENA. We further validate the model by age group.

To examine the potential policies’ effects on daily and nondaily smoking prevalence, we compare *SimSmoke* prevalence rates with policies implemented between 2002 and 2018 (status quo) to policies set at their 2002 level (counterfactual). To gauge the effect of each policy, we incorporate changes in the selected policy while holding other policies at their 2002 level. The impact of the policies is measured as the relative difference in prevalence between the status quo and counterfactual. Since *SimSmoke* policies effects are not additive, the role of an individual policy is measured relative to the summed effects of all individual policies.

Further details of the development and projections of the model are provided in a S1 Report.

## Results

### Validation of current daily and nondaily smoking prevalence

**Fig 1:**
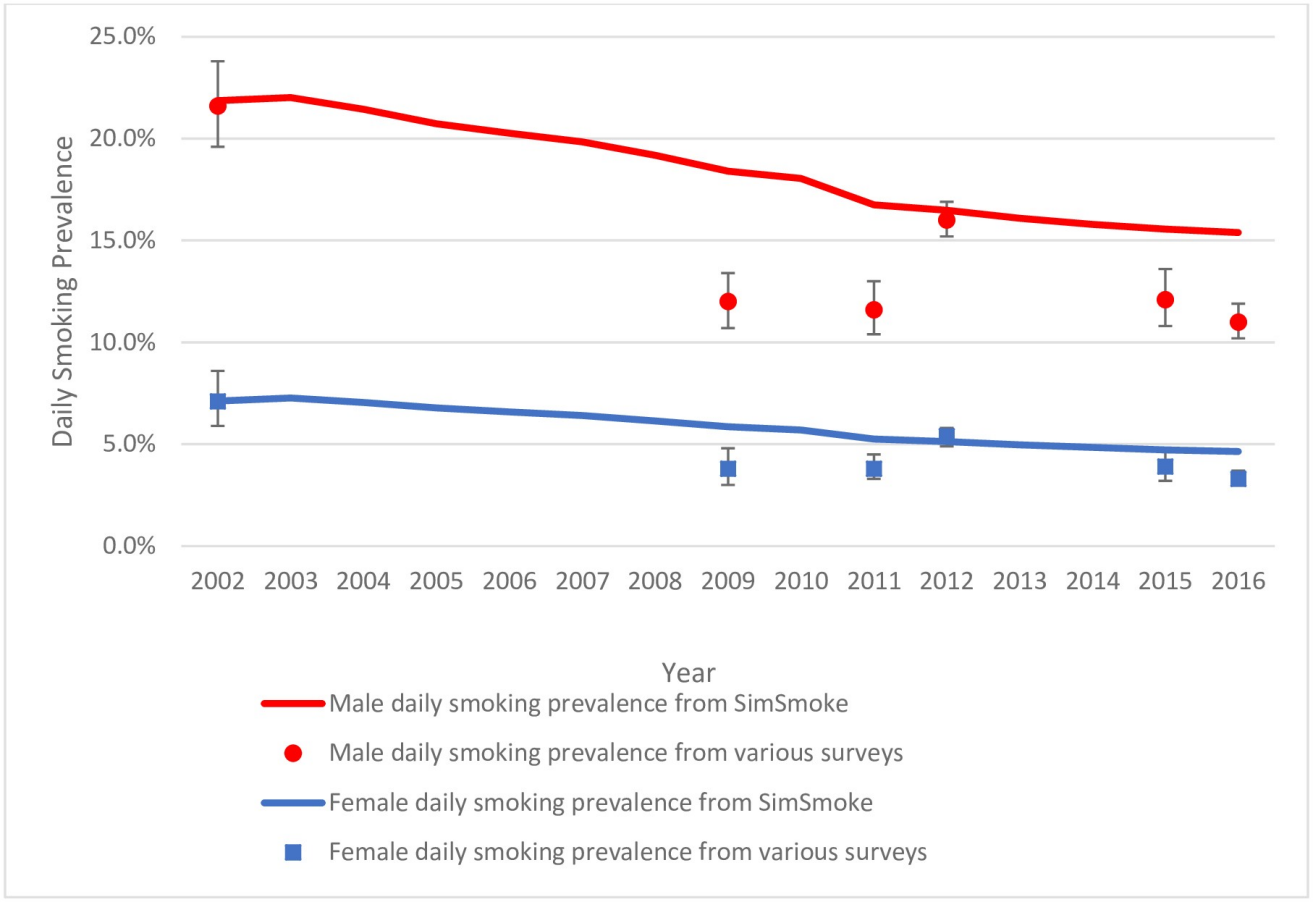
Validation of daily smoking prevalence, ages 15–65 by gender, from *Mexico SimSmoke* and various national surveys,* 2002–2016. *The survey point estimates (indicated by dots) and 95% confidence intervals were obtained from the National Addictions Survey (ENA) in 2002 and 2011, the Global Adult Tobacco Survey (GATS) in 2009 and 2015, the National Health and Nutrition Survey (ENSANUT) in 2012, and the National Survey of Drugs, Alcohol, and Tobacco Consumption (ENCODAT) in 2016.

**Fig 2:**
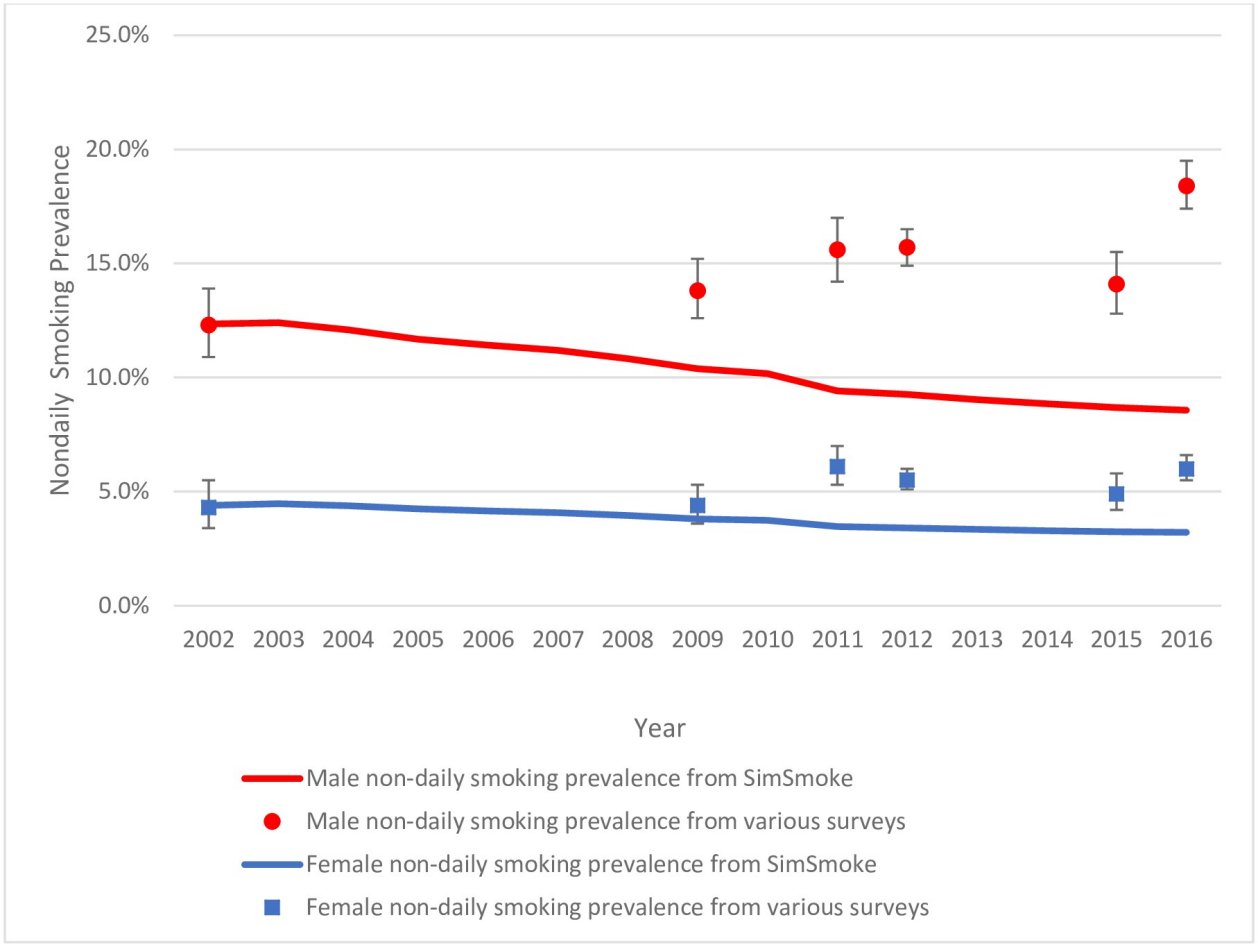
Validation of nondaily smoking prevalence, ages 15–65 by gender, from *Mexico SimSmoke* and various national surveys*, 2002–2016. *The survey point estimates (indicated by dots) and 95% confidence intervals were obtained from the National Addictions Survey (ENA) in 2002 and 2011, the Global Adult Tobacco Survey (GATS) in 2009 and 2015, the National Health and Nutrition Survey (ENSANUT) in 2012, and the National Survey of Drugs, Alcohol, and Tobacco Consumption (ENCODAT) in 2016.

Figs 1 and 2 present the daily and nondaily smoking prevalence projections by gender from *SimSmoke* and Mexico surveys using smoking estimates without the 100-cigarette lifetime criterion and assuming the same policy effects for nondaily and daily smokers.

For daily smoking prevalence ages 15–65, *SimSmoke* predicts a decline to 15.4% for males and 4.6% for females in 2016, compared to 11.0% and 3.3% respectively from ENCODAT. *SimSmoke* daily estimates for both genders, are above the survey’s upper limits of the 95% confidence interval (CI) but fall within the 95% CI for 2012. By age group (S2.1 Table in S1 Report), *SimSmoke* overestimates male daily smoking prevalence (especially for ages 25–44), except for ages 15–44 in 2012, and overestimates female daily smoking rates for all age groups (especially for ages 15–24) for all surveys except for ages 25–65 in 2012.

For male nondaily smoking prevalence ages 15–65 in 2016, *SimSmoke* predicts a decline for males to 8.6% compared to an increase to 18.4% from ENCODAT and a decline for females to 3.2% compared to an increase to 6.0% from ENCODAT. Nondaily *SimSmoke* predictions are below the 95% CIs of all survey estimates, except for females from the 2009 GATS. For ages 18–24 and 25–64, *SimSmoke* nondaily smoking predictions for both genders are below all survey 95% CIs, except for females ages 25–44 in 2009. The model predicts better for ages 45–65 but still underestimates nondaily prevalence for males in 2016.

### The potential effect of tobacco control policies implemented through 2018

Table 2 shows the results for daily and nondaily smoking prevalence comparing the status-quo scenario (with actual policies) to the counterfactual (no new policies implemented after 2002).

**Table 2:** Current smoking prevalence by smoking status and gender, from *Mexico SimSmoke* under different policy scenarios, 2002–2060.

**Males**				
**Scenarios**	**Type of smokers**	**2002**	**2018**	**Relative difference in 2018**
Counterfactual*	Non-Daily	11.9%	11.3%	-
	Daily	21.3%	19.6%	-
Status quo**	Non-Daily	11.9%	8.2%	-26.9%
	Daily	21.3%	14.6%	-25.6%
**Individual Policy**				
Cigarette Price	Non-Daily	11.9%	9.5%	-16.0%
	Daily	21.3%	16.8%	-14.4%
Smoke-free air laws	Non-Daily	11.9%	10.9%	-2.8%
	Daily	21.3%	19.1%	-2.7%
Media campaigns	Non-Daily	11.9%	11.1%	-1.2%
	Daily	21.3%	19.4%	-1.1%
Cessation treatment policy	Non-Daily	11.9%	11.0%	-2.0%
	Daily	21.3%	19.2%	-2.1%
Health warnings	Non-Daily	11.9%	10.8%	-4.0%
	Daily	21.3%	18.8%	-4.1%
Marketing restrictions	Non-Daily	11.9%	11.1%	-1.7%
	Daily	21.3%	19.3%	-1.7%
**Females**				
**Scenarios**	**Type of smokers**	**2002**	**2018**	**Relative difference in 2018**
Counterfactual*	Non-Daily	4.2%	4.1%	-
	Daily	6.9%	6.0%	-
Status quo**	Non-Daily	4.2%	3.0%	-27.3%
	Daily	6.9%	4.4%	-26.7%
Cigarette Price	Non-Daily	4.2%	3.5%	-16.1%
	Daily	6.9%	5.1%	-15.5%
Smoke-free air laws	Non-Daily	4.2%	4.0%	-2.8%
	Daily	6.9%	5.9%	-2.8%
Media campaigns	Non-Daily	4.2%	4.1%	-1.2%
	Daily	6.9%	6.0%	-1.2%
Cessation treatment policy	Non-Daily	4.2%	4.0%	-2.2%
	Daily	6.9%	5.9%	-2.2%
Health warnings	Non-Daily	4.2%	4.0%	-4.1%
	Daily	6.9%	5.8%	-4.1%
Marketing restrictions	Non-Daily	4.2%	4.1%	-1.7%
	Daily	6.9%	5.9%	-1.7%

* The counterfactual is estimated by keeping all policies at their 2002 levels.

** The status quo is obtained incorporating policies implemented between 2002 and 2018.

+ Relative differences are calculated as the percent difference in the smoking prevalence with a particular policy or group of policies relative to the counterfactual smoking prevalence.

Under the status quo, *SimSmoke* projects daily smoking prevalence in 2018 at 14.6% for males and 4.4% for females and nondaily at 8.2% for males and 3.0% for females. Compared to the counterfactual, the *SimSmoke* status-quo projects that the daily smoking prevalence in 2018 is reduced, in relative terms, by 25.6% for male and 26.7% for females, and that the nondaily smoking prevalence is reduced by 26.9% for males and 27.3% for females.

By individual policy for the year 2018, cigarette price increases are projected to reduce daily smoking rates by 14.4% for males and 15.5% for females and to reduce nondaily rates by 16.0% for males and 16.1% for females. Smoke-free air laws yield relative reductions of 2.7% for male and 2.8% for female daily smokers and relative reductions of 3.2% for males and 3.4% for females for nondaily smokers. Other policies yield similar estimated effects for both genders and daily and nondaily smokers: 4.1% for health warnings, 2.1% for cessation treatment policies, 1.7% for marketing restrictions, and 1.1% for tobacco control campaigns. As a percent of the total reduction in daily and nondaily rates due to policies, *SimSmoke* attributes 56% to increased cigarette prices, 15% to health warnings, 11% to smoke-free air laws, 8% to cessation treatments, 6% to marketing restrictions, and 4% to tobacco control mass media campaigns.

### Sensitivity analyses

To further validate *Mexico SimSmoke* projections of daily and nondaily smoking, we perform two sensitivity analyses, one assuming half of the original policy effect sizes for nondaily smokers and a second one assuming half of the original effect combined with using the100-cigarettes lifetime screen for defining current smoking prevalence (S1 Report). Assuming half of the original effect, daily smoking projections are unaffected, but *SimSmoke* nondaily smoking projections in 2016 are closer to those observed with full policy effects, but still below survey estimates (model: 10% vs. survey: 18.4% for males; model: 3.8% vs. survey: 6.0% for females) (S2.2 Table in S1 Report). The relative reduction in smoking prevalence from all policies compared to the no policy counterfactual is reduced from about 22% to 17% for nondaily smokers (S3.2 Table in S1 Report).

Using the 100-cigarettes lifetime screen for defining current smoking prevalence in combination with half of the policy effect, the relative prediction errors for daily smokers are roughly the same compared to analyses without the screen. Applying the assumption of half the policy effects for nondaily as daily smokers and using the prevalence estimates with the 100 cigarettes lifetime screen, the relative underestimation of nondaily smoking from the model is still large for nondaily smokers by 2016 (model: 5.2% vs. survey: 12.3% for males; model: 1.4% vs. survey: 3.6% for females) (S2.4 Table in S1 Report) In that case, the relative reduction due to policies is still about 17% (S3.4 Table in S1 Report).

## Discussion

Previous *SimSmoke* models, including the earlier Mexico model [11, 35–38], have generally validated well against country-specific smoking prevalence. However, the updated *Mexico SimSmoke* does not validate well against population trends, even after distinguishing daily and nondaily smokers. By 2016, *SimSmoke* underpredicts the reduction in daily smoking compared to survey estimates. *SimSmoke* also predicts a decline in nondaily smoking rates, but surveys show an increasing trend.

While simulation models are generally evaluated in terms of their ability to predict actual behavior, the failure of this model to predict well suggests areas for future research. In particular, the poor validation of the model points toward the need for a better understanding of nondaily relative to daily smoking. One potential cause for poor predictions may be related to the use of the 100-cigarettes lifetime screen. However, applying the screen did not improve model validation for nondaily smokers. A recent study [3] observed that Mexico’s nondaily smoking prevalence without the 100-cigarettes screen for ages 15–24 was nearly 70% greater than with the screen, and differences were observed even among older age groups. Similar variations using the 100-cigarettes screen have been observed in a US study [39].

Attention should also be directed at the impact of policies on daily and nondaily smokers. *Mexico SimSmoke* predicts more than a 35% reduction in smoking potentially due to policies implemented up to 2018. However, with the reductions understated for daily smokers and overstated for nondaily smokers, the results should be interpreted with caution. While the model predictions improve slightly by assuming half the effect of policies on nondaily compared to daily smokers, *SimSmoke* still fails to predict the observed increase in nondaily smoking. Indeed, when combining daily and nondaily smokers, *Mexico SimSmoke* still overpredicts the reductions between 2002 to 2016 in overall smoking prevalence relative to surveys (males: 26% model vs. 13% survey, females: 27% model vs. 18% survey).

While the effects of policies projected by *Mexico SimSmoke* are likely biased towards daily users, the results still highlight the potential beneficial effect of different policies. Price increases are projected to have the greatest effect on smoking. However, increased cigarette taxes may have led to reductions in the number of days smoked, i.e., substitution of daily for nondaily smoking, as well as a reduced effect on nondaily smokers. A previous study [40] found that 62.1% of Mexican nondaily and 25.9% of daily smokers purchased single cigarettes and paying more than double compared with those purchasing cigarette packs, suggesting that nondaily smokers purchasing single cigarettes may be less responsive to price. Smoke-free air law policies may also have a weaker effect on nondaily users, with nondaily smokers deciding to smoke only on weekends and to avoid smoke-free restaurants and bars.

Cessation treatment policies, such as health care provider brief interventions, may also have less impact on nondaily than daily smokers. Traditionally, nondaily smokers are frequently considered as low nicotine dependent users and assumed to have less incentive to quit. As a result, health personnel are less likely to advise cessation [5]. However, evidence has shown that nondaily smokers may have a similar or greater physical and psychological dependency and lower intentions to quit than daily cigarette users [9, 41, 42]. Nondaily smokers also perceive lower health risks than daily smokers [42, 43], suggesting that media campaigns and cigarette pack warnings may have a limited effect on this group. To address these issues, media campaigns and cessation treatment may need to employ strategies that are tailored to reach nondaily smokers.

*SimSmoke* models are generally used to calculate smoking attributable deaths and long-term projections. If policies remain at 2018 levels, *Mexico SimSmoke* projects a reduction in the prevalence of nondaily smoking of 38% by 2060 (8.2% to 5.8% for males and 3.0% to 2.1% for females from 2018 to 2060) with a similar reduction in prevalence for daily smokers (14.6% vs. 10.8% for males and from 4.4% to 2.7% for female) (S3.1 Table in S1 Report) and 1,186,165 fewer deaths occurring from 2018 to 2060 from which 14% will be from nondaily users (S4.1 Table in S1 Report). Due to the poor predictability of the model, these results may be underestimating the impact of nondaily users in the burden of smoking in Mexico; however, they emphasize the differential effect of the control policies between daily and nondaily users.

Results from our simulation model depend on the data, input parameters, and assumptions underlying the model. The model’s failure to predict the increasing patterns of nondaily usage may reflect the *Mexico SimSmoke* model’s limitations. Daily and nondaily smoking prevalence estimates were obtained from ENA 2002, but it was not until after GATS 2009 that the Mexican national health surveys started to use a standardized measure to collect nondaily prevalence (i.e., “Do you currently smoke tobacco on a daily basis, less than daily, or not at all?”). Differences in the definition of nondaily users between the data used for model input and validation may contribute to the failure in our validation. Another limitation is that projections are based on the cessation rates used in the model. While we assume the same quit rates for both, recent studies found higher quit rates among nondaily than daily [44], as might be expected due to reduced nicotine intake. However, higher quit rates would have led to an even greater decline in nondaily smoking than our projections rather than the observed increase by surveys. Furthermore, nondaily users’ cessation behaviors are variable [45] and more study is warranted on the quitting and switching patterns of daily and nondaily smokers. In addition, the policy effects for nondaily smokers used in this modeling exercise are tentative. We apply sensitivity analyses by reducing the effect of some policies in order to reflect potentially lower policies impacts on nondaily smokers, but further ranges should be explored.

Another limitation of our model that could be a source of discrepancy between model projections and observed behavior may be the failure to consider the evolution of nicotine delivery products. The rapid uptake of new nicotine delivery products may have influenced tobacco use patterns in such a way as to undermine cigarette-oriented tobacco control policies. For instance, cigarettes with flavor capsules have rapidly gained market share in countries like Mexico [46], where they were introduced soon after the General Tobacco Control Law was implemented. Furthermore, despite a ban on e-cigarette sales in Mexico, e-cigarette use has rapidly grown, particularly amongst youth and adult smokers [47]. However, trends toward increased nondaily smoking began before e-cigarettes were introduced.

Better information is needed to understand the trajectories and differential effects of implemented policies on daily and nondaily smokers. Our study casts light on the shortage of surveillance and policy evaluation data regarding nondaily smokers’ behaviors. With tobacco usage behaviors becoming more complex, the lack of nondaily data and understanding represents a barrier to effectively regulating these products. The World Health Organization [48] and the Institute of Health Metrics and Evaluation [49] have focused on overall current or daily smokers, and often do not report nondaily smoking. The reporting of nondaily smokers’ prevalence by leading international agencies would encourage countries to follow their lead. There already exist standardized tools to monitor nondaily tobacco use. The Global Tobacco Surveillance System (GTSS) collects data on tobacco surveillance through four different surveys and effectively reports nondaily (occasional) data for 22 countries. As part of the GTSS, GATS provides a subset of standardized tobacco monitoring measures that can assist other countries in collecting data on smoking prevalence and tobacco control policies indicators. Systematizing the use of this set of questions in national surveys would be an important first step towards improving surveillance [50]. Further, to better capture nondaily trends and users’ characteristics, the nondaily use definition could be expanded beyond reporting “occasional use” or “consumption in one or more days in the past 30 days” by including frequency and intensity measures.

While the heterogeneity and stability of nondaily users (e.g., frequent and infrequent, exclusive, or multiproduct users) behaviors makes it difficult to estimate the effect of a policy on this group, it is important to characterize the response of nondaily users to regulations and apply these characterizations to future tobacco control policies development [51]. This need is particularly relevant for the Mexican and other Hispanic/Latino populations which have shown a higher prevalence of nondaily use than other populations [52–54], and especially for Mexicans who are more likely to be nondaily smokers than other Hispanic/Latino individuals [55]. Although this behavior may in part be attributed to social influence, it has also been observed that the presence of a gene variation in Hispanic/Latino smokers has potential influence in their predisposition towards low intensity nondaily use [56].

As one of the countries with the highest proportion of nondaily smokers, Mexico’s particular tobacco prevalence and control panorama calls attention to the relevance of including nondaily monitoring indicators as part of the policy evaluations. Nondaily smokers are an important contributor to the tobacco-associated disease burden [57]. Mexico’s overall tobacco prevalence has remained stable since 2009 (around 80%) with a reduction in prevalence from daily smokers (from 8.6% to 7.5% in 2016 [2]. However, Mexico’s smoking-attributable deaths have increased from 51.6 thousand in 2017 (7.5% of total deaths) to 58.2 thousand in 2019 (8.1% of total deaths) [58] due to the increase in nondaily prevalence. Other countries have started to show similar tobacco trends [59]. Brazil, a high-achieving tobacco control country, has seen a reduction in the rate of decline in smoking prevalence [60, 61], with an increase in nondaily and in illicit cigarette use [62], similar to the conditions in Mexico which have contributed to the rise in nondaily use. Mexico’s lack of surveillance of nondaily use combined with the low enforcement of implemented policies also characterizes other low and middle-income countries [63]. Public health researchers need to consider systematizing the collection of the nondaily smoking data to monitor and prevent future scenarios like the one observed from Mexico.

Addressing the needed improvements in surveillance also goes hand-in-hand with efforts to reassess and update tobacco control regulations. Fifteen years ago, Mexico was one of the first countries in America to ratify the WHO-FCTC treaty. Since then no major changes to the policies have been made [1]. Despite the lack of major policy changes, previously implemented policies were reducing the smoking problem in Mexico, as observed by the validation of our previous *Mexico SimSmoke* models [11, 12]. However, with the changes in population smoking patterns in recent years, the implemented policies appear to no longer address Mexico’s population smoking patterns and needs. By not moving forward, there is the risk of losing the reduction in smoking prevalence and the requisite public health benefits that have been obtained in previous years [3, 64].

Despite a failure to capture actual trends, *Mexico SimSmoke* helps identify gaps in surveillance and policy evaluation. Nondaily use has become an important public health problem. The results presented here demonstrate a need to better capture the dynamic relationship between daily and nondaily smoking and how policies affect these two groups. Mexico’s nondaily smoking has increased, and future policy will need to better target nondaily smokers. More generally, nondaily smoking is common in many countries, and further advances in tobacco control will require a better understanding of this group’s characteristics.

## Supporting information

S1 Report (DOCX)
