# Supplementary material for: The Mexico SimSmoke tobacco control policy model: Development of a simulation model of daily and nondaily cigarette smoking
Source: PLoS One. 2021 Jun 21;16(6):e0248215. doi: 10.1371/journal.pone.0248215 (PMC8216521; doi:10.1371/journal.pone.0248215)
Supplement: S1 Report — (DOCX) [file pone.0248215.s001.docx]

***The Mexico SimSmoke*** **Tobacco Control Policy Model: Development of a**

**simulation model of daily and nondaily smoking**

**Supplementary Report**

Luz María Sánchez-Romero, MD, MSc, PhD

Lombardi Comprehensive Cancer Center, Georgetown University, Washington, D.C

Luis Zavala-Arciniega, MD, MSc

Tobacco Research Department, National Institute of Public Health, Cuernavaca, Mexico

Luz Myriam Reynales-Shigematsu MSc, PhD

Tobacco Research Department, National Institute of Public Health, Cuernavaca, Mexico

Belen Saenz de Miera-Juárez MSc, PhD

Tobacco Research Department, National Institute of Public Health, Cuernavaca, Mexico

Zhe Yuan, MS

Lombardi Comprehensive Cancer Center, Georgetown University, Washington, D.C

Yameng Li, MS

Lombardi Comprehensive Cancer Center, Georgetown University, Washington, D.C

Yan Kwan Lau, MPH

Department of Epidemiology, University of Michigan School Public Health, Ann Arbor, MI

Nancy Fleischer, PhD

Department of Epidemiology, University of Michigan School Public Health, Ann Arbor, MI

Rafael Meza, PhD

Department of Epidemiology, University of Michigan School Public Health, Ann Arbor, MI

James F. Thrasher, PhD

Department of Health Promotion, Education & Behavior, Arnold School of Public Health, University of South Carolina, Columbia, USA

David T. Levy, PhD

Lombardi Comprehensive Cancer Center, Georgetown University, Washington, D.C

**October 2020**

**SUMMARY**

This report describes in detailed the development of an updated version of *Mexico SimSmoke* simulation model that examines the potential effect of tobacco control policies on daily and nondaily smoking prevalence in Mexico. This model was based on the established *SimSmoke* tobacco control policy simulation model, which has been previously developed and validated for the US and other nations. A previous *Mexico SimSmoke* model was adapted by using population size, smoking rates, and tobacco control policy data by 2018 for Mexico.

*Mexico SimSmoke* assesses, individually and in combination, the effect of seven types of policies: cigarette taxes, smoke-free air laws, tobacco control campaigns, marketing bans, health warning labels, cessation treatments, and youth access policies. Besides considering the effect of past policies, it presents the benefits of a more comprehensive tobacco control strategy. The model also explores how the effect of these policies depends on the population composition, the existence of other policies, and the length of time of implementation.

*Mexico SimSmoke* validated relatively well for female daily smoking, but poorly for male daily and male and female nondaily smoking. Between 2002 and 2016, *SimSmoke* predicted a decline in nondaily smoking rates for males and females, while survey rates instead rose. The increase in nondaily smoking occurred primarily among male ages 15-24 and 25-44 during 2002-2012 and 15-24, 25-44, and 45-65 during 2012-2016 and observed for female ages 15-24, 25-44, and 45-65 during 2002-2012 and 15-24 and 25-44 during 2012-2016. *SimSmoke* also under-predicted the reduction in male and female daily smoking prevalence during 2012-2016.

*Mexico SimSmoke* shows that past policies have been relatively effective at reducing daily, but not nondaily smoking. The predicted reductions in both daily and nondaily smoking by *SimSmoke* were largely due to policy changes from 2002-2018. Tax policies accounted for about 60% of the reduction due to policies, followed by health warnings, cessation treatment policies, smoke-free air laws, and tobacco control spending. We also projected the effect of implementing stricter policies than those already in place. Increasing the cigarette excise tax to 60% of price yielded a 7.5% relative reduction in smoking prevalence, while increasing the tax to 70% yielded a 20% prevalence reduction. Increased tobacco control spending was projected to reduce prevalence by 8%, while cessation treatments yielded a 5% reduction. Mostly through better enforcement, more widespread smoke-free air laws were projected to reduce smoking prevalence by 4% and marketing restrictions by 5.5%.

*SimSmoke* also helps to identify gaps in surveillance and points to the lack of evaluation schemes that could further show the effectiveness of tobacco control policies in Mexico. Current policies, such as media campaigns and cessation treatment policies, need to be better directed at nondaily smokers, and smoke-free air laws and marketing restrictions need to be better enforced. The model did not consider other policies that may also target nondaily smokers, such as restrictions on flavored capsules and the sale of single cigarettes. In general, future research should consider measurement issues related to the prevalence of smokers, especially nondaily smokers, and the factors influencing their trends.

**INTRODUCTION**

Globally, it is estimated that 7 million deaths each year are attributable to tobacco use, with trends that show they could reach 10 million deaths per year by 2030 ([1](#_ENREF_1)). In response, the World Health Organization (WHO) has set out the Framework Convention for Tobacco Control (FCTC), a treaty that reaffirms the right of the population to health and promotes strategies to prevent and reduce the consumption of addictive substances like tobacco ([2](#_ENREF_2)). Based on this treaty, WHO MPOWER measures were introduced to define a set of policies to assist countries in achieving their commitments to reduce tobacco demand ([3](#_ENREF_3)). In 2004, Mexico ratified the FCTC treaty and since then, has adopted stronger advertising restrictions, smoke-free laws in some states, stronger health warnings, and increased cigarette taxes.

Population tobacco control policies, including higher cigarette taxes, smoke-free air laws, advertising bans, media campaigns, and cessation treatment policy, have been shown to be effective in reducing adult smoking rates, especially when combined as a comprehensive strategy ([4-7](#_ENREF_4)). These policies not only reduce smoking initiation, but also lead current smokers to quit. Quitting can halt or even reverse many of the health problems associated with smoking ([8](#_ENREF_8), [9](#_ENREF_9)). MPOWER suggests that each nation should impose taxes on cigarettes that constitute 75% of the retail price, implement and enforce comprehensive smoke-free indoor air laws and advertising/marketing restrictions, require large, bold and graphic package health warnings, provides broad access to cessation treatments, and implement well-funded tobacco control campaigns.

Statistical studies generally examine the effect of only a limited number of policies [e.g., Hu ([10](#_ENREF_10), [11](#_ENREF_11)), Farrelly ([12](#_ENREF_12))], because the ability to distinguish their effects on smoking rates is limited. However, simulation models are able to combine information from different sources to provide a useful tool for examining how the effects of public policies unfold over time in complex social systems ([13](#_ENREF_13), [14](#_ENREF_14)). Simulation models examining the effect of tobacco control policies have been developed by Mendez and Warner ([15](#_ENREF_15), [16](#_ENREF_16)), Tengs et al. ([17-19](#_ENREF_17)), Ahmad ([20-22](#_ENREF_20)) and Levy et al. ([14](#_ENREF_14)). The *SimSmoke* model by Levy et al. simultaneously considers a broader array of public policies than other models and has been validated in many countries ([23-31](#_ENREF_23)) and states ([32-38](#_ENREF_32)).

*SimSmoke* has been previously adapted to Mexico using population, smoking rates, and tobacco policy data specific to the country ([39](#_ENREF_39), [40](#_ENREF_40)). *Mexico SimSmoke* assesses the effect of cigarette taxes, smoke-free air laws, tobacco control campaigns, advertising bans, health warnings, cessation treatment policies, and youth access laws on smoking prevalence and smoking-attributable deaths (SADs). This previous version of *Mexico SimSmoke* considered the role of policies through the year 2012 and found that smoking prevalence had been reduced by about 30% as a result of policies implemented 2002 and 2011([40](#_ENREF_40)). The model generally predicted trends well when compared to surveys of smoking prevalence ([40](#_ENREF_40)), but the model did not distinguish daily from nondaily smokers. Studies have found that more than half of smokers in Mexico smoke nondaily and are playing an increasing role in smoking prevalence ([41](#_ENREF_41), [42](#_ENREF_42)).

This report describes the development of a new version of *Mexico SimSmoke*. We updated the model to examine the role of Mexican smoking-control policies from 2002 through 2018 and projected trends from 2019 to 2060. This model designates distinct outcomes for daily and nondaily smokers. This new version was validated against smoking prevalence from different Mexican national health surveys from 2009 through 2016.

**METHODS**

*Mexico SimSmoke* includes population, smoking, smoking-attributable death, and policy modules ([14](#_ENREF_14), [43](#_ENREF_43), [44](#_ENREF_44)). It uses 2002 as the baseline year, because of the availability of the large-scale survey. In addition, smoking prevalence had been relatively stable prior to 2002 ([45](#_ENREF_45)), and before FCTC implementation in 2004 and the implementation of most major tobacco control policies.

We assume a discrete time, first-order Markov process to project future age and gender-specific population growth and smoking rates from 2002 through 2060. Briefly, total population growth evolves through a predetermined population before age 15, deaths, and immigration. Smoking prevalence evolves through smoking initiation, cessation, and relapse, but are modified through changes in the level of tobacco control policies.

By applying a method similar to standard attribution measures ([46](#_ENREF_46), [47](#_ENREF_47)), smoking-attributable deaths were estimated using total population, current and former smoking prevalence, and total mortality risks of current and former smokers relative to never smokers. A detailed description of the mathematical equations used is found in Appendix 1.

**Population**

Population, mortality, and immigration data for 2002-2050 by single age (0 to 85+) and gender were obtained from the Mexico National Council of Population (*Consejo Nacional de Población*, CONAPO) ([48](#_ENREF_48)). Instead of estimating new births using fertility rate in classic SimSmoke model, we used the CONAPO population data from birth up to age 14 for all years as never smokers, assuming no smoking occurs before age 14 ([39](#_ENREF_39), [40](#_ENREF_40)). Evolution of the population age 15 and above incorporated mortality.

Mortality rates were obtained by dividing the total number of deaths for each age, gender and year by the corresponding mid-year population. To capture the decrease in mortality rates, baseline mortality rates in 2002 were adjusted relative to 2002 (ratio of future mortality to mortality in 2002) at later years, instead of using constant mortality rates as in the previous *Mexico SimSmoke*. We applied a linear interpolation to the male’s mortality rates between 2007 and 2017 to overcome an observed 20% increase in the number of deaths among males age 10-45 in the period 2008-2016. Figure 1 shows the mortality rates for males before and after adjustment.

**Figure 1: Males’ mortality rates before and after interpolation, 2002-2050, CONAPO**

The projected population was also adjusted by single age, gender and year specific net international immigration rates for all years. Figure 2 shows the reported immigration rates for 2002 from CONAPO. On a per capita basis, in 2002, net immigration rates increased from 0.2% in age 0-10 and to 1.6% for age 22-23. They slowly decreased to 0.2% at age 50 and remained stable afterward for males, increased from 0.2% in age 0-10 to 0.8% in age 20-25, and dropped to 0.2% at age 40 and remained at about 0.2% for older ages for females (Figure 2).

**Figure 2. Net immigration rates in Mexico by gender, 2002, CONAPO**

Figure 3 shows net immigration rates by age from 2002 to 2050. The data showed that overall net immigration rates remained constant from 2002 to 2004, but changed for all ages starting in 2005. For males and females ages 0-8, rates fell to about -0.1% in 2007-2016 and increased to half of its 2002 value thereafter. For ages 9-50, both genders fell to one-third their 2002 values in 2007-2016 and returned to half of that value thereafter. For ages 51+, both gender net immigration rates almost drop to zero in 2007-2016 but returned to half of their 2002 values after 2016.

#### **Figure 3. Trends in net immigration rates by age and gender, 2002-2050, CONAPO**

To validate population projections for both genders, we compared the projected population from the model to data available from CONAPO for the last year of tracking period (2018). Figure 4 shows the differences in relative terms. The relative difference through age 14 is zero because we used CONAPO population data before that age. A relative difference of less than 1% was obtained between ages 21-30 and a less than 1% relative difference was obtained for ages 30 to 68 for males and 30 to 70 for females. We observed that increasing overestimation occurred after age 70 for both genders, from about 1% to nearly 9% for males and 2% for females at age 85+. While we did not isolate the discrepancy at older ages, it may pertain to the model failing to account for tobacco-related deaths. Overall, the population module predicted well in 2018, and the male (female) population ages 15 and above was 43,963,121 (47,740,409) compared with 43,805,808 (47,697,770) reported in CONAPO, overestimated by 0.36% (0.09%) in relative terms. To project through the year 2060, population, mortality, and immigration rates are assumed at the same level for 2051-2060 as in 2050.

**Figure 4. Relative difference (%)* in model projection vs CONAPO population, 2018**

Note: Relative difference was measured as (Model - CONAPO)/CONAPO.

**Smoking Prevalence**

**Baseline prevalence estimates**

The baseline current daily and nondaily current smoking, former smoking and never smoking prevalence were obtained primarily from the National Addictions Survey *(Encuesta Nacional de Adicciones*, ENA) 2002 ([49](#_ENREF_49)). ENA is a Mexican nationally representative probabilistic, randomized and multistage household survey of population ages 15 to 65 years old that distinguished between daily and nondaily smokers. Trained personnel collected data via a face-to-face interview. Smoking information was collected from a total of 4,619 males and 5,799 females. Each survey participant was classified as a current, former or never smoker using the questions, if they had ever smoked tobacco in their lifetime, and if they had smoked in the past-30 days. A never smoker was defined as having never smoked in his/her lifetime. A current smoker was defined as an individual who smoked in his/her lifetime and smoked during the past-30 days. Current smokers were further distinguished as nondaily or daily smokers based on whether they smoked daily in the past-30 days. A former smoker was defined as those who had smoked in his/her lifetime, but not in the past-30 days. Former smokers were further stratified according to the number of years quitting, in the following categories: < 1 year, 1-2 years, 3-5 years, 6-10 years, 11-15 years or ≥16 years. The prevalence of nondaily, daily, and former smokers were stratified in nine age groups: 15-17, 18-21, 22-24, 25-29, 30-34, 35-44, 45-54, 55-64, and 65.

The ENA questionnaire also asked if the individual smoked 100 cigarettes during his/her lifetime. In the previous *Mexico* *SimSmoke* ([39](#_ENREF_39), [40](#_ENREF_40)), we limited ever smokers to those that met the 100 cigarettes screen. However, the screen was not used in the current model, because of a concern that many smokers may have been omitted due to the large percentage of nondaily smokers in Mexico ([41](#_ENREF_41), [42](#_ENREF_42), [50](#_ENREF_50)). For comparison, current and former smokers who smoked at least 100 cigarettes lifetime are also considered as the baseline prevalence. Appendix 2 contains a comparison of the two methods, which shows that the 100 cigarettes screen leads to particularly large differences in the rates for younger and nondaily smokers, with relative differences between ever smoker measures as large as 70%. Comparing prevalence estimates with and without the screen using ENA 2002 data, we found that 4.5% (1.5%) of male (female) smokers aged 45-64 claimed that they did not smoke 100 cigarettes, but admitted to smoking in the past-30 days, with much larger percentages at younger ages.

Due to unavailable data on the prevalence for former smokers, the ratio between the prevalence of current smokers with and without screening by age group and gender are used to scale the prevalence of former smokers without screening for substitution.

ENA 2002 did not contain information for the population older than 65 years. Smoking prevalence for that age group was obtained from the National Health and Nutrition Survey, Mexico (*Encuesta Nacional de Salud y Nutricion*, ENSA/ENSANUT) 2000. ENSA 2000 is nationally representative, using probabilistic multistage cluster sampling collected from 54,078 individuals aged 15 and above. This survey only collected detailed smoking data from those who met the 100 cigarettes lifetime screen.

Current smokers in ENSA 2000 were those individuals that responded affirmatively to the question if they “have smoked 100 cigarettes during their lifetime?” We then separated those individuals in daily and nondaily smokers according to the response to “how often do you smoke?” We defined former smoker as those who answered no when asked, “Do you currently smoke?” Since ENSA 2000 measure smoking status differently from ENA 2002, we compared smoking rates for the 45-64 age group from the two surveys to assess potential differences. We compared the current smoking prevalence of age group 45-64 with the screen in ENSA 2000 (31.5% for males and 9.4%) separately with that in ENA 2002 with (30.1% for males and 8.8% for females) and without the screen (34.6% for males and 10.3% for females). With the 100 cigarettes screening, the male (female) prevalence decreased by 4.4% (6.4%) in relative terms and increased by 9.8% (9.6%) without the screening in ENA 2002. Since the difference in prevalence with and without a screen is small and older smokers are more likely to meet the 100-cigarette screen, no adjustment was made to the ENSA 2000 prevalence for ages 65 and above.

For current daily and nondaily smokers, the age-group prevalence obtained from ENSA 2000 and ENA 2002 were transformed to prevalence estimates by single age to be used as inputs for *SimSmoke*. Beginning with smoking rate by age group, we assumed that rate was the prevalence of the corresponding mid-age of that group (e.g., age 16 for 15-17, age 19 for 18-21, age 23 for 22-24, age 27 for 25-29, and age 32 for 30-34, age 40 for 35-44, age 50 for 45-54, age 60 for 55-64, age 73 for 65 and above). We assumed no smoking prevalence for individuals below age 15, and linear-interpolated the smoking rates between age 14 (0% current smokers) and the mid-point ages (16, 19, 23, 27, 32, 40, 50, 60, 73) to estimate single age prevalence for ages 15-72. We then smoothed the rate for every single age by a 3-year moving average for age 15-24 and a 5-year moving average for 25-83. Smoking prevalence for ages 74 to 80 was estimated by extrapolating the prevalence trends for age 60 to 73, and the prevalence from 80 years onwards was assumed to be constant.

Former smokers were classified, according to the number of quitting years in five groups: ≤1, 2-5, 6-10, 11-15, and ≥16 using the same procedure as applied for current smokers to determine by single age. To estimate the prevalence of former smokers by quit years, we first used the percentages of quit-year sub-groups (<1, 1-2, 3-5, 6-10, 11-15, and 16+ years) among total former smoker population from ENA 2002 to divide the total prevalence of former smokers into the same five quit-year groups. We assumed the same ratios for single ages within an age group, and the ratio of age group 55-64 was assigned to every single age at 65 and above. We then used the inferred prevalence of former smokers by quit-year group and redistributed with equal prevalence for former smokers who quit 3, 4, or 5 years. Finally, we applied the ratio of current daily to nondaily smoker to total former smoker prevalence to obtain daily and nondaily smoker prevalence for all quit years. Since the sample size of ENA 2002 was too small to detect former prevalence by years quit, the accuracy of our estimated percentage by quit-year groups among former smokers was assessed by comparing the obtained percentage by quit-year from ENA 2002 to the percentage data from 1993 Current Population Survey- Tobacco Use Supplement (CPS-TUS) ([51](#_ENREF_51)) by age group (25-44 and 45-64). Our results showed that the percentage by quit-year among former smokers from the two datasets were similar for both age groups and genders. Hence, ENA 2002 was adopted input for former smoker prevalence.

Since *SimSmoke* model does not distinguish the mortality rates of former smokers from current or never smokers before age 35, we modeled former smokers starting from age 30 by quit-year in order to take advantage of the former smoker rates averaged over ages 25-35 (30.5% for male and 16.2% for females), with the mathematical derivations in Appendix 1. Up until age 30, we do not distinguish initiation, cessation, and relapse rates, because those rates are generally unstable. Instead, as described below, we modeled the net initiation rates into daily and nondaily smoking, which incorporates initiation, cessation relapse and switching between daily and nondaily smoking. In later years, first-year former smokers are determined by the cessation rate.

After calculating current and former prevalence by age and gender, the remaining population was assumed to be never smokers, i.e., 1 - (daily smoker prevalence + nondaily smoker prevalence + former smoker prevalence for all quit years).

**Transitions**

*Mexico SimSmoke* classifies individuals as never smokers from birth. After age 14, individuals could either initiate smoking or remain as never smokers, if they do not die. Once becoming a smoker, individual may quit. Former smokers may return to their past smoker status through separate relapse rates, which depends on years quit.

Due to empirical challenges in measuring initiation and cessation, and in order to ensure stability and internal consistency of the model, baseline initiation rates at each age were measured as the difference between the baseline-smoking rate (2002) at each age and the baseline-smoking rate at previous age. Initiation rates of nondaily and daily smokers were developed from their respective baseline smoking rates in 2002. Based on the ages over which there were increases in the daily smoking prevalence for both genders, we allowed for initiation through age 28 for both males and females. Because we do not explicitly allow for switching from daily to nondaily smoking, we did not distinguish the initiation cut-off rate for daily and nondaily smokers in order to allow for nondaily smokers switching to daily smoking.

The cessation rate during the past year was obtained using ENA 2002 data, calculated as the number of former smokers who quit during the last year as a percent of smokers one year ago(current smokers + those who quit in the last year). In addition, we considered cessation and relapse data from the International Tobacco Control (ITC) surveys ([52](#_ENREF_52), [53](#_ENREF_53)) to calculate average cessation rates across both males and females, and found those rates were about 7.8% over the period 2007 through 2012. Allowing for the effect of policies since 2002, the ENA 2002 yielded cessation rates similar to ITC estimates. The rates for males ranged from 6%-7% at ages 28- 46, dropping to 4% at ages 47-64, increasing to 6% after age 64 for males, and for females remaining at 8% for ages 25-36, ranging from 4%- 7% at ages 37-53, and remaining at 4.6% and 6% at age 54 and above. Although there was some indication of higher cessation rates for nondaily than daily smokers in both the ITC,([53](#_ENREF_53)) we assumed the same cessation rates for daily and nondaily smokers, because nondaily smoker cessation rates may have been inflated due to what might be considered more short-term quitting.

For relapse rates (for the first through 5 years), we examined ITC data by city, which were found to be close to those of the US ([9](#_ENREF_9), [54-57](#_ENREF_54)). Consequently, we used US relapse rates for both male and female smokers differentiated by quit years (quit-year sub-groups includes <1, 1-2, 3-5, 6-10, 11-15, and 16+ years). The same estimated relapse rates by gender were assumed for daily and nondaily smokers.

## Smoking-Attributable Deaths

Smoking-attributable deaths (SADs) were determined by excess smoking mortality risks for current and former smokers by gender, which were calculated from the difference between estimated mortality risk of current (or former) smokers and mortality risk of never smokers. Death rates were first calculated by age, gender and smoking categories (never, nondaily, daily, and former smoker groups) using overall mortality rates from 2002 to 2050, 2002 smoking rates, and a set of relative mortality risks.

Since the relative mortality risk estimates of smoking from Mexico were not sufficient to capture risks for women, we use relative risk estimates from the US Cancer Prevention Study II ([58](#_ENREF_58)). Doll and Peto reported similar relative risks for British doctors ([59](#_ENREF_59), [60](#_ENREF_60)). Similar risks have also been used in previous Mexico studies ([61-63](#_ENREF_61)). For daily smokers, relative mortality risk were set at 2.2 through age 59 and of 2.0 for age 60 and above for males. For females, risks were set at 2.0 through age 59 and at 1.9 for age 60 and above. For US former smokers, relative risks was modeled as declining as age increases ([9](#_ENREF_9), [58](#_ENREF_58), [64](#_ENREF_64)). Based findings in a recent study ([65](#_ENREF_65)) and a recent review ([66](#_ENREF_66)), we estimated that nondaily smokers have a 65% excess mortality risk of daily smokers. Nondaily risks were calculated as exp (65%*ln(relative risk of daily smokers)). The risks of former nondaily smokers were assumed to fall at the same rate as for former daily smokers.

The number of daily smokers at each age was multiplied by the difference in death rates between daily smokers and never smokers to obtain the number of excess deaths due to being a daily smoker. We applied the same method for nondaily smokers and former smokers by quit-year group. The results for each smoking group were summed to estimate the total number of SADs.

## Tobacco Control Policies

The policy effect sizes in *SimSmoke* are based on literature reviews ([7](#_ENREF_7), [67](#_ENREF_67)) and the advice of an expert panel, and are defined in terms of percentage reductions in smoking prevalence, initiation and cessation rates. Reductions are applied to the smoking prevalence in the year of policy implementation, and applied to initiation and cessation rates in future years, unless otherwise specified, if the policy was sustained. In the absence of synergies, the effect of a second policy implemented simultaneously is reduced by (1- the effect of the first policy).

Although Mexico is categorized as a middle-income country, the policy effects used in this study for daily smokers were the same as used for high-income nations, because Mexico’s smoking trends (i.e., the stage in the epidemic) are closer to high-income trends than to middle-income trends. However, effect sizes are based primarily on studies where the population consists mostly of daily smokers. Therefore, we conduct sensitivity analysis 1) assuming the same policy effects for daily and nondaily smokers, and 2) assuming half the policy effects for daily and nondaily smokers. Table 1 provides a detailed description of the policies and effect sizes used in *Mexico* *SimSmoke*.

The effect of a policy is dependent on its initial level (e.g., the incremental effect of a complete worksite ban is less when a nation already has a partial worksite ban). Because changes in policies affect the future path of smoking prevalence in *SimSmoke*, we tracked policy levels from the baseline year, 2002, to the most recent year, 2018. The level of a policy is based on MPOWER Reports 2007-2016 ([3](#_ENREF_3), [68-70](#_ENREF_68)), the [**Tobacco Control Report for the Region of the Americas 2013**](http://www.paho.org/hq/index.php?option=com_docman&task=doc_download&gid=24181&Itemid=270&lang=en) ([71](#_ENREF_71)), and members of the Mexican National Institute of Public Health (INSP).

**Cigarette Excise Taxes**

Cigarette excise taxes are considered one of the most potent tobacco control policies ([7](#_ENREF_7)). To estimate the effect of taxation on our main outcomes, the *SimSmoke* model used an equation to translate tax rates into changes in the package price. The estimated amendments in price were then translated into changes in smoking prevalence through an equation dependent on price elasticities (see Appendix 1) as described in Levy et al. ([72](#_ENREF_72)).

Chaloupka et al. ([73](#_ENREF_73)) found that high-income nations had total price elasticities between -0.3 and -0.5, averaging -0.4, higher than price elasticities for low- and middle-income nations. For Mexico, Waters et al. ([45](#_ENREF_45)) obtained a per capita consumption price elasticity of -0.5 and Saenz de Miera Juarez et al. ([74](#_ENREF_74)) obtained a participation elasticity (decision to smoke) of -0.17 and an intensity (quantity smoked) elasticity of -0.4, yielding an overall elasticity of about -0.6. Because total prevalence elasticities for Mexico were close to those of the US, we use the age-specific prevalence elasticities from US *SimSmoke* ([30](#_ENREF_30)). They are -0.4 for ages 15-17, -0.3 for ages 18-24, -0.2 for ages 25-34, and -0.1 for ages 34 to 64 and -0.2 for ages 65 and above. We assume the same elasticities for daily and nondaily smokers, but also consider half the effects for nondaily smokers since they may pay higher prices from purchasing cigarettes in smaller amounts and may be less sensitive to prices since their purchases are likely to require a smaller share of their income than daily smokers.

Figure 5: Comparison of inflation-adjusted annual cigarette prices, 2000-2017

We used the mean annual real cigarette price for each year. We also incorporate the value added tax, although not specific to cigarettes, since this tax amplifies excise taxes (see Appendix 1). To assess the validity of cigarette price data, we compared the National Institute of Statistics and Geography (*Instituto Nacional de Estadística y Geografía*, INEGI) series with price data from Waters et al. ([45](#_ENREF_45)) for the earlier years (2000-2009) and data from MPOWER for 2008-2016. Both were adjusted to the 2016 price level using the INEGI national consumer price index ([75](#_ENREF_75)). We also compared our estimates with data from the Institute of Health Metrics and Evaluation (IHME) for later years (2011-2016) (Provided by INSP). Our results (Figure 5) indicate that INEGI data was consistent with other sources and appears to better capture yearly price changes. Data on the percentage of excise and value-added tax rate (VAT) (as a percentage of the price to the consumer) were from Waters et al. ([45](#_ENREF_45)) for 2002-2009, and from MPOWER Reports for years 2010 to 2016. They were obtained from world.tax-rates.org/mexico/sales-tax for VAT in 2017-2018. With data available on prices through 2017 and on excise taxes through 2016, we assumed that the price and taxes remained constant in the unavailable years (2018 for price and 2017-2018 for tax).

In summary, the *Mexico* *SimSmoke* real cigarette pack prices increased from $22.17 MXN peso in 2002 to $44.20 MXN peso (1 USD= 19.3 MXN pesos, for 2019), adjusted by Mexican inflation rate. Excise cigarette tax data started at 40.2% from the baseline year (2002) and increased to 53.5% for 2016. The final VAT added to package price after tax was 13.04% from 2002 to 2008 and changed to 13.79% from 2009 to 2018. We assumed that prices and taxes were the same for nondaily and daily smokers.

### Smoke-Free Air Laws

The smoke-free air law module considers smoking bans in four places: worksites, restaurants, pubs and bars, and other public places (i.e., public transportation and other indoor places). *SimSmoke* model distinguished the level of worksite bans as none, low (bans in two of the following three workplaces: health facilities, universities, and government facilities), moderate (ban in indoor offices only), and high (ban in all these worksite areas). Additionally, *SimSmoke* used a score between 0% and 100% to identify the percentage of policy coverage for each of the places. We estimated the percentage of policy ban implementation for restaurants, pubs and bars, and other places based on the percentage of places, at the national level, that have reached a smoke-free level in all indoor areas with a total possible score between 0% and 100%. The effect of smoke-free air laws also depends on enforcement, which ranges from 0 (weak) to 10 (strong), and synergy related to broader tobacco control campaigns (e.g., through greater awareness of the dangers of secondhand smoke).

We based the policy effect sizes for *Mexico* *SimSmoke* on studies of restrictions in private worksites and smoking-free air laws for high-income countries ([7](#_ENREF_7)). For worksites, the effect size used was consistent with that reported by Fichtenberg and Glantz ([76](#_ENREF_76)). With the highest level of enforcement and publicity, a smoke-free air law in *SimSmok*e has an effect size of 6% for a full ban in worksites (4% for a moderate ban and 2% for a low ban), 2% for bans in restaurants, 1% for bans in pubs and bars, and 1% in other public places. We consider effect sizes at half the rate for nondaily smokers to reflect that the impact may be lessened due to less frequent smoking in the places covered. The effects were scaled based on the value of the enforcement of smoke-free air laws from MPOWER reports and the level of publicity from tobacco control campaigns (e.g., the effects are halved in the absence of enforcement and publicity). The worksite laws directly affect only those who work indoors.

According to the 2008 MPOWER report ([77](#_ENREF_77)) and data from the previous *Mexico SimSmoke* ([40](#_ENREF_40)), Mexico had implemented smoke-free air laws at low levels in from 2002 to 2007 for worksites, except in health care and educational facilities, and theaters and cinemas. Since 2008, Mexico City and Tabasco went smoke-free in workplaces, restaurants and bars. In 2008, only 10% of the worksites in Mexico City had smoking designated areas. Smoke-free air laws were adopted in 2011 in Morelos and Zacatecas (increasing the coverage to 18%), in Veracruz in 2012, in Estado de Mexico in 2013, in Nuevo Leon, Baja California and then Baja California Sur, Oaxaca, and Sinaloa in 2014 by, increasing overall coverage to 45%. These changes in coverage were modeled to reflect the percentage of locations with smoke-free as opposed to lesser local laws. In 2008 and 2009, worksites bans had scores of 10% high level and 90% low-level implementation policy. This percentage increased to 18% high level and 82% mid-level policy implementation for 2010-2011. For 2012, the distribution was 20% high level and 80% middle level, and increasing to 40% high level and 60% middle level in 2013, and to 45% high level and 55% middle level for 2014-2016. Restaurants bans and pubs and bars bans had scores of 0% before 2008, but increased to 55% for 2008-2009, 60% for 2010-2012, and 70% from 2013 onwards. We assumed that the policy levels for 2017 and 2018 were the same as in 2016. Bans in other public places were set at 50% for all the modeled years.

To gauge enforcement, we considered actual second-hand smoke exposure. A recent study ([78](#_ENREF_78)) estimated self-reported exposure to smoke at restaurants (31.9%), bars (52.9%), public transportations (26%) and workplace (15.2%) among nonsmokers. In general, the states that had a smoke free law vs. the states that do not have a smoke free law generally showed little difference in exposures, but some states with laws had lower exposure (e.g., Nuevo León had low exposure at bars (40.4%) and restaurants (21.5%) compared to the national average, despite its relatively highest smoking prevalence (22.8%)). Mexico City, the first state that implemented a smoke-free policy, second-hand smoke exposure increased in restaurants between 2011 (25.3%) and 2016 (35.2%) and in bars (36.2% to 50.2%). MPOWER reports rated the enforcement of this policy as 3 on a 10-point scale in 2008, 4 in 2010, 5 in 2012, 3 in 2014, and 6 in 2016. Based on the MPOWER and exposure data, we set the enforcement level of smoke-free air laws at four for all years.

### Tobacco Control Campaigns

The *SimSmoke* tobacco control/media campaign module has three levels of campaigns categories (i.e., high, medium, and low) based on the budget allocated by the country for these activities. The MPOWER reports provide information on the national strategy, goals, number of employees, and overall budget for tobacco control. We described a low-level campaign as, the existence of a national agency that supports efforts for tobacco control policies with at least a minimal budget allocated. To classify as a medium-level campaign, the nation must meet the requirements for a low-level campaign and allocate annual tobacco control campaign expenditures (general) over the equivalent of $0.10 USD per capita. For a high-level campaign, the nation must have annual per capita expenditures over $0.25 USD per capita.

The campaigns with demonstrated effectiveness were those that involved a strong media component and grassroots organizations, such as those in California, Arizona, Massachusetts, Australia and Thailand ([7](#_ENREF_7)). In *Mexico* *SimSmoke*, the policy effect sizes used were 6.5% for a high-level, 3.25% for a medium level and 1.63% for a low-level campaign. These effects for tobacco control campaigns have been described previously in Levy et al. ([7](#_ENREF_7)). Te effects may be less for nondaily smokers, becauseinformation on risks may be assumed to be less relevant.

Prior to 2005, Mexico had only some educational programs in schools. There was a national media campaign in 2005, and a campaign prior to the implementation of smoke-free air laws in Mexico City ([79](#_ENREF_79)), a campaign in Guadalajara to accompany pictorial warning label implementation in 2010 ([80](#_ENREF_80)), and a national campaign in 2013 that was not evaluated. The 2009 MPOWER report indicated that Mexico had a national tobacco control agency with goals for 2008. It also reported that there were media campaigns and tobacco control expenditures totaling $200,000 MXN pesos (17,970 in USD) or $0.002 MXN peso per capita age 15+ (0.00013 USD) by 2008. The 2017 MPOWER reported that the overall national budget for tobacco control activities was $140,131,205 MXN pesos or $1.58 MXN peso per capita age 15+ (equivalent to $0.08 USD). Furthermore, the 2015 Global Adult Tobacco Survey (GATS) found that 32% of Mexican adults were aware of the "*Tabaco mata carita*" campaign – a mass media campaign directed to adolescent population, which aimed to disseminate the health consequences of smoking. Media campaigns have been sporadic, involve minimal expenditures and have low exposure rates, and tobacco control campaigns were increased from no campaign to a low level in 2005 ([81](#_ENREF_81), [82](#_ENREF_82)), and remained at that level.

### Marketing Bans

The marketing ban policy module in *SimSmoke* corresponds to the bans on advertising, promotion and sponsorship. Based on MPOWER recommendations, we categorized marketing bans into four levels: 1) no policy, 2) minimal policy, 3) moderate policy, and 4) complete policy. Overall enforcement of direct or indirect bans was measured from no enforcement (score of 0) to complete (score of 10) based on MPOWER reports. The effect sizes for marketing bans were based on a review by Levy et al.([7](#_ENREF_7)), relying primarily on the more comprehensive studies by Saffer and Chaloupka ([83](#_ENREF_83)) and Blecher ([84](#_ENREF_84)). Based on those studies (especially Blecher et al), a complete ban on direct and indirect marketing reduces the prevalence by 5%, increases the cessation rate by 4% and reduces the initiation rate by 8%. With a moderate ban (complete advertising and several indirect marketing bans), prevalence is reduced by 3%, cessation is increased by 2%, and initiation is reduced by 4%. With a low-level ban on advertising (some advertising), prevalence is reduced by 1%, the cessation rate is not affected, and the initiation rate is reduced by 1%. In our model, the impact of enforcement on marketing bans policy could be from being halved if enforcement is at a level of zero (no enforcement) to no impact on the effect size with a score of 10 (complete enforcement).

Based on information in Ramirez-Barba et al ([82](#_ENREF_82)), Mexico had minimal limits on advertising in 2002 and then only banned advertising on TV (except for some hours) and radio in 2004. By law in 2008, advertising was banned on all television, radio, and billboards and some types of sponsorship, starting in 2009; however, advertising was allowed in magazines that primarily targeted adults, in adult-only venues and adults-only points of sale, and through the mail ([85](#_ENREF_85)). In addition, promotional discounts, branding, and advertising at sponsored events were banned. Data from the 2009 GATS indicated that about half of Mexicans had seen advertisements in the last 30 days ([86](#_ENREF_86)). The survey *Encuesta Nacional de Consumo de Drogas, Alcohol y Tabaco* (ENCODAT) 2016 reported that 37.6% of the population had seen advertisements at points of sale. According to the ITC survey ([85](#_ENREF_85)), there was a reduction in the advertisements seen after 2008, especially in Mexico City. However, the policy implementation level in 2010 was still about 25% for television, 10% for radio, 20% for papers and magazines, 25% for posters and billboards, 10% for restaurants, and discos and bars, and about 5% each for sponsored sports and arts events. MPOWER reports indicated that the average compliance of direct and indirect marketing bans was 9 out of 10 points in 2007, 6 in 2008, 7 in 2010, 5 in 2012, 7 in 2014, and 4 in 2016. Mexico reported a 50% partial ban in 2002 and 2003, which increased to a 100% low level in 2004. This changed to a 75% moderate level and 25% low level for 2009-2018. Based on MPOWER data and Mexico’s evidence, we assumed a level of policy enforcement (compliance with bans) of 5 out of 10 for all the years.

### Health Warnings

The health warnings policy module in *SimSmoke* had four levels of policy implementation defined according to the percentage of display in the package and content of the warning. These levels included: no policy, low (< 30% of the principal display area of the pack), moderate (at least 30% of the principal display area of the pack and includes one or more of the seven pack warning criteria outlined in the Technical Note of Appendix II of the MPOWER Report), and high (at least 50% of the display area and includes all seven pack warning criteria, as well as a ban on deceitful terms). Strong health warnings reduced smoking prevalence by 4%, increased cessation by 10% and reduced initiation by 6%. When the level is set to moderate, prevalence is reduced by 2%, cessation is increased by 4%, and initiation is reduced by 2%. When the level is set to low, prevalence is reduced by 1%, cessation is increased by 2%, and initiation is reduced by 1%. Evidence on the effects of health warnings on cessation behaviors is provided in Levy et al. ([87](#_ENREF_87)).

ITC studies for Mexico have confirmed the effects of health warnings ([88-90](#_ENREF_88)). In addition, according to the 2016 ENCODAT, 85% of the smokers had noticed the health warnings on the cigarette packages, and 38% thought about quitting because of the label in the last month. However, nondaily smokers are likely to see health warnings less frequently than daily smokers, since nondaily smokers are more likely to buy single cigarettes and those who purchase by the pack, purchase less often. Consequently, we assumed that the effects of health warnings were reduced by 50% for nondaily smokers relative to smokers.

Based on information in Ramirez-Barba et al, health warnings in 2002 covered only a small portion of the pack are considered as low-level warnings. The warnings increased to 50% of the back of the pack in 2004 and changes to moderate-level. In 2011, they increased to 30% of the front and 100% of the back with graphic warnings, considering since then as a high-level policy. We categorized health warnings in Mexico as low for 2002-2003, moderate for 2004-2010, and high for 2011-2018.

### Cessation Treatment Policies

The cessation treatment policy module in *SimSmoke* is framed corresponding to the section “Cessation Programs: Treatment of Tobacco Dependence” in Appendix II of the 2008 MPOWER report. This module has four primary sub-policies: pharmacotherapy (PT) availability, cessation treatment financial coverage, quit lines and brief interventions ([91](#_ENREF_91), [92](#_ENREF_92))

*Pharmacotherapy availability*

The PT availability sub-policy option corresponded to the information in the MPOWER report regarding whether nicotine replacement treatments (NRT) and/or non-nicotine replacement therapies, such as Bupropion and Varenicline, were available and where they could be obtained. If Bupropion was available and NRT was available without a prescription, then prevalence is reduced by 1.0% in the first year of the policy (and maintained through future years), and the pre-policy cessation rate is increased by 6.0% per year in all future years following the first year of policy implementation. We assumed no effect on initiation.

Availability of NRT was categorized as 0 if not available or not reported, 1 if available in general stores and/or in pharmacies with a prescription, and 2 if available without a prescription. Availability of Bupropion was quantified only as 0 if not available or 1 if available. Since information about the availability of bupropion was not included in the MPOWER Reports, we assumed it was available once NRT was available. According to MPOWER Reports ([77](#_ENREF_77)), Mexico had NRT available in pharmacies without prescription since 2007. We assumed that same availability of NRT without prescription from 2002 to 2018.

#### Cessation treatment financial coverage

For the cessation treatment financial coverage sub-policy option, we followed the MPOWER Reports, which gives a score according to the level of coverage in each the following areas: primary care facilities, hospitals, offices of health professionals, community, and others. The possible scores were “fully (score 2)”, “partially (1)” or “none (0)”. We sum the scores to get the proportional effect with a maximum score of 10. The synergistic effect of the publicity on this financial coverage policy was captured by whether there also exists a high, medium or low level of tobacco control campaign (see above). With a high-level tobacco control campaign, this sub-policy would reduce the smoking prevalence by 2.25% in the first year of implementation (and maintained through future years) and increase the cessation rate by 12% annually in all future years. The aforementioned effects on prevalence and cessation were reduced by a 12.5% if a medium-level instead of high-level tobacco control campaign was implemented, and by 18.75% with a low-level campaign. Some recent evidence from Brazil ([93](#_ENREF_93)) and Great Britain ([94-98](#_ENREF_94)) provided results consistent with the above postulated effects.([99](#_ENREF_99))

Mexico has officially supported tobacco cessation, but pharmacotherapy for tobacco cessation was not included in the basic list of essential medications so that the patient is required to buy the drug in the pharmacy. From 2006 through 2012, a national program to support health care units for addictions treatment provided behavioral therapy for individuals and groups, but there were few referrals. According to 2009 GATS, 6% of those who made a quit attempt in the last year used pharmacotherapy and 3% used behavioral therapy. Only 3.5% of current smokers who made at least one quit attempt used pharmacotherapy according to 2016 ENCODAT. Reported use of pharmacotherapy based on ITC data was also low ([100](#_ENREF_100)). According to MPOWER reports, cessation supports have been partially covered in most primary care facilities, some hospitals, and communities since 2007 and have been available in some other places and offices since 2010.

#### Quit lines

The quit line sub-policy module was characterized, based on MPOWER reports, by whether or not the population has access to a toll-free quit line. Furthermore, *SimSmoke* categorizes quitlines as: passive =1, proactive (more than one call with attempts to encourage cessation treatment use) =2, or active with follow-up=3 ([91](#_ENREF_91), [92](#_ENREF_92)).

The effect size of quitlines depends also on publicity, using the same method as the one described above for the treatment financial coverage sub-policy. An active quitline with follow-up and high-level publicity reduced prevalence by 0.75% in the first year of the policy (and maintained through future years) and increased the cessation rate by 5.0% relative to its initial level in all future years. With medium level publicity, the effect on prevalence and cessation is reduced by 12.5%, and is reduced by 18.75% with low-level publicity. The rates for a passive quitline are one-third those of an active quitline with follow-up.

A quitline was been in place since 2008, but only provided referrals (considered a passive quitline) and promoted through pictorial warnings ([80](#_ENREF_80), [89](#_ENREF_89)).

#### Brief interventions

The brief interventions sup-policy module was ranked from 0 to 100%, where 100% described a more advanced form of interventions that involved screening for regular tobacco use, advice and follow-up on quitting and methods of quitting, training of the providers, charting, reminder systems, as well as integration with other services (quitlines, web-based cessation, and financial access). When fully implemented by itself, (a value of one), smoking prevalence is reduced by 1.0% in the first year (and maintained through future years), and the cessation rate is increased by 8.0%.

Data from the ITC survey ([100](#_ENREF_100)) indicated that 25% of smoker’s visited a physician in the last year, and of those about 37% received advice to quit smoking, 9% received a referral to a clinic, and about 9% received a pamphlet. The rates varied by city but stayed relatively constant over time. Data from 2009 GATS ([101](#_ENREF_101)) also indicated that brief interventions were provided to about 25% of smokers with little more than advice to quit. Data from 2016 ENCODAT indicated that 25% of smokers were advised to quit. We set the brief intervention index to a 20% level for all years.

*Combined policies*

If all of the sub-policies of the cessation treatment model were implemented at their highest level, smoking prevalence was reduced by 5.15% relative to no policies in the first year (and maintained through future years). The cessation rate was increased by 42.7% for the following years of implementation. Nondaily smokers may be less affected, due to lower perceived harms from smoking and being less likely to be advised by a doctor to quit.

### Youth Access Policies

Youth access policy includes those surrounding minimum purchase age laws, including enforcement efforts, and self-service and vending machine bans. A strongly enforced enforcement policy with bans reduces smoking prevalence in the first year and initiation rates in future years by 30% for youth below age 16 and by 20% for ages 16-17. Due to fewer purchases, nondaily smoking may be less affected.

Mexico has banned the sale of cigarettes to youth under the age of 18 years and sales from vending machines since 2002, but does not appear to enforce the law. According to the 2011 Global Youth Tobacco Surveys (GYTS) ([102](#_ENREF_102)), over 60% of youth were not refused the purchase of cigarettes at stores. According to 2016 ENCODAT, 75% of current adolescent smokers buy single cigarettes and 88% of the adolescent's currents smokers between 12-17 years report that they did not need ID to buy cigarettes. Based on the level of compliance from Global Youth Tobacco Surveys and ENCODAT surveys, youth access enforcement was considered at a low level with no vending machine or self-service bans since 2002. We also note that single cigarettes have been commonly sold to youth.

## Model Outcomes, Calibration, Validation, and Projection

As described above, *SimSmoke* estimates the effects over time for two primary outcomes: smoking prevalence and smoking-attributable deaths. Smoking prevalence is provided for the population ages 15 and above, but the model also has the capability to provide breakdowns by age. Separate results are provided by gender. *SimSmoke* estimates these outcomes for the tracking period, which is from 2002 to 2018, and projects future outcomes for 2019 through 2060.

The model using baseline prevalence without screening was first validated by comparing smoking rates predicted by *SimSmoke* to the 2011 ENA survey, the 2012 ENSANUT, 2009 and 2015 GATS survey and 2016 ENCODAT by the same measure. We also validated he model using baseline prevalence apply the 100 cigarette screen, and comparing the results with the non-screen data using the 2011 ENA, 2006 and 2012 ENSANUT, and 2016 ENCODAT by the same measure. These surveys were designed to provide national-level prevalence estimates ([41](#_ENREF_41), [42](#_ENREF_42), [50](#_ENREF_50)).

To examine the effect of policies, we first examine the effect of policies implemented between 2002 and 2018 on smoking rates and calculate the deaths averted resulting from those policies. We developed the counterfactual with no policies implemented by setting policies through 2018 to their 2002 levels to obtain the counterfactual with no policies. The difference between the actual (with policies) and counterfactual smoking rate yields the net effect of policies implemented since 2002. To gauge the effect of a single policy, we estimated the percentage reduction with only that policy relative to the counterfactual and compare that reduction to the summed effect of all policies. Deaths averted were estimated as the difference in smoking-attributable deaths with the policy or policies implemented and smoking-attributable deaths under the *counterfactual of no policies implemented.* We also estimated the impact of policies on smoking-attributable deaths through the year 2060 by subtracting the number of deaths with policies implemented from the number of deaths with policies kept at their 2002 levels. We considered policies individually and in combination.

After examining the effect of policies already implemented, we examined the potential effect of newly implemented future policies consistent with the FCTC treaty relative to the status quo, where tobacco control policies are maintained at their 2018 level. We then considered the effect of tobacco control policies in isolation and through a comprehensive tobacco control strategy, in each scenario implemented in 2019 and maintained through 2060 (a 40- year period).

In gauging comparing the effect of policies quo, we focus on the relative change in smoking prevalence, i.e., the change in smoking prevalence from the status quo to the future policy scenario divided by the status quo smoking prevalence. For smoking-attributable deaths, we consider deaths averted calculated as the difference between the number of smoking-related deaths under the new policy and the number of deaths under the status quo. For smoking-attributable deaths, we sum the deaths over the period 2018 through 2060 to obtain a cumulative estimate of the total excess deaths from current or former smokers.

Regarding the two measures of smoking (with and without 100-cigarettes screen) and two scenarios for the effect of policies on non-daily smokers (same as or half of daily smoking), four scenarios are simulated for prevalence validation and single policy evaluation, including: without the screen and same policy effect (Scenario 1), without the screen and half the policy effect (Scenario 2), with the screen and same policy effect (Scenario 3), and with the screen and half the policy effect (Scenario 4). The effects of stronger future policies are simulated only for the Scenario 1.

**RESULTS: SCENARIO 1**

**Validation of Current Daily and Nondaily Smoking Prevalence**

Figures 6.1 and 7.1 present the daily and nondaily smoking prevalence rates from *SimSmoke* and validation against Mexico surveys for scenario 1 with policy effects for nondaily smokers at the same level as for daily smokers and with the prevalence estimates without the screen.

For adults ages 15-65, *SimSmoke* predicts that current daily male smoking prevalence fell from 21.9% in 2002 to 15.4% in 2016, and female smoking prevalence fell from 7.1% to 4.6% over the same time period. Similarly, surveys show that between 2002 and 2016 male daily rates fell from 21.6% to 11.0% and female rates fell from 7.1% to 3.3%. Thus, from 2002 to 2016, SimSmoke predicted a relative decline in male daily smoking prevalence by 29.6 % compared to 49.1% from surveys, and female daily prevalence fell 34.8% compared to 53.5% from surveys. *SimSmoke* daily estimates were above the upper limits of the 95% confidence interval (CI) for survey estimates in 2009, 2011, 2015, and 2016, and were only within 95% CI in 2012 for both genders. By age group (see Table 2.1), the model overestimated male daily smoking rates in most years (especially for ages 25-44) and female daily smoking rates at the ages of 15-24 in all years. The predictions were below the 95% CI for males ages 15-24 (SimSmoke 10.3% vs ENSANUT 11.1%-13.6%) in 2012 and for females aged 25-44 (4.7% vs 5.5%-7.0%) and 45-65 (6.4% vs 6.9%-8.8%) in 2012. In terms of the relative change in 2002-2016 for all age groups, SimSmoke predicted the decreasing pattern from surveys for both males (SimSmoke -36.6% vs ENCODAT -54.3% for ages 15-24; -33.3% vs -53.5% for age 25-44; -17.9% vs -36.8% for age 45-65) and females (-33.9% vs -70.1% for ages 15-24; -38.6% vs -52.9% for age 25-44; -32.4% vs -41.8% for age 45-64).

For adult nondaily smokers, *SimSmoke* predicted that male prevalence fell from 12.3% in 2002 to 8.6% in 2016 and female prevalence fell from 4.4% to 3.2% over the same period. However, surveys show an increase from 12.3% in 2002 to 18.4% in 2016 for males and from 4.3% to 6.0% for females. Thus, from 2002 to 2016, SimSmoke predicted a relative decline in male nondaily smoking prevalence by 30.6% compared to a 49.6% relative increase from surveys, and female nondaily prevalence fell 26.8% compared to a 39.5% relative increase from surveys. *SimSmoke* predictions were below the confidence intervals of all survey estimates, except for the female rate in 2009 (SimSmoke 3.8% vs GATS 3.6%-5.3%). For the 18-24 and 25-64 age groups (see Table 2.1), *SimSmoke* predictions for male and female nondaily smoking were below survey 95% CIs between 2009 and 2016, except for females aged 25-44 in 2009. For ages of 45-65, the model did well for non-daily smokers but it underestimated the 2016 male and 2011 female prevalence. In terms of the relative change in 2002-2016 for ages 15-24 and 25-64, SimSmoke failed to predicted the increasing pattern from surveys for both males (SimSmoke -36.2% vs ENCODAT 66.9% for ages 15-24; -25.7% vs 75.4% for ages 25-44) and females (-34.1% vs 48.9% for ages 15-24; -35.3% vs 32.7% for ages 25-44). For ages of 45-65 in 2002-2016, the model failed to predicted the stable smoking rate for males (-32.8% vs 1.5%), but predicted the relative increase for females (31.4% vs 68.2%) although at less increase.

**The Effect of Tobacco Control Policies Implemented through 2018**

Tables 3.1 and 4.1 show the results for smoking prevalence and SADs comparing the status-quo scenario to the counterfactual of no additional policies implemented after 2002. The reductions due to individual or combined policies are shown relative to the counterfactual of the same year.

Male and female smoking prevalence were projected at 14.6% and 4.4% for daily and at 8.2% and 3.0% for nondaily smokers in the 2018 under the status quo with actual policies implemented. Compared to the counterfactual with no new policy implemented since 2002, *SimSmoke* projected that the 2018 male and female daily smoking prevalence were 25.6% and 26.7% lower and nondaily smoking prevalence were 26.9% and 27.3% lower than the prevalence in the counterfactual scenario by 2018. *SimSmoke* estimated a total (daily smokers) of 60,559 (53,393) male SADs and 18,181 (16,204) female SADs in 2018, increasing to 81,349 (71,220) male SADs and 20,207 (16,474) female SADs in 2060. Annual SADs for both genders combined were reduced by 8,220 in 2018, with a cumulative impact from 2002 to 2018 of 45,366 fewer SADs. By 2060, the impact of all policies for male and female daily smoking prevalence are projected to lead to a 34.7% and 38.6% reduction and the impact for male and female nondaily smoking to lead to a 38.2% and 38.6% reduction compared with the prevalence in the counterfactual scenario, which lead to 1,017,273 male and 273,228 female deaths averted by 2060.

Cigarette price increases by itself reduced the male and female daily smoking rates relatively by 14.4% and 15.5% and the nondaily smoking rates by 16.0% and 16.1% in 2018, and averted 644,571 total deaths by 2060. Smoke-free air laws yielded a 2.7% and 2.8% relative reduction for male and female daily smokers and 3.2% and 3.4% relative reduction for nondaily smokers in 2018, and averted 145,282 deaths. Health warnings reduced daily and nondaily smoking prevalence relatively by about 4.1% for both genders in 2018, and averted 231,142 deaths by 2060. Cessation treatments yielded about 2.1% relative reduction for male daily and non-daily smokers and 2.2% for females in 2018, and averted 165,316 deaths. The marketing bans yielded a 1.7% relative reduction for daily and non-daily smokers for both genders in 2018, and averted 81,387 deaths. Tobacco control campaigns yielded about 1.1% (1.2%) relative reduction for male (female) smokers in 2018, and averted 62,673 deaths. Of the reduction due to all policies for daily (non-daily) smokers, *SimSmoke* projects that 55%-57% (57%-58%) from increasing cigarettes prices, 15%-16% (14%-15%) from health warnings, 10%-11% (11%) from smoke-free air laws, 8% (7%-8%) from cessation treatments, 6% (6%) from marketing bans, and 4% (4%) from mass media campaigns.

**The Effects of Stronger Future Policies**

The effects of implementing stronger future policies were projected relative to trends with no policy changes from current levels in 2018, i.e., the status quo. Tables 5 and 6 respectively show the effects for smoking prevalence and SADs. Because the level of health warnings had reached its highest level by 2018 in Mexico, this policy shows no effects.

Among the available policy measures, increasing the cigarette tax is especially effective in reducing smoking prevalence, especially among youth ([4-7](#_ENREF_4)). We consider an increase in cigarette excise taxes from the current level of 53.5% to 60% and to 70% of the price. With the cigarette tax increased to 60%, rates for both daily and nondaily male (female) smokers were projected to decline by 3.7% (3.9%) initially at 2019 and by 7.1% (7.9%) at 2060. Further increasing the tax to 70% of the price, a short-term reduction in both daily and nondaily male (female) smoking prevalence of 10.9% (11.3%) is projected increasing to a 19.4% (21.2%) by 2060. The effects of taxes on deaths are delayed, because the effects of cessation on death rates are relatively slow to develop and because the greatest tax effects are on youth for whom health benefits occur at later ages. Increasing the cigarette tax to 60% of price is projected to lead to a cumulative total of 98,504 deaths averted by 2060 with the effects still growing. With the cigarette tax increased to 70% of price, cumulative deaths averted grow to 279,104 by 2060.

Comprehensive smoke-free air laws, with complete bans on smoking in all worksites, pubs and bars, restaurants and other public places throughout the country along with strong enforcement yielded a short-term (i.e., 2 years) 2.9% (2.9%) relative reduction in male (female) smoking prevalence, increasing to 3.8% (4.0%) by 2060 for both daily and nondaily smokers. This policy is projected to reduce SADs by 91,482 between 2019 and 2060.

We considered a well-funded tobacco control/media campaign directed at all smokers (i.e., not targeted to a specific population, such as youth) relative to the current policy of a low publicized campaign. *SimSmoke* predicts a short-term 5.6% (5.6%) reduction in male (female) smoking prevalence, increasing to 7.7% (8.0%) by 2060 with cumulative 186,113 fewer SADs. From implementing strong marketing restrictions with enforcement, a short-term 3.1% (3.1%) reduction in the male (female) smoking prevalence is projected, increasing to 5.8% (5.8%) by 2060 with 98,564 fewer SADs. For the smoking cessation treatment policy, we considered the combination of availability of nicotine replacement therapy and bupropion, the provision of quitlines, and the provision of cessation treatment. With the combined cessation policies at their highest level, *SimSmoke* predicts a short-term 1.9% (1.9%) reduction in the male (female) smoking prevalence, increasing to 4.7% (5.0%) by 2060 with a cumulative 150,594 fewer SADs. With strong enforcement of youth access laws and bans on vending machines and self-service, *SimSmoke* predicts a short-term 0.5% (0.6%) reduction in the male (female) smoking prevalence, increasing to 11.7% (15.5%) by 2060 with a cumulative 27,331 fewer SADs.

Research has shown that the most effective tobacco control approach is to adopt a comprehensive set of policy measures ([4-7](#_ENREF_4)), targeting different populations and filling different needs to reduce smoking prevalence and subsequent deaths. The final scenario projects the effect for a combination of policies, representing increasing the tax to 60% and 70%; comprehensive worksite and restaurant smoking bans with enforcement; a high intensity tobacco control/media campaign; a total ban on cigarette marketing with enforcement; strong health warnings; strong enforcement on youth access; and a comprehensive smoking cessation treatment program. With the cigarette tax increased to 60% of the price and all other policies implemented, daily and nondaily male (female) smoking prevalence are projected to decline by 13.6% (13.7%) in the short-term and by 31.0% (34.5%) by 2060 with 538,707 (393,718 males and 113,302 females) deaths averted by 2060. With a further increase of the cigarette tax to 70% of the price along with all other policies, smoking rates for males (females) are projected to fall by 22.9% (23.3%) initially and by 44.8% (48.7%) in 2060, with 761,929 (604,879 males and 157,050 females) deaths averted.

**RESULTS: SCENARIO 2**

**Validation of Current Daily and Nondaily Smoking Prevalence**

Figures 6.2 and 7.2 present the daily and nondaily smoking prevalence rates from *SimSmoke* and validation against Mexico surveys for scenario 2 with policy effects for nondaily smokers at half the level as for daily smokers and with the prevalence estimates without the screen.

For adults ages 15-65, *SimSmoke* predicts that current daily male smoking prevalence fell from 21.9% in 2002 to 15.3% in 2016, and female smoking prevalence fell from 7.1% to 4.6% over the same time period. Similarly, surveys show that between 2002 and 2016 male daily rates fell from 21.6% in 2002 to 11.0% in 2016 and females rates fell from 7.1 in 2002 and 3.3% in 2016. Thus, from 2002 to 2016, SimSmoke predicted a relative decline in male daily smoking prevalence by 29.9% compared to 49.1% from surveys, and female daily prevalence fell 34.9% compared to 53.5% from surveys. *SimSmoke* daily estimates were above the upper limit of the 95% confidence intervals for survey estimates in 2009, 2011, 2015, and 2016, and were only within 95% CI in 2012 for both genders. By age group (see Table 2.2), the model overestimated male daily smoking rates in most years (especially for ages 25-44) and female daily smoking rates at the ages of 15-24 in all years. The predictions were below the 95% CI for males aged 15-24 (SimSmoke 10.3% vs ENSANUT 11.1%-13.6%) in 2012 and for females aged 25-44 (4.7% vs 5.5%-7.0%) and 45-65 (6.4% vs 6.9%-8.8%) in 2012. In terms of the relative change in 2002-2016 for all age groups, SimSmoke predicted the decreasing pattern from surveys for both males (SimSmoke -37.2% vs ENCODAT -54.3% for ages 15-24; -33.5% vs -53.5% for age 25-44; -17.9% vs -36.8% for age 45-65) and females (-34.1% vs -70.1% for ages 15-24; -38.6% vs -52.9% for age 25-44; -32.4% vs -41.8% for age 45-64). The validation for daily smokers in scenario 2 shows similar pattern with the validation in scenario 1 (daily smokers and nondaily smokers share the same policy effects).

For adult nondaily smokers, *SimSmoke* predicted that male prevalence fell from 12.3% in 2002 to 10.0% in 2016 and female prevalence fell from 4.4% to 3.8% over the same period, while the surveys show an increase from 12.3% in 2002 to 18.4% in 2016 for males and from 4.3% to 6.0% in 2016 for females. Thus, from 2002 to 2016, SimSmoke predicted a relative decline in male nondaily smoking prevalence by 19.0 % compared to a 49.6% relative increase from surveys, and female nondaily prevalence fell 14.4% compared to a 39.5% relative increase from surveys. *SimSmoke* nondaily predictions were below the confidence intervals of all survey estimates, except for female prevalence in 2009 (SimSmoke 4.1% vs GATS 3.6%-5.3%). By age group, *SimSmoke* predictions for male and female nondaily smokers at ages of 15-24 and 25-44 were mostly below survey 95% CIs between 2009 and 2016, except for females in 2009. The model did well for female nondaily smokers ages of 45-65, but overestimated male nondaily smokers in 2009 and 2011 and underestimated the prevalence in 2016 for the 45-65 age groups. In terms of the relative change in 2002-2016 for ages 15-24 and 25-64, SimSmoke failed to predicted the increasing pattern from surveys for both males (SimSmoke -22.7% vs ENCODAT 66.9% for ages 15-24; -13.0% vs 75.4% for age 25-44) and females (-20.1% vs 48.9% for ages 15-24; -23.9% vs 32.7% for age 25-44). For ages of 45-65 in 2002-2016, the model failed to predicted the stable smoking rate for males (-24.8% vs 1.5%) but predicted the relative increase for females (47.6% vs 68.2%).

Compared with the scenario 1, reducing the policy effect sizes by 50% for nondaily smokers has higher nondaily smoking prevalence and better predictions for both genders. However, the new predictions are still lower than the estimates from various surveys. From the tables 2.1 and 2.2, we found that the survey data for females nondaily smokers generally show an increasing trend for ages 15-24, 25-44, 45-65, and 15-65 for both genders. Currently, the only increasing pattern estimated by the model is for female nondaily smokers at ages of 45-65 in both cases.

**The Effect of Tobacco Control Policies Implemented through 2018**

Tables 3.2 and 4.2 show the results for smoking prevalence and SADs comparing the status-quo scenario to the counterfactual of no additional policies implemented after 2002. The reductions due to individual or combined policies are shown relative to the counterfactual of the same year.

Male and female smoking prevalence were projected at 14.5% and 4.4% for daily and at 9.6% and 3.5% for nondaily smokers in the 2018 under the status quo with all policies implemented. Compared to the counterfactual with no new policy implemented since 2002, *SimSmoke* projected that the male and female daily smoking prevalence were 25.8% and 26.7% lower and nondaily smoking prevalence were 14.3% and 14.6% lower than the prevalence in counterfactual scenario by 2018. *SimSmoke* estimated a total (daily) smokers of 60,888 (53,385) male deaths and 18,270 (16,202) female SADs in 2018, increasing to 83,161 (70,702) male SADs and 21,069 (16,495) female SADs in 2060. Annual SADs for both genders combined were reduced by 7,804 in 2018, with a cumulative impact from 2002 to 2018 of 43,179 fewer SADs. By 2060, the impact of all policies for male and female daily smoking prevalence will lead to a 35.6% and 38.6% relative reduction and for male and female nondaily smoking to a 21.1% reduction compared with the prevalence in the counterfactual scenario, which lead to 966,143 male and 253,330 female deaths averted by 2060.

Cigarette price increases, by itself, reduced the male and female daily smoking rates relatively by 14.6% and 15.6% and the nondaily smoking rates by 8.3% and 8.4% in 2018, and it averted 611,376 total deaths by 2060. Smoke-free air laws yielded a 2.8% relative reduction for male and female daily smokers and 1.4% for nondaily smokers in 2018, and averted 136,480 deaths. Health warnings reduced daily and nondaily smoking prevalence relatively by about 4.1% and 2.0% for both genders in 2018, and averted 218,247 deaths by 2060. Cessation treatments yielded about 2.1% and 1.0% relative reduction for male daily and nondaily smokers and 2.2% and 1.1% for females in 2018, and averted 154,416 deaths. The marketing bans yielded a 1.7% and 0.8% relative reduction for daily and nondaily smokers for both genders in 2018, and averted 76,762 deaths. Tobacco control campaigns yielded about 1.2% and 0.6% relative reduction for daily and nondaily smokers (both genders) in 2018, and averted 58,963 deaths. Of the reduction due to all policies for daily (nondaily) smokers, *SimSmoke* projects that 55%-57% (58%-59%) from increasing cigarettes prices, 15%-16% (14%) from health warnings, 10%-11% (10%) from smoke-free air laws, 8% (7%-8%) from cessation treatments, 6% (6%) from marketing bans, and 4% (4%) from mass media campaigns.

**RESULTS: SCENARIO 3**

**Validation of Current Daily and Nondaily Smoking Prevalence**

Figures 6.3 and 7.3 present the daily and nondaily smoking prevalence rates from *SimSmoke* and validation against Mexico surveys in the third scenario with policy effects for nondaily smokers at the same level as for daily smokers and using the prevalence estimates with the screen.

For adults ages 15-65, *SimSmoke* predicts that current daily male smoking prevalence fell from 19.6% to 14.0% from 2002 to 2016, and female smoking prevalence fell from 6.1% to 4.2% over the same time period. Similarly, surveys show that between 2002 and 2016 male rates fell from 19.8% to 10.5% and female rates fell from 6.3% to 3.2%. Thus, from 2002 to 2016, SimSmoke predicted a relative decline in male daily smoking prevalence by 28.6% compared to 47.0% from surveys, and female daily prevalence fell 32.3% compared to 49.2% from surveys. Although the model and survey estimates have similar decreasing trends, *SimSmoke* daily estimates were below the 95% confidence intervals (CIs) for survey estimates in 2006 for males and 2012 for females, and were higher than the 95% CI in 2011 and 2016 for both genders. By age group (Table 2.3), the model underestimated male daily smokers at the ages of 15-24 and 45-65 in 2006 and 2012, but overestimated the male daily smokers at ages of 15-24 and 25-44 in 2011 and 2016 and ages 45-65 in 2016. The model underestimated female daily smokers at the ages of 25-44 in 2006 and 2012 and ages 45-65 in 2012, and overestimated daily smokers at ages of 15-24 in 2011, 2012, and 2016, and ages 25-44 in 2016. In terms of the relative change in 2002-2016 for all age groups, *SimSmoke* and the survey predicted the relative decreases for both males (*SimSmoke* -34.4% vs ENCODAT -49.2% for ages 15-24; -31.4% vs -51.4% for ages 25-44; -22.1% vs -37.0% for ages 45-65) and females (-34.0% vs -66.1% for ages 15-24; -31.7% vs -48.4% for ages 25-44; -34.8% vs -40.8% for ages 45-64).

For adult nondaily smokers, *SimSmoke* predicted that male prevalence fell from 6.8% in 2002 to 4.5% in 2016 and female prevalence fell from 1.6% to 1.2% over the same years. Surveys show an increase from 6.9% in 2002 to 12.3% in 2016 among males and 1.7% in 2002 to 3.6% in 2016. Thus, from 2002 to 2016, SimSmoke predicted a relative decline in male nondaily smoking prevalence by 33.9% compared to a 78.3% relative increase from surveys, and female nondaily prevalence fell 28.3% compared to a 111.8% relative increase from surveys. Nondaily *SimSmoke* predictions were below the confidence intervals of all survey estimates. By age group, *SimSmoke* predictions for male and female nondaily smoking rates were below survey 95% CIs in most years between 2006 and 2016. The model overestimated the smoking rate of males aged 45-65 in 2006. Two estimates (male smokers aged 45-65 in 2011 and female smokers aged 45-65 in 2006) from the model are within the 95% CI from various surveys. In terms of the relative changes by age group in 2002-2016, *SimSmoke* failed to predicted the increasing pattern from surveys for both males (SimSmoke -34.7% vs ENCODAT 208.3% for ages 15-24; -34.5% vs 90.9% for ages 25-44; -39.4% vs 5.1% for ages 45-65) and females (-33.6% vs 191.7% for ages 15-24; -34.3% vs 120.0% for ages 25-44; -14.9% vs 64.3% for ages 45-65).

**The Effect of Tobacco Control Policies Implemented through 2018**

Tables 3.2 and 4.3 show the results for smoking prevalence and SADs comparing the status-quo scenario to the counterfactual of no additional policies implemented after 2002. The reductions due to individual or combined policies are shown relative to the counterfactual of the same year.

Male and female smoking prevalence was projected at 13.4% and 4.0% for daily and at 4.5% and 1.1% for nondaily in the 2018 under the status quo with all policies implemented. Compared to the counterfactual with no new policy implemented since 2002, *SimSmoke* projected that the male and female daily smoking prevalence were 25.8% and 26.6% lower and nondaily smoking prevalence were 25.1% and 26.5% lower than the prevalence in counterfactual scenario in 2018. *SimSmoke* estimated a total of 56,512 (51,008 for daily smokers) male SADs and 15,784 (14,536) female SADs in 2018, increasing to 74,512 (68,245) male SADs and 17,626 (16,099) female SADs in 2060. Annual SADs for both genders combined were reduced by 7,620 in 2018, with a cumulative impact from 2002 to 2018 of 42,553 fewer SADs. By 2060, the impact of all policies for male and female daily smoking prevalence will lead to a 35.4% and 37.6% reduction and the impact for male and female nondaily smoking will lead to a 34.6% and 37.3% reduction compared with the prevalence in the counterfactual scenario, which lead to 931,085 male and 232,611 female deaths averted by 2060.

Cigarette price increases by itself reduced the male and female daily smoking rates relatively by 14.5% and 15.3% and the nondaily smoking rates by 13.6% and 14.8% in 2018, and averted 579,920 total deaths by 2060. Health warnings reduced daily smoking prevalence relatively by 4.2% and 4.1% for males and females and reduced nondaily smoking prevalence relatively by 4.1% and 4.3% for males and females in 2018, and it averted 212,875 deaths by 2060. Smoke-free air laws yielded a 2.8% relative reduction for male and female daily smokers and nondaily smokers in 2018, and averted 131,692 deaths by 2060. Cessation treatments yielded about 2.0% and 2.1% relative reduction for male and female daily smokers and 2.2% and 2.3% relative reduction for male and female nondaily smokers in 2018, and it averted 144,766 deaths by 2060. The marketing bans yielded about 1.7% relative reduction for daily and nondaily smokers for both genders in 2018, and averted 75,158 deaths by 2060. Tobacco control campaigns yielded about 1.2% relative reduction for daily and nondaily smokers for both genders in 2018, and averted 56,923 deaths by 2060. Of the reduction due to all policies for daily (nondaily) smokers, *SimSmoke* projects that 55%-56% (53%-54%) from increasing cigarettes prices, 15%-16% (16%) from health warnings, 10%-11% (10%-11%) from smoke-free air laws, 8% (9%) from cessation treatments, 6% (6%) from marketing bans, and 4% (4%-5%) from mass media campaigns.

**RESULTS: SCENARIO 4**

**Validation of Current Daily and Nondaily Smoking Prevalence**

Figures 6.4 and 7.4 present the daily and nondaily smoking prevalence rates from *SimSmoke* and validation against Mexico surveys in the fourth scenarios with policy effects for nondaily smokers at half the level as for daily smokers and using the prevalence estimates with the screen.

For adults ages 15-65, *SimSmoke* predicts that current daily male smoking prevalence fell from 19.6% to 14.0% from 2002 to 2016, and female smoking prevalence fell from 6.1% to 4.2% over the same time period. Similarly, surveys indicate that between 2002 and 2016 male rates fell from 19.8% to 10.5% and female rates fell from 6.3% to 3.2%. Thus, from 2002 to 2016, SimSmoke predicted a decline in male daily smoking prevalence by 28.7% compared to 47.0% from surveys, and female daily prevalence fell 32.3% compared to 49.2% from surveys. Although the model and survey estimates have similar decreasing trends, *SimSmoke* daily estimates were below the 95% confidence intervals (CIs) for survey estimates in 2006 for males and 2012 for females, and were higher than the 95% CI in 2011 and 2016 for both genders. By age group (Table 2.4), the model underestimated male daily smokers at the ages of 15-24 and 45-65 in 2006 and 2012, but overestimated male daily smokers ages 15-24 and ages 25-44 in 2011 and 2016 and ages 45-65 in 2016. The model underestimated female daily smokers ages of 25-44 in both 2006 and 2012 and ages 45-65 in 2012, and overestimated smokers ages 15-24 in 2011, 2012, and 2016, and ages 25-44 in 2016. In terms of the relative change in 2002-2016 for all age groups, *SimSmoke* and the survey predicted the relative decreases for both males (*SimSmoke* -34.5% vs ENCODAT -49.2% for ages 15-24; -31.5% vs -51.4% for ages 25-44; -22.1% vs -37.0% for ages 45-65) and females (-34.0% vs -66.1% for ages 15-24; -31.7% vs -48.4% for ages 25-44; -34.8% vs -40.8% for ages 45-64). The validation for daily smokers in the third and fourth scenarios are similar to each other, since the change in policy effect size is on nondaily smokers.

For adult nondaily smokers, *SimSmoke* predicted that male prevalence fell from 6.8% in 2002 to 5.2% in 2016, and female prevalence fell from 1.6% in 2002 to 1.4% in 2016. Surveys show an increase from 6.9% to 12.3% for males and 1.7% to 3.6% for females during 2002-2016. Thus, from 2002 to 2016, SimSmoke predicted a relative decline in male nondaily smoking prevalence by 23.6% compared to 78.3% relative increase from surveys, and female nondaily prevalence fell 16.6% compared to a 111.8% relative increase from surveys. Nondaily *SimSmoke* predictions were below the confidence intervals of all survey estimates. By age group, *SimSmoke* predictions for male and female nondaily smoking rates were below survey 95% CIs in most years between 2006 and 2016. The model overestimated the smoking rate of males aged 45-65 in 2006. Two estimates (male smokers aged 45-65 in 2011 and female smokers aged 45-65 in 2006) from the model are within the 95% CI from various surveys. In terms of the relative changes by age group in 2002-2016, *SimSmoke* failed to predicted the increasing pattern from surveys for both males (SimSmoke -20.9% vs ENCODAT 208.3% for ages 15-24; -23.6% vs 90.9% for ages 25-44; -32.2% vs 5.1% for ages 45-65) and females (-19.6% vs 191.7% for ages 15-24; -22.9% vs 120.0% for ages 25-44; -4.5% vs 64.3% for ages 45-65).

**The Effect of Tobacco Control Policies Implemented through 2018**

Tables 3.4 and 4.4 show the results for smoking prevalence and SADs comparing the status-quo scenario to the counterfactual of no additional policies implemented after 2002. The reductions due to individual or combined policies are shown relative to the counterfactual of the same year.

Male and female smoking prevalence were projected at 13.4% and 4.0% for daily and at 5.2% and 1.3% for nondaily in the 2018 under the status quo with all policies implemented. Compared to the counterfactual with no new policy implemented since 2002, *SimSmoke* projected that the male and female daily smoking prevalence were 25.9% and 26.6% lower and nondaily smoking prevalence were 13.0% and 14.1% lower than the prevalence in counterfactual scenario in 2018. *SimSmoke* estimated a total of 56,761 (51,000 for daily smokers) male SADs and 15,838 (14,535) female SADs in 2018, increasing to 74,686 (68,082) male SADs and 17,957 (16,102) female SADs in 2060. Annual SADs for both genders combined were reduced by 7,316 in 2018, with a cumulative impact from 2002 to 2018 of 40,894 fewer SADs. By 2060, the impact of all policies for male and female daily smoking prevalence will lead to a 35.6% and 37.6% reduction and the impact for male and female nondaily smoking will lead to a 17.9% and 20.3% reduction compared with the prevalence in the counterfactual scenario, which lead to 896,313 male and 224,102 female deaths averted by 2060.

Cigarette price increases by itself reduced the male and female daily smoking rates relatively by 14.6% and 15.4% and the nondaily smoking rates by 6.8% and 7.7% in 2018, and averted 559,765 total deaths by 2060. Health warnings reduced daily smoking prevalence relatively by 4.2% and 4.1% for males and females and reduced nondaily smoking prevalence relatively by 2.0% and 2.1% for males and females in 2018, and averted 204,758 deaths by 2060. Smoke-free air laws yielded a 2.8% relative reduction for daily smokers and 1.4% for nondaily smokers for both genders in 2018, and averted 126,271 deaths by 2060. Cessation treatments yielded about 2.1% relative reduction for male and female daily smokers and 1.1% and 1.2% relative reduction for male and female nondaily smokers in 2018, and averted 138,605 deaths by 2060. The marketing bans yielded about 1.7% and 0.8% relative reduction for daily and nondaily smokers for both genders in 2018, and averted 72,168 deaths by 2060. Tobacco control campaigns yielded about 1.2% and 0.6% relative reduction for daily and nondaily smokers for both genders in 2018, and averted 54,584 deaths by 2060. Of the reduction due to all policies for daily (nondaily) smokers, *SimSmoke* projects that 55%-56% (54%-55%) from increasing cigarettes prices, 15%-16% (16%) from health warnings, 10%-11% (10%-11%) from smoke-free air laws, 8% (8%-9%) from cessation treatments, 6% (6%) from marketing bans, and 4% (4%) from mass media campaigns.

**DISCUSSION**

Unlike previous *SimSmoke* models ([23-38](#_ENREF_23)), including an earlier model for Mexico ([40](#_ENREF_40)), the revised *Mexico* *SimSmoke* did not validate well. Different from the earlier Mexico model, we separately modeled daily and nondaily smoking. Between 2002 and 2016, *SimSmoke* predicted a decline in nondaily smoking rates for males and females, while survey rates instead showed a strong increasing trend. The increase in nondaily smoking occurred primarily among males ages 15-44, but was also observed for females. *SimSmoke* also under-predicted the reduction in male daily smoking, again largely among the younger population. This finding suggests male smokers were changing from daily to nondaily smoking due to some unexplained pattern (e.g., not policy related to current specification of policies).

The reductions in both daily and nondaily smoking predicted by *SimSmoke* were largely due to policy changes over the time period 2002-2018. Cigarettes excise tax policy accounted for about 60% of the reduction due to policies, followed by smoke-free air laws, health warnings, cessation treatment policies, and tobacco control campaigns. We also projected the future effect of implementing stricter policies than those already in place. Increasing the cigarette excise tax to 60% of price yielded a 7.5% long-term relative reduction in smoking prevalence, while increasing the tax to 70% yielded a 20% prevalence reduction. Increased tobacco control spending was projected to reduce long-term prevalence by 8%, while cessation treatments yielded a 5% reduction. Mostly through better enforcement, smoking prevalence was projected to decline by 4% from widespread smoke-free air laws and 6% from comprehensive marketing restrictions. In evaluating these policy effects, but the model over-predicted the reduction in overall smoking prevalence, especially for nondaily smokers, so that the effects are likely over-stated. Nevertheless, the model suggests that higher taxes, better enforcement of smoke-free air laws and marketing restrictions, media campaigns and cessation treatment policies can play a major role in reducing future rates of smoking.

The *SimSmoke* policy effect sizes for daily smokers and nondaily smokers were assumed the same, except for health warnings, which were considered to have less effect on nondaily smokers due to packages being observed less frequently. We did not consider variations in effect size for other policies. While daily smokers may be less addicted and therefor more easily influenced by tobacco control policies, price policies may also have less effect on nondaily smokers if, in reaction to higher costs, smokers may instead switch from daily to nondaily smoking. For example, daily smokers may switch to smoking nondaily and/or nondaily smokers are more likely to reduce the number of cigarettes smoked rather than quit. In particular, the effect of price may be influenced by tendencies of Mexican smokers to smoke single cigarettes ([52](#_ENREF_52), [103](#_ENREF_103)). Based on the 2016 ENCODAT ([42](#_ENREF_42)), 62.1% of the nondaily smokers and 25.9% of the daily smokers buy single cigarettes. Those who bought single cigarettes paid more than double per cigarette compared to those who bought packages. For this reason, the elasticities are less clear, since they may be less responsive to the higher prices actually paid by those who buy single cigarettes.

Other policies may also have less influence on nondaily than daily smokers. Smoke-free air laws may also have less impact on nondaily smokers, if, for example, they are less affected by not being able to smoke at work or in public places. While cessation treatment policies could be more effective for nondaily than daily smokers, because they are less dependent, cessation treatment policies may instead have less effect on nondaily smokers, because doctors are less likely to advise nondaily smokers to quit or nondaily smokers see less reason to quit than daily smokers. Media campaigns may play less of a role with nondaily smokers, if they do not consider themselves regular smokers or if they view their health risks to be substantially lower than for smokers. However, both media campaigns and cessation treatment policies can play a more important role with nondaily smoker. For example, media campaigns may better communicate the health risks to nondaily smokers, even at low levels of smoking, and encourage cessation treatments by health providers for nondaily smokers and design treatments to meet the needs of nondaily smokers. Further research is needed on whether policies differentially affect nondaily compared to daily smokers, and how policies may be tailored to nondaily smoking.

Another limitation of the model is the failure to consider changes in smoking patterns, which arose from changes in the supply of tobacco products. One such change is the industry provision of cigarettes with flavor capsules, which are particularly appealing to young smokers ([104](#_ENREF_104), [105](#_ENREF_105)). We also did not consider the role of single cigarettes, i.e., “loosies”, which may also play an important role in smoking patterns of youth ([52](#_ENREF_52), [103](#_ENREF_103)). Limits could be placed on both of these practices. In addition, we did not consider smuggling. Although a relatively recent occurrence and now banned, the appearance of e-cigarettes onto the market may have also played a role in recent smoking patterns ([106-111](#_ENREF_106)).

Finally, better measures of prevalence, especially for nondaily use, are needed. We used estimates where the screening question of having smoked 100 cigarettes in one’s lifetime was not applied. Using the 2002, 2011 and 2016 ENA and 2012 ENSANUT surveys for Mexico, we found that the relative difference in prevalence estimates with and without the screen was greater for nondaily than daily smoking prevalence, although the screen also had a minor effect even on daily smoking prevalence. The variation was especially large among nondaily smokers aged 15-24, with as much as a 70% difference in prevalence with and without the screen. Similar results, although less prominent, were obtained in a US study ([112](#_ENREF_112)).

In summary, the results from the *Mexico SimSmoke* indicate a need to reformulate the model to better capture the evolution of nondaily smoking and to better understand the relationship of nondaily to daily smoking. Nevertheless, in the failure to capture these trends, *SimSmoke* helps to identify gaps in surveillance and points to the need for evaluations of tobacco control policies that can effectively reduce nondaily smoking.

**List of fifteen tables:**

Table 2.1-4 Validation of daily and nondaily smoking prevalence, ages 15-65 by gender, from Mexico SimSmoke and various national surveys*, 2002-2016 (for Scenario 1-4)

Table 3.1-4 Prevalence by smoking status and gender, from Mexico *SimSmoke* under various policy scenarios in 2002-2060 (for Scenario 1-4)

Table 4.1-4 Smoking-attributable deaths (SADs) and lives saved† by smoking status and gender, from Mexico *SimSmoke* under various policy scenarios in 2002-2060 (for Scenario 1-4)

Table 5. Prevalence by smoking status and gender, from Mexico *SimSmoke* under various stronger future policies, 2002-2060 for Scenario 1

Table 6. Smoking-attributable deaths (SADs) and lives could be saved by smoking status and gender, from Mexico SimSmoke under various stronger future policies in 2002-2060 for Scenario 1

**List of eight figures (not including Fig 1.for mortality, Fig 2-3 for immigration, Fig 4 for population in the Report):**

Figure 6.1-4 Validation of Daily Smoking Prevalence, Ages 15-65 by gender, from *Mexico* *SimSmoke* and Various National Surveys*, 2002-2016 (for Scenario 1-4)

Figure 7.1-4 Validation of Nondaily Smoking Prevalence, Ages 15-65 by gender, from *Mexico* *SimSmoke* and Various National Surveys*, 2002-2016 (for Scenario 1-4)

**Table 1. Tobacco Control Policies, Specifications and Policy Effect Sizes***

| **Policy** | **Description** | **Policy Effect Size**** |
| --- | --- | --- |
| **Cigarette Excise Taxes** | | |
| **Cigarette price/tax** | The effect of taxes is directly incorporated through the average price after tax. The price elasticity is used to convert the % price changes into effect sizes. | Elasticities  -0.4 for ages 14-17  -0.3 for ages 18-24  -0.2 for ages 25-34  -0.1 for ages 35-64  -0.2 for ages 65+ |
| **Smoke-Free Air Laws** | | |
| **Worksite smoking ban** | Ban in all indoor worksites, with strong enforcement of laws (reduced by 1/3 if allowed in ventilated areas and by 2/3 if allowed in common areas) | -6% |
| **Restaurant smoking ban** | Ban in all indoor restaurants (scaled for lower coverage), with strong enforcement of laws | -2% |
| **Pubs and bars smoking ban** | Ban in all indoor in pubs and bars (scaled for lower coverage), with strong enforcement of laws | -1% |
| **Other place bans** | Ban in 3 out of 4 government buildings (scaled for lower coverage), retail stores, public transportation, and elevators , with strong enforcement of laws | -1% |
| **Enforcement and Publicity** | Government agency enforces the laws and publicity via tobacco control campaigns | Effects reduced 50% absent publicity and enforcement |
| **Tobacco Control Campaigns** | | |
| **High publicity media campaign** | Campaign publicized heavily on TV and at least some other media, with a social marketing approach | -6.5% |
| **Medium publicity media campaign** | Campaign publicized sporadically on TV and at least some other media | -3.25% |
| **Low publicity media campaign** | Campaign publicized only sporadically in newspaper, billboard, or some other media | -1.63% |
| **Marketing Restrictions** | | |
| **Comprehensive marketing ban** | Ban is applied to television, radio, print, billboard, in-store displays, sponsorships and free samples (all indirect marketing) | -5% prevalence,  -8% initiation,  +4% cessation |
| **Moderate advertising ban** | Ban is applied to all media (television, radio, print, billboard) plus one indirect marketing medium | -3% prevalence,  -4% initiation,  +2% cessation |
| **Minimal advertising ban** | Ban is applied to some television, radio, print, and billboard | -1% prevalence and -1% initiation only |
| **Enforcement** | Government agency enforces the laws | Effects reduced 50% absent enforcement |

| **Health Warnings** | | |
| --- | --- | --- |
| **High** | Labels are large, bold and graphic, and cover at least 50% of pack | -4% prevalence,  -6% initiation,  +10% cessation |
| **Moderate** | Laws cover at least 30% of package, not bold or graphic | -2% prevalence,  -2% initiation,  +4% cessation |
| **Low** | Laws cover less than 30 of package, not bold or graphic | -1% prevalence,  -1% initiation,  +2% cessation |
| **Cessation Treatment Policies** | | |
| **Availability of pharmacotherapies** | Legality of nicotine replacement therapy and/or Bupropion and Varenecline | -1% prevalence,  +6% cessation |
| **Cessation treatment financial coverage** | Payments to cover pharmacotherapy and behavioral cessation treatment with high publicity (Effect size reduced by 12.5% with moderate publicity and 18.75% with low publicity) | -2.25% prevalence,  +12% cessation |
| **Quitline** | Three quit line types: passive, proactive and active with follow-up. (Effect size reduced by 1/3 if quit line is proactive, reduced by 2/3 if quit line passive). | -0.75% prevalence,  +7.5% cessation |
| **Brief interventions** | Advice by health care provider to quit and methods provided | -1% prevalence,  +8% cessation |
| **All cessation policies combined** | Complete availability and reimbursement of pharmaco- and behavioral treatments, quit lines, and brief interventions | -5.15% prevalence, +42.7% cessation |
| **Youth Access Policies** | | |
| **Strong enforcement & well publicized** | Compliance checks are conducted 4 times per year per outlet, penalties are potent and enforced with heavy publicity | -16% initiation and prevalence for ages 16-17 and -24% ages 14-15 |
| **Moderate enforcement with some publicity** | Compliance checks are conducted regularly, penalties are potent, and publicity and merchant training are included | -8% initiation and prevalence for ages 16-17 and -12% ages 14-15 |
| **Low enforcement** | Compliance checks are conducted sporadically, penalties are weak | -2% initiation and prevalence for ages 16-17 and -3% ages 14-15 |
| **Vending machine restrictions** | Total ban, with strong enforcement | 8% |
| **Self-service restrictions** | Total ban, with strong enforcement | 4% |

* References can be found in the text.

** Policy effect sizes are the assumed to be the same for prevalence rates (first year) and initiation rates (after the first year) and to be the opposite effect for cessation rates (after the first year) unless otherwise stated.

|  | | | **Male** | | | | | | **Relative difference** | |
| --- | --- | --- | --- | --- | --- | --- | --- | --- | --- | --- |
| **Daily** | **Age groups** | | **2002** | **2009** | **2011** | **2012** | **2015** | **2016** | **2002-2011** | **2002-2016** |
|  | 15-65 | SimSmoke | 21.9% | 18.4% | 16.8% | 16.5% | 15.6% | 15.4% | -23.4% | -29.6% |
|  |  | Surveys | 21.6% | 12.0% | 11.6% | 16.0% | 12.1% | 11.0% | -46.3% | -49.1% |
|  |  | 95% CI | 19.6%,23.8% | 10.7%,13.4% | 10.4%,13.0% | 15.2%,16.9% | 10.8%,13.6% | 10.2%,11.9% |  |  |
|  | 15-24 | SimSmoke | 15.8% | 11.7% | 10.4% | 10.3% | 10.0% | 10.0% | -34.0% | -36.6% |
|  |  | Surveys | 15.1% | 10.9% | 6.6% | 12.3% | 10.4% | 6.9% | -56.3% | -54.3% |
|  |  | 95% CI | 12.0%,18.7% | 8.4%,14.1% | 5.2%,8.3% | 11.1%,13.6% | 8.2%,13.1% | 5.8%,8.2% |  |  |
|  | 25-44 | SimSmoke | 26.6% | 22.0% | 19.8% | 19.4% | 18.0% | 17.7% | -25.4% | -33.3% |
|  |  | Surveys | 26.9% | 11.5% | 13.0% | 19.2% | 12.8% | 12.5% | -51.7% | -53.5% |
|  |  | 95% CI | 23.7%,30.3% | 9.7%,13.6% | 11.2%,15.0% | 18.0%,20.5% | 10.8%,15.1% | 11.0%,14.1% |  |  |
|  | 45-65 | SimSmoke | 20.8% | 19.8% | 18.5% | 18.3% | 17.4% | 17.1% | -11.1% | -17.9% |
|  |  | Surveys | 20.9% | 14.2% | 15.4% | 18.8% | 12.9% | 13.2% | -26.3% | -36.8% |
|  |  | 95% CI | 17.1%,25.2% | 12.1%,16.6% | 12.6%,18.6% | 17.3%,20.4% | 10.6%,15.5% | 11.8%,14.6% |  |  |
| **Non-daily** | **Age groups** | | **2002** | **2009** | **2011** | **2012** | **2015** | **2016** | **2002-2011** | **2002-2016** |
|  | 15-65 | SimSmoke | 12.3% | 10.4% | 9.4% | 9.3% | 8.7% | 8.6% | -23.7% | -30.6% |
|  |  | Surveys | 12.3% | 13.8% | 15.6% | 15.7% | 14.1% | 18.4% | 26.8% | 49.6% |
|  |  | 95% CI | 10.9%,13.9% | 12.6%,15.2% | 14.2%,17.0% | 14.9%,16.5% | 12.8%,15.5% | 17.4%,19.5% |  |  |
|  | 15-24 | SimSmoke | 12.6% | 9.6% | 8.5% | 8.3% | 8.0% | 8.0% | -32.7% | -36.2% |
|  |  | Surveys | 12.4% | 15.8% | 19.0% | 15.9% | 17.1% | 20.7% | 53.2% | 66.9% |
|  |  | 95% CI | 9.6%,15.9% | 13.5%,18.4% | 16.3%,21.9% | 14.6%,17.4% | 14.4%,20.1% | 18.9%,22.7% |  |  |
|  | 25-44 | SimSmoke | 11.9% | 10.4% | 9.5% | 9.4% | 8.9% | 8.9% | -20.0% | -25.7% |
|  |  | Surveys | 11.8% | 15.1% | 17.4% | 18.9% | 15.4% | 20.7% | 47.5% | 75.4% |
|  |  | 95% CI | 9.6%,14.5% | 13.4%,16.9% | 15.2%,19.7% | 17.8%,20.1% | 13.5%,17.5% | 19.1%,22.3% |  |  |
|  | 45-65 | SimSmoke | 12.9% | 11.3% | 10.3% | 10.0% | 9.0% | 8.6% | -20.1% | -32.8% |
|  |  | Surveys | 13.0% | 8.9% | 8.7% | 10.4% | 9.0% | 13.2% | -33.1% | 1.5% |
|  |  | 95% CI | 9.7%,17.1% | 7.0%,11.4% | 7.1%,10.7% | 9.3%,11.5% | 7.3%,11.1% | 11.6%,15.0% |  |  |

**Table 2.1 Validation of daily and nondaily smoking prevalence, ages 15-65 by gender, from Mexico *SimSmoke* and various national surveys*, 2002-2016. Without 100-cigarettes screen and full policies effect.**

|  | | | **Female** | | | | | | **Relative difference** | |
| --- | --- | --- | --- | --- | --- | --- | --- | --- | --- | --- |
| **Daily** | **Age groups** | | **2002** | **2009** | **2011** | **2012** | **2015** | **2016** | **2002-2011** | **2002-2016** |
|  | 15-65 | SimSmoke | 7.1% | 5.9% | 5.2% | 5.1% | 4.7% | 4.6% | -26.4% | -34.8% |
|  |  | Surveys | 7.1% | 3.8% | 3.8% | 5.4% | 3.9% | 3.3% | -46.5% | -53.5% |
|  |  | 95% CI | 5.9%,8.6% | 3.0%,4.8% | 3.3%,4.5% | 4.9%,5.8% | 3.2%,4.7% | 3.0%,3.7% |  |  |
|  | 15-24 | SimSmoke | 6.6% | 5.2% | 4.6% | 4.6% | 4.4% | 4.4% | -30.2% | -33.9% |
|  |  | Surveys | 6.7% | 2.4% | 2.2% | 3.1% | 2.4% | 2.0% | -67.2% | -70.1% |
|  |  | 95% CI | 4.7%,9.6% | 1.5%,3.6% | 1.4%,3.4% | 2.5%,3.8% | 1.5%,3.7% | 1.5%,2.7% |  |  |
|  | 25-44 | SimSmoke | 7.1% | 5.5% | 4.8% | 4.7% | 4.4% | 4.3% | -31.5% | -38.6% |
|  |  | Surveys | 7.0% | 3.7% | 4.3% | 6.2% | 3.8% | 3.3% | -38.6% | -52.9% |
|  |  | 95% CI | 5.6%,8.8% | 2.7%,4.9% | 3.5%,5.2% | 5.5%,7.0% | 3.0%,4.9% | 2.8%,3.9% |  |  |
|  | 45-65 | SimSmoke | 7.9% | 7.2% | 6.6% | 6.4% | 5.6% | 5.3% | -16.6% | -32.4% |
|  |  | Surveys | 7.9% | 5.9% | 4.9% | 7.8% | 5.2% | 4.6% | -38.0% | -41.8% |
|  |  | 95% CI | 6.0%,10.2% | 4.5%,7.5% | 3.7%,6.4% | 6.9%,8.8% | 3.5%,7.6% | 4.0%,5.3% |  |  |
| **Non-daily** | **Age groups** | | **2002** | **2009** | **2011** | **2012** | **2015** | **2016** | **2002-2011** | **2002-2016** |
|  | 15-65 | SimSmoke | 4.4% | 3.8% | 3.5% | 3.4% | 3.2% | 3.2% | -21.1% | -26.8% |
|  |  | Surveys | 4.3% | 4.4% | 6.1% | 5.5% | 4.9% | 6.0% | 41.9% | 39.5% |
|  |  | 95% CI | 3.4%,5.5% | 3.6%,5.3% | 5.3%,7.0% | 5.1%,6.0% | 4.2%,5.8% | 5.5%,6.6% |  |  |
|  | 15-24 | SimSmoke | 4.7% | 3.6% | 3.2% | 3.2% | 3.1% | 3.1% | -30.7% | -34.1% |
|  |  | Surveys | 4.7% | 5.1% | 8.2% | 6.2% | 5.1% | 7.0% | 74.5% | 48.9% |
|  |  | 95% CI | 3.2%,6.8% | 3.7%,6.9% | 6.3%,10.6% | 5.4%,7.0% | 3.6%,7.0% | 6.0%,8.2% |  |  |
|  | 25-44 | SimSmoke | 5.2% | 4.2% | 3.7% | 3.6% | 3.4% | 3.4% | -28.5% | -35.3% |
|  |  | Surveys | 5.2% | 4.7% | 5.6% | 5.8% | 6.2% | 6.9% | 7.7% | 32.7% |
|  |  | 95% CI | 3.8%,7.2% | 3.7%,5.9% | 4.6%,6.8% | 5.2%,6.6% | 5.0%,7.6% | 6.1%,7.8% |  |  |
|  | 45-65 | SimSmoke | 2.4% | 3.3% | 3.3% | 3.3% | 3.1% | 3.1% | 37.7% | 31.4% |
|  |  | Surveys | 2.2% | 2.9% | 4.8% | 3.9% | 2.8% | 3.7% | 118.2% | 68.2% |
|  |  | 95% CI | 1.4%,3.3% | 1.9%,4.3% | 3.6%,6.3% | 3.3%,4.7% | 2.0%,4.0% | 3.1%,4.5% |  |  |

*The survey point estimates (indicated by dots) and 95% confidence intervals were reported from various surveys, including the Encuesta Nacional de Adicciones (ENA) in 2002 and 2011, the Global Adult Tobacco Survey (GATS) in 2009 and 2015, the Encuesta Nacional de Salud y Nutricion (ENSANUT) in 2012, and the Encuesta Nacional de Consumo de Drogas, Alcohol y Tabaco (ENCODAT) in 2016.

**Table 3.1 Prevalence by smoking status and gender, from Mexico *SimSmoke* under various scenarios in 2002-2060. Scenario 1, without 100-cigarettes screen and full policies effect.**

| **Male** | | | | | | | |
| --- | --- | --- | --- | --- | --- | --- | --- |
| **Scenarios** | **Type of smoker** | **2002** | **2018** | **2019** | **2060** | **Relative difference** | |
|  |  |  |  |  |  | **in 2018** | **in 2060** |
| **Counterfactual*** | **Nondaily** | 11.9% | 11.3% | 10.1% | 9.5% | - | - |
|  | **Daily** | 21.3% | 19.6% | 19.5% | 16.5% | - | - |
| **Status quo**** | **Nondaily** | 11.9% | 8.2% | 7.8% | 5.8% | -26.9% | -38.2% |
|  | **Daily** | 21.3% | 14.6% | 14.4% | 10.8% | -25.6% | -34.7% |
| **Individual policy effect** | | | | | | | |
| **Cigarette Price** | **Nondaily** | 11.9% | 9.5% | 9.0% | 7.1% | -16.0% | -25.2% |
|  | **Daily** | 21.3% | 16.8% | 16.6% | 13.0% | -14.4% | -21.3% |
| **Smoke-free air laws** | **Nondaily** | 11.9% | 10.9% | 9.8% | 9.1% | -2.8% | -3.3% |
|  | **Daily** | 21.3% | 19.1% | 19.0% | 16.0% | -2.7% | -3.2% |
| **Media campaigns** | **Nondaily** | 11.9% | 11.1% | 10.0% | 9.3% | -1.2% | -1.3% |
|  | **Daily** | 21.3% | 19.4% | 19.3% | 16.3% | -1.1% | -1.3% |
| **Cessation treatments** | **Nondaily** | 11.9% | 11.0% | 9.9% | 9.2% | -2.0% | -2.7% |
|  | **Daily** | 21.3% | 19.2% | 19.1% | 16.1% | -2.1% | -2.6% |
| **Health warnings** | **Nondaily** | 11.9% | 10.8% | 9.7% | 8.8% | -4.0% | -6.4% |
|  | **Daily** | 21.3% | 18.8% | 18.7% | 15.5% | -4.1% | -6.3% |
| **Marketing bans** | **Nondaily** | 11.9% | 11.1% | 9.9% | 9.3% | -1.7% | -2.2% |
|  | **Daily** | 21.3% | 19.3% | 19.2% | 16.2% | -1.7% | -2.1% |
| **Female** | | | | | | | |
| **Scenarios** | **Type of smoker** | **2002** | **2018** | **2019** | **2060** | **Relative difference** | |
|  |  |  |  |  |  | **in 2018** | **in 2060** |
| **Counterfactual*** | **Nondaily** | 4.2% | 4.1% | 3.7% | 3.5% | - | - |
|  | **Daily** | 6.9% | 6.0% | 6.0% | 4.5% | - | - |
| **Status quo**** | **Nondaily** | 4.2% | 3.0% | 2.9% | 2.1% | -27.3% | -38.6% |
|  | **Daily** | 6.9% | 4.4% | 4.3% | 2.7% | -26.7% | -38.6% |
| **Individual policy effect** | | | | | | | |
| **Cigarette Price** | **Nondaily** | 4.2% | 3.5% | 3.3% | 2.6% | -16.1% | -24.8% |
|  | **Daily** | 6.9% | 5.1% | 5.0% | 3.3% | -15.5% | -25.6% |
| **Smoke-free air laws** | **Nondaily** | 4.2% | 4.0% | 3.6% | 3.3% | -2.8% | -3.5% |
|  | **Daily** | 6.9% | 5.9% | 5.8% | 4.3% | -2.8% | -3.4% |
| **Media campaigns** | **Nondaily** | 4.2% | 4.1% | 3.7% | 3.4% | -1.2% | -1.4% |
|  | **Daily** | 6.9% | 6.0% | 5.9% | 4.4% | -1.2% | -1.3% |
| **Cessation treatments** | **Nondaily** | 4.2% | 4.0% | 3.6% | 3.3% | -2.2% | -3.1% |
|  | **Daily** | 6.9% | 5.9% | 5.8% | 4.3% | -2.2% | -2.6% |
| **Health warnings** | **Nondaily** | 4.2% | 4.0% | 3.6% | 3.2% | -4.1% | -6.8% |
|  | **Daily** | 6.9% | 5.8% | 5.7% | 4.2% | -4.1% | -6.5% |
| **Marketing bans** | **Nondaily** | 4.2% | 4.1% | 3.7% | 3.4% | -1.7% | -2.2% |
|  | **Daily** | 6.9% | 5.9% | 5.9% | 4.4% | -1.7% | -2.2% |

* The counterfactual is determined by keeping the policies at their 2002 levels

** The status quo is obtained incorporating policies implemented between 2002 and 2018**Table 4.1 Smoking-attributable deaths (SADs) and lives saved† by smoking status and gender, from Mexico *SimSmoke* under various scenarios in 2002-2060. Scenario 1, without 100-cigarettes screen and full policies effect.**

| **Male** | | | | | | | |
| --- | --- | --- | --- | --- | --- | --- | --- |
| **SADs** | **Type of smokers** | **2002** | **2018** | **2019** | **2060** | **Sum by 2018** | **Sum by 2060** |
| **Counterfactual*** | **Nondaily** | 5,271 | 7,875 | 8,142 | 15,280 | 107,956 | 626,813 |
|  | **Daily** | 50,032 | 59,140 | 60,539 | 104,037 | 888,193 | 4,501,584 |
|  | **Total** | 55,303 | 67,015 | 68,681 | 119,317 | 996,150 | 5,128,397 |
| **Status quo**** | **Nondaily** | 5,271 | 7,166 | 7,337 | 10,129 | 104,270 | 497,473 |
|  | **Daily** | 50,032 | 53,393 | 54,139 | 71,220 | 856,224 | 3,613,651 |
|  | **Total** | 55,303 | 60,559 | 61,476 | 81,349 | 960,494 | 4,111,124 |
| **Lives saved†** |  |  |  |  |  |  |  |
| **Status quo** | **Nondaily** | - | 709 | 804 | 5,151 | 3,686 | 129,340 |
|  | **Daily** | - | 5,747 | 6,400 | 32,817 | 31,970 | 887,933 |
|  | **Total** | - | 6,456 | 7,205 | 37,968 | 35,656 | 1,017,273 |
| **Cigarette price** | **Nondaily** | - | 343 | 377 | 2,943 | 1,760 | 64,730 |
|  | **Daily** | - | 2,739 | 2,952 | 18,566 | 15,068 | 446,159 |
|  | **Total** | - | 3,083 | 3,329 | 21,509 | 16,828 | 510,889 |
| **Smoke-free air laws** | **Nondaily** | - | 77 | 105 | 519 | 401 | 14,582 |
|  | **Daily** | - | 639 | 848 | 3,271 | 3,553 | 99,698 |
|  | **Total** | - | 716 | 953 | 3,790 | 3,954 | 114,281 |
| **Media campaigns** | **Nondaily** | - | 53 | 52 | 212 | 326 | 6,296 |
|  | **Daily** | - | 423 | 413 | 1,315 | 2,827 | 42,890 |
|  | **Total** | - | 476 | 465 | 1,527 | 3,153 | 49,186 |
| **Cessation treatments** | **Nondaily** | - | 74 | 92 | 641 | 348 | 17,379 |
|  | **Daily** | - | 598 | 730 | 3,602 | 3,034 | 109,019 |
|  | **Total** | - | 671 | 822 | 4,243 | 3,382 | 126,398 |
| **Health warnings** | **Nondaily** | - | 117 | 129 | 896 | 588 | 22,336 |
|  | **Daily** | - | 966 | 1,044 | 5,932 | 5,150 | 159,967 |
|  | **Total** | - | 1,083 | 1,174 | 6,827 | 5,738 | 182,303 |
| **Marketing bans** | **Nondaily** | - | 49 | 53 | 290 | 288 | 7,983 |
|  | **Daily** | - | 405 | 427 | 1,945 | 2,528 | 56,641 |
|  | **Total** | - | 454 | 479 | 2,236 | 2,816 | 64,624 |

| **Female** | | | | | | | |
| --- | --- | --- | --- | --- | --- | --- | --- |
| **SADs** | **Type of smokers** | **2002** | **2018** | **2019** | **2060** | **Sum by 2018** | **Sum by 2060** |
| **Counterfactual*** | **Nondaily** | 1,648 | 2,167 | 2,240 | 5,584 | 31,323 | 204,658 |
|  | **Daily** | 15,454 | 17,779 | 18,282 | 24,779 | 269,139 | 1,254,735 |
|  | **Total** | 17,102 | 19,946 | 20,522 | 30,362 | 300,463 | 1,459,394 |
| **Status quo**** | **Nondaily** | 1,648 | 1,977 | 2,025 | 3,733 | 30,340 | 161,799 |
|  | **Daily** | 15,454 | 16,204 | 16,524 | 16,474 | 260,412 | 1,024,366 |
|  | **Total** | 17,102 | 18,181 | 18,549 | 20,207 | 290,752 | 1,186,165 |
| **Lives saved†** |  |  |  |  |  |  |  |
| **Status quo** | **Nondaily** | - | 190 | 215 | 1,851 | 983 | 42,859 |
|  | **Daily** | - | 1,575 | 1,758 | 8,305 | 8,727 | 230,369 |
|  | **Total** | - | 1,765 | 1,973 | 10,156 | 9,710 | 273,228 |
| **Cigarette price** | **Nondaily** | - | 93 | 101 | 1,007 | 480 | 20,488 |
|  | **Daily** | - | 734 | 786 | 4,884 | 4,055 | 113,194 |
|  | **Total** | - | 827 | 888 | 5,891 | 4,535 | 133,682 |
| **Smoke-free air laws** | **Nondaily** | - | 20 | 27 | 192 | 103 | 4,877 |
|  | **Daily** | - | 174 | 235 | 822 | 973 | 26,123 |
|  | **Total** | - | 194 | 262 | 1,014 | 1,076 | 31,001 |
| **Media campaigns** | **Nondaily** | - | 14 | 14 | 79 | 84 | 2,102 |
|  | **Daily** | - | 117 | 112 | 337 | 770 | 11,385 |
|  | **Total** | - | 131 | 126 | 416 | 854 | 13,487 |
| **Cessation treatments** | **Nondaily** | - | 21 | 26 | 266 | 96 | 6,503 |
|  | **Daily** | - | 177 | 218 | 1,016 | 873 | 32,415 |
|  | **Total** | - | 198 | 243 | 1,282 | 969 | 38,918 |
| **Health warnings** | **Nondaily** | - | 31 | 34 | 337 | 155 | 7,640 |
|  | **Daily** | - | 269 | 294 | 1,408 | 1,422 | 41,200 |
|  | **Total** | - | 300 | 329 | 1,746 | 1,577 | 48,840 |
| **Marketing bans** | **Nondaily** | - | 13 | 14 | 105 | 74 | 2,613 |
|  | **Daily** | - | 109 | 115 | 459 | 684 | 14,150 |
|  | **Total** | - | 121 | 129 | 563 | 758 | 16,763 |

* The counterfactual is determined by keeping the policies at their 2002 levels

** The status quo is obtained incorporating policies implemented between 2002 and 2018

**†** Lives saved are calculated based on the difference in smoking-attributable deaths in the status quo and smoking-attributable deaths with the policy implemented. †No change in policy level since 2002

**Table 5. Prevalence by smoking status and gender, from Mexico *SimSmoke* under various stronger future policies, 2002-2060. Without 100-cigarettes screen and full policies effect.**

|  | **Male** | | | | **Female** | | | |
| --- | --- | --- | --- | --- | --- | --- | --- | --- |
| **Scenarios** | **2018** | **2019** | **2030** | **2060** | **2018** | **2019** | **2030** | **2060** |
| **Daily Smoking Prevalence** | | | | | | | | |
| **Status Quo Policies** | 14.6% | 14.4% | 12.9% | 10.8% | 4.4% | 4.3% | 3.6% | 2.7% |
| **Percent Change in Smoking Prevalence from Status Quo** | | | | | | | | |
| **Tax of 60% Price** | - | -3.6% | -4.8% | -6.7% | - | -3.9% | -5.2% | -8.0% |
| **Tax of 70% Price** | - | -10.5% | -13.4% | -18.3% | - | -11.3% | -14.6% | -21.5% |
| **Complete Smoke Free** | - | -2.9% | -3.4% | -3.8% | - | -2.9% | -3.4% | -3.9% |
| **Marketing Ban & Enforcement** | - | -3.1% | -4.1% | -5.8% | - | -3.1% | -4.0% | -5.8% |
| **Tobacco Control Campaign** | - | -5.6% | -6.7% | -7.7% | - | -5.6% | -6.8% | -7.9% |
| **Strong Health Warnings** | - | 0.0% | 0.0% | 0.0% | - | 0.0% | 0.0% | 0.0% |
| **Youth Access Enforcement** | - | -0.4% | -3.3% | -8.2% | - | -0.6% | -5.6% | -16.9% |
| **Cessation Treatment Policies** | - | -1.9% | -3.9% | -4.7% | - | -1.9% | -4.2% | -4.7% |
| **All above, with 60% tax** | - | -13.5% | -20.9% | -28.3% | - | -13.7% | -23.1% | -35.2% |
| **All above, with 70% tax** | - | -22.6% | -31.7% | -41.9% | - | -23.4% | -34.4% | -49.5% |
| **Nondaily Smoking Prevalence** | | | | | | | | |
| **Status Quo Policies** | 8.2% | 8.1% | 7.2% | 5.8% | 3.0% | 3.0% | 2.6% | 2.1% |
| **Percent Change in Smoking Prevalence from Status Quo** | | | | | | | | |
| **Tax of 60% Price** | - | -3.9% | -5.3% | -7.9% | - | -3.8% | -5.2% | -7.7% |
| **Tax of 70% Price** | - | -11.5% | -14.9% | -21.3% | - | -11.2% | -14.6% | -20.9% |
| **Complete Smoke Free** | - | -2.9% | -3.4% | -3.9% | - | -2.9% | -3.5% | -4.0% |
| **Marketing Ban & Enforcement** | - | -3.1% | -4.0% | -5.7% | - | -3.1% | -4.1% | -5.9% |
| **Tobacco Control Campaign** | - | -5.6% | -6.7% | -7.8% | - | -5.6% | -6.9% | -8.1% |
| **Strong Health Warnings** | - | 0.0% | 0.0% | 0.0% | - | 0.0% | 0.0% | 0.0% |
| **Youth Access Enforcement** | - | -0.8% | -6.5% | -18.0% | - | -0.6% | -5.0% | -13.8% |
| **Cessation Treatment Policies** | - | -1.9% | -3.9% | -4.7% | - | -1.9% | -4.3% | -5.3% |
| **All above, with 60% tax** | - | -13.8% | -23.5% | -36.1% | - | -13.7% | -22.8% | -33.7% |
| **All above, with 70% tax** | - | -23.6% | -35.0% | -50.3% | - | -23.3% | -34.2% | -47.8% |

**Table 6. Smoking-attributable deaths (SADs) and potential lives saved by smoking status and gender, from Mexico *SimSmoke* under various stronger future policies in 2002-2060. Scenario 1,without 100-cigarettes screen and full policies effect.**

|  | **Male** | | | | | **Female** | | | | |
| --- | --- | --- | --- | --- | --- | --- | --- | --- | --- | --- |
| **Scenarios** | **2018** | **2019** | **2030** | **2060** | **2019-2060** | **2018** | **2019** | **2030** | **2060** | **2019-2060** |
| **Daily smoking-attributable deaths** | | | | | | | | | | |
| **Status Quo Policies** | 53,393 | 54,139 | 61,402 | 71,215 | 2,757,343 | 16,204 | 16,524 | 18,958 | 16,474 | 763,954 |
| **Lives could be saved** | | | | | | | | | | |
| **Tax of 60% Price** | - | - | 1,062 | 3,169 | 68,753 | - | - | 303 | 718 | 16,459 |
| **Tax of 70% Price** | - | - | 3,109 | 8,827 | 195,589 | - | - | 885 | 2,002 | 46,902 |
| **Complete Smoke Free** | - | - | 1,126 | 2,378 | 63,665 | - | - | 311 | 540 | 15,757 |
| **Marketing Ban** | - | - | 1,166 | 2,866 | 69,860 | - | - | 320 | 580 | 16,179 |
| **Tobacco Control Campaign** | - | - | 2,153 | 4,968 | 129,173 | - | - | 596 | 1,145 | 32,251 |
| **Strong Health Warnings** | - | - | - | - | - | - | - | - | - | - |
| **Youth Access Enforcement** | - | - | - | 1,613 | 16,567 | - | - | - | 601 | 5,664 |
| **Cessation Treatment Policies** | - | - | 1,270 | 4,113 | 101,128 | - | - | 383 | 1,061 | 28,169 |
| **All above, with 60% tax** | - | - | 5,579 | 15,315 | 370,670 | - | - | 1,571 | 3,706 | 94,898 |
| **All above, with 70% tax** | - | - | 8,228 | 22,062 | 528,151 | - | - | 2,322 | 5,153 | 131,878 |
| **Nondaily smoking-attributable deaths** | | | | | | | | | | |
| **Status Quo Policies** | 7,166 | 7,337 | 8,923 | 10,143 | 393,585 | 1,977 | 2,025 | 2,571 | 3,738 | 131,580 |
| **Lives could be saved** | | | | | | | | | | |
| **Tax of 60% Price** | - | - | 155 | 456 | 9,850 | - | - | 39 | 149 | 3,031 |
| **Tax of 70% Price** | - | - | 451 | 1,271 | 27,997 | - | - | 115 | 415 | 8,616 |
| **Complete Smoke Free** | - | - | 151 | 350 | 9,022 | - | - | 42 | 125 | 3,037 |
| **Marketing Ban** | - | - | 156 | 383 | 9,400 | - | - | 43 | 133 | 3,124 |
| **Tobacco Control Campaign** | - | - | 289 | 739 | 18,416 | - | - | 80 | 268 | 6,274 |
| **Strong Health Warnings** | - | - | - | - | - | - | - | - | - | - |
| **Youth Access Enforcement** | - | - | - | 427 | 4,303 | - | - | - | 84 | 798 |
| **Cessation Treatment Policies** | - | - | 176 | 672 | 15,517 | - | - | 50 | 266 | 5,780 |
| **All above, with 60% tax** | - | - | 752 | 2,429 | 54,735 | - | - | 209 | 833 | 18,404 |
| **All above, with 70% tax** | - | - | 1,135 | 3,352 | 76,728 | - | - | 307 | 1,136 | 25,172 |

**Table 2.2 Validation of daily and nondaily smoking prevalence, ages 15-65 by gender, from Mexico *SimSmoke* and various national surveys*, 2002-2016. Scenario 2, without 100-cigarettes screen and half policies effect for nondaily.**

|  | | | **Male** | | | | | | **Relative difference** | |
| --- | --- | --- | --- | --- | --- | --- | --- | --- | --- | --- |
| **Daily** | **Age groups** | | **2002** | **2009** | **2011** | **2012** | **2015** | **2016** | **2002-2011** | **2002-2016** |
|  | 15-65 | SimSmoke | 21.9% | 18.4% | 16.7% | 16.5% | 15.5% | 15.3% | -23.5% | -29.9% |
|  |  | Surveys | 21.6% | 12.0% | 11.6% | 16.0% | 12.1% | 11.0% | -46.3% | -49.1% |
|  |  | 95% CI | 19.6%,23.8% | 10.7%,13.4% | 10.4%,13.0% | 15.2%,16.9% | 10.8%,13.6% | 10.2%,11.9% |  |  |
|  | 15-24 | SimSmoke | 15.8% | 11.7% | 10.4% | 10.2% | 9.9% | 9.9% | -34.4% | -37.2% |
|  |  | Surveys | 15.1% | 10.9% | 6.6% | 12.3% | 10.4% | 6.9% | -56.3% | -54.3% |
|  |  | 95% CI | 12.0%,18.7% | 8.4%,14.1% | 5.2%,8.3% | 11.1%,13.6% | 8.2%,13.1% | 5.8%,8.2% |  |  |
|  | 25-44 | SimSmoke | 26.6% | 22.0% | 19.8% | 19.4% | 17.9% | 17.7% | -25.5% | -33.5% |
|  |  | Surveys | 26.9% | 11.5% | 13.0% | 19.2% | 12.8% | 12.5% | -51.7% | -53.5% |
|  |  | 95% CI | 23.7%,30.3% | 9.7%,13.6% | 11.2%,15.0% | 18.0%,20.5% | 10.8%,15.1% | 11.0%,14.1% |  |  |
|  | 45-65 | SimSmoke | 20.8% | 19.8% | 18.5% | 18.3% | 17.4% | 17.1% | -11.1% | -17.9% |
|  |  | Surveys | 20.9% | 14.2% | 15.4% | 18.8% | 12.9% | 13.2% | -26.3% | -36.8% |
|  |  | 95% CI | 17.1%,25.2% | 12.1%,16.6% | 12.6%,18.6% | 17.3%,20.4% | 10.6%,15.5% | 11.8%,14.6% |  |  |
| **Non-daily** | **Age groups** | | **2002** | **2009** | **2011** | **2012** | **2015** | **2016** | **2002-2011** | **2002-2016** |
|  | 15-65 | SimSmoke | 12.3% | 11.2% | 10.6% | 10.5% | 10.1% | 10.0% | -13.7% | -19.0% |
|  |  | Surveys | 12.3% | 13.8% | 15.6% | 15.7% | 14.1% | 18.4% | 26.8% | 49.6% |
|  |  | 95% CI | 10.9%,13.9% | 12.6%,15.2% | 14.2%,17.0% | 14.9%,16.5% | 12.8%,15.5% | 17.4%,19.5% |  |  |
|  | 15-24 | SimSmoke | 12.6% | 10.6% | 10.0% | 9.9% | 9.7% | 9.7% | -20.8% | -22.7% |
|  |  | Surveys | 12.4% | 15.8% | 19.0% | 15.9% | 17.1% | 20.7% | 53.2% | 66.9% |
|  |  | 95% CI | 9.6%,15.9% | 13.5%,18.4% | 16.3%,21.9% | 14.6%,17.4% | 14.4%,20.1% | 18.9%,22.7% |  |  |
|  | 25-44 | SimSmoke | 11.9% | 11.2% | 10.7% | 10.7% | 10.4% | 10.4% | -9.9% | -13.0% |
|  |  | Surveys | 11.8% | 15.1% | 17.4% | 18.9% | 15.4% | 20.7% | 47.5% | 75.4% |
|  |  | 95% CI | 9.6%,14.5% | 13.4%,16.9% | 15.2%,19.7% | 17.8%,20.1% | 13.5%,17.5% | 19.1%,22.3% |  |  |
|  | 45-65 | SimSmoke | 12.9% | 12.0% | 11.3% | 11.0% | 10.0% | 9.7% | -12.6% | -24.8% |
|  |  | Surveys | 13.0% | 8.9% | 8.7% | 10.4% | 9.0% | 13.2% | -33.1% | 1.5% |
|  |  | 95% CI | 9.7%,17.1% | 7.0%,11.4% | 7.1%,10.7% | 9.3%,11.5% | 7.3%,11.1% | 11.6%,15.0% |  |  |

|  | | | **Female** | | | | | | **Relative difference** | |
| --- | --- | --- | --- | --- | --- | --- | --- | --- | --- | --- |
| **Daily** | **Age groups** | | **2002** | **2009** | **2011** | **2012** | **2015** | **2016** | **2002-2011** | **2002-2016** |
|  | 15-65 | SimSmoke | 7.1% | 5.9% | 5.2% | 5.1% | 4.7% | 4.6% | -26.4% | -34.9% |
|  |  | Surveys | 7.1% | 3.8% | 3.8% | 5.4% | 3.9% | 3.3% | -46.5% | -53.5% |
|  |  | 95% CI | 5.9%,8.6% | 3.0%,4.8% | 3.3%,4.5% | 4.9%,5.8% | 3.2%,4.7% | 3.0%,3.7% |  |  |
|  | 15-24 | SimSmoke | 6.6% | 5.2% | 4.6% | 4.5% | 4.4% | 4.4% | -30.4% | -34.1% |
|  |  | Surveys | 6.7% | 2.4% | 2.2% | 3.1% | 2.4% | 2.0% | -67.2% | -70.1% |
|  |  | 95% CI | 4.7%,9.6% | 1.5%,3.6% | 1.4%,3.4% | 2.5%,3.8% | 1.5%,3.7% | 1.5%,2.7% |  |  |
|  | 25-44 | SimSmoke | 7.1% | 5.5% | 4.8% | 4.7% | 4.4% | 4.3% | -31.5% | -38.6% |
|  |  | Surveys | 7.0% | 3.7% | 4.3% | 6.2% | 3.8% | 3.3% | -38.6% | -52.9% |
|  |  | 95% CI | 5.6%,8.8% | 2.7%,4.9% | 3.5%,5.2% | 5.5%,7.0% | 3.0%,4.9% | 2.8%,3.9% |  |  |
|  | 45-65 | SimSmoke | 7.9% | 7.2% | 6.6% | 6.4% | 5.6% | 5.3% | -16.6% | -32.4% |
|  |  | Surveys | 7.9% | 5.9% | 4.9% | 7.8% | 5.2% | 4.6% | -38.0% | -41.8% |
|  |  | 95% CI | 6.0%,10.2% | 4.5%,7.5% | 3.7%,6.4% | 6.9%,8.8% | 3.5%,7.6% | 4.0%,5.3% |  |  |
| **Non-daily** | **Age groups** | | **2002** | **2009** | **2011** | **2012** | **2015** | **2016** | **2002-2011** | **2002-2016** |
|  | 15-65 | SimSmoke | 4.4% | 4.1% | 3.9% | 3.9% | 3.8% | 3.8% | -10.7% | -14.4% |
|  |  | Surveys | 4.3% | 4.4% | 6.1% | 5.5% | 4.9% | 6.0% | 41.9% | 39.5% |
|  |  | 95% CI | 3.4%,5.5% | 3.6%,5.3% | 5.3%,7.0% | 5.1%,6.0% | 4.2%,5.8% | 5.5%,6.6% |  |  |
|  | 15-24 | SimSmoke | 4.7% | 4.0% | 3.8% | 3.8% | 3.7% | 3.7% | -18.5% | -20.1% |
|  |  | Surveys | 4.7% | 5.1% | 8.2% | 6.2% | 5.1% | 7.0% | 74.5% | 48.9% |
|  |  | 95% CI | 3.2%,6.8% | 3.7%,6.9% | 6.3%,10.6% | 5.4%,7.0% | 3.6%,7.0% | 6.0%,8.2% |  |  |
|  | 25-44 | SimSmoke | 5.2% | 4.5% | 4.2% | 4.1% | 4.0% | 4.0% | -19.3% | -23.9% |
|  |  | Surveys | 5.2% | 4.7% | 5.6% | 5.8% | 6.2% | 6.9% | 7.7% | 32.7% |
|  |  | 95% CI | 3.8%,7.2% | 3.7%,5.9% | 4.6%,6.8% | 5.2%,6.6% | 5.0%,7.6% | 6.1%,7.8% |  |  |
|  | 45-65 | SimSmoke | 2.4% | 3.5% | 3.6% | 3.6% | 3.5% | 3.5% | 51.0% | 47.6% |
|  |  | Surveys | 2.2% | 2.9% | 4.8% | 3.9% | 2.8% | 3.7% | 118.2% | 68.2% |
|  |  | 95% CI | 1.4%,3.3% | 1.9%,4.3% | 3.6%,6.3% | 3.3%,4.7% | 2.0%,4.0% | 3.1%,4.5% |  |  |

*The survey point estimates (indicated by dots) and 95% confidence intervals were reported from various surveys, including the Encuesta Nacional de Adicciones (ENA) in 2002 and 2011, the Global Adult Tobacco Survey (GATS) in 2009 and 2015, the Encuesta Nacional de Salud y Nutricion (ENSANUT) in 2012, and the Encuesta Nacional de Consumo de Drogas, Alcohol y Tabaco (ENCODAT) in 2016.

**Table 3.2 Prevalence by smoking status and gender, from Mexico *SimSmoke* under various scenarios in 2002-2060. Scenario 2, without 100-cigarettes screen and half policies effect for nondaily.**

| **Male** | | | | | | | |
| --- | --- | --- | --- | --- | --- | --- | --- |
| **Scenarios** | **Type of smoker** | **2002** | **2018** | **2019** | **2060** | **Relative difference** | |
|  |  |  |  |  |  | **in 2018** | **in 2060** |
| **Counterfactual*** | **Nondaily** | 11.9% | 11.3% | 10.6% | 9.5% | - | - |
|  | **Daily** | 21.3% | 19.6% | 19.5% | 16.5% | - | - |
| **Status quo**** | **Nondaily** | 11.9% | 9.6% | 9.4% | 7.5% | -14.3% | -21.1% |
|  | **Daily** | 21.3% | 14.5% | 14.4% | 10.6% | -25.8% | -35.6% |
| **Individual policy effect** | | | | | | | |
| **Cigarette Price** | **Nondaily** | 11.9% | 10.3% | 10.0% | 8.2% | -8.3% | -13.4% |
|  | **Daily** | 21.3% | 16.7% | 16.6% | 12.9% | -14.6% | -22.1% |
| **Smoke-free air laws** | **Nondaily** | 11.9% | 11.1% | 10.5% | 9.3% | -1.4% | -1.7% |
|  | **Daily** | 21.3% | 19.1% | 19.0% | 16.0% | -2.8% | -3.3% |
| **Media campaigns** | **Nondaily** | 11.9% | 11.2% | 10.6% | 9.4% | -0.6% | -0.7% |
|  | **Daily** | 21.3% | 19.4% | 19.3% | 16.3% | -1.2% | -1.3% |
| **Cessation treatments** | **Nondaily** | 11.9% | 11.1% | 10.5% | 9.3% | -1.0% | -1.4% |
|  | **Daily** | 21.3% | 19.2% | 19.1% | 16.1% | -2.1% | -2.6% |
| **Health warnings** | **Nondaily** | 11.9% | 11.0% | 10.4% | 9.2% | -2.0% | -3.3% |
|  | **Daily** | 21.3% | 18.8% | 18.7% | 15.4% | -4.1% | -6.5% |
| **Marketing bans** | **Nondaily** | 11.9% | 11.2% | 10.5% | 9.4% | -0.8% | -1.1% |
|  | **Daily** | 21.3% | 19.3% | 19.2% | 16.1% | -1.7% | -2.2% |
| **Female** | | | | | | | |
| **Scenarios** | **Type of smoker** | **2002** | **2018** | **2019** | **2060** | **Relative difference** | |
|  |  |  |  |  |  | **in 2018** | **in 2060** |
| **Counterfactual*** | **Nondaily** | 4.2% | 4.1% | 3.9% | 3.5% | - | - |
|  | **Daily** | 6.9% | 6.0% | 6.0% | 4.5% | - | - |
| **Status quo**** | **Nondaily** | 4.2% | 3.5% | 3.4% | 2.7% | -14.6% | -21.1% |
|  | **Daily** | 6.9% | 4.4% | 4.3% | 2.7% | -26.7% | -38.6% |
| **Individual policy effect** | | | | | | | |
| **Cigarette Price** | **Nondaily** | 4.2% | 3.8% | 3.7% | 3.0% | -8.4% | -13.0% |
|  | **Daily** | 6.9% | 5.1% | 5.0% | 3.3% | -15.6% | -25.6% |
| **Smoke-free air laws** | **Nondaily** | 4.2% | 4.1% | 3.9% | 3.4% | -1.4% | -1.7% |
|  | **Daily** | 6.9% | 5.9% | 5.8% | 4.3% | -2.8% | -3.4% |
| **Media campaigns** | **Nondaily** | 4.2% | 4.1% | 3.9% | 3.4% | -0.6% | -0.7% |
|  | **Daily** | 6.9% | 6.0% | 5.9% | 4.4% | -1.2% | -1.3% |
| **Cessation treatments** | **Nondaily** | 4.2% | 4.1% | 3.9% | 3.4% | -1.1% | -1.6% |
|  | **Daily** | 6.9% | 5.9% | 5.8% | 4.3% | -2.2% | -2.6% |
| **Health warnings** | **Nondaily** | 4.2% | 4.0% | 3.8% | 3.3% | -2.1% | -3.4% |
|  | **Daily** | 6.9% | 5.8% | 5.7% | 4.2% | -4.1% | -6.6% |
| **Marketing bans** | **Nondaily** | 4.2% | 4.1% | 3.9% | 3.4% | -0.8% | -1.1% |
|  | **Daily** | 6.9% | 5.9% | 5.9% | 4.4% | -1.7% | -2.2% |

* The counterfactual is determined by keeping the policies at their 2002 levels**.** ** The status quo is obtained incorporating policies implemented between 2002 and 2018**Table 4.2 Smoking-attributable deaths (SADs) and lives saved† by smoking status and gender, from Mexico *SimSmoke* under various scenarios in 2002-2060. Scenario 2, without 100-cigarettes screen and half policies effect for nondaily.**

| **Male** | | | | | | | |
| --- | --- | --- | --- | --- | --- | --- | --- |
| **SADs** | **Type of smokers** | **2002** | **2018** | **2019** | **2060** | **Sum by 2018** | **Sum by 2060** |
| **Counterfactual*** | **Nondaily** | 5,271 | 7,876 | 8,143 | 15,314 | 107,962 | 627,635 |
|  | **Daily** | 50,032 | 59,140 | 60,539 | 104,037 | 888,193 | 4,501,564 |
|  | **Total** | 55,303 | 67,016 | 68,682 | 119,351 | 996,155 | 5,129,198 |
| **Status quo**** | **Nondaily** | 5,271 | 7,503 | 7,720 | 12,459 | 106,043 | 557,466 |
|  | **Daily** | 50,032 | 53,385 | 54,129 | 70,702 | 856,183 | 3,605,589 |
|  | **Total** | 55,303 | 60,888 | 61,848 | 83,161 | 962,226 | 4,163,055 |
| **Lives saved†** |  |  |  |  |  |  |  |
| **Status quo** | **Nondaily** | - | 372 | 423 | 2,855 | 1,919 | 70,169 |
|  | **Daily** | - | 5,756 | 6,410 | 33,335 | 32,010 | 895,974 |
|  | **Total** | - | 6,128 | 6,834 | 36,190 | 33,929 | 966,143 |
| **Cigarette price** | **Nondaily** | - | 177 | 194 | 1,573 | 901 | 34,232 |
|  | **Daily** | - | 2,739 | 2,953 | 19,063 | 15,061 | 453,386 |
|  | **Total** | - | 2,916 | 3,147 | 20,636 | 15,962 | 487,618 |
| **Smoke-free air laws** | **Nondaily** | - | 39 | 53 | 263 | 201 | 7,382 |
|  | **Daily** | - | 639 | 848 | 3,335 | 3,552 | 100,583 |
|  | **Total** | - | 678 | 901 | 3,598 | 3,754 | 107,965 |
| **Media campaigns** | **Nondaily** | - | 27 | 26 | 107 | 164 | 3,173 |
|  | **Daily** | - | 423 | 413 | 1,347 | 2,827 | 43,381 |
|  | **Total** | - | 450 | 439 | 1,454 | 2,991 | 46,554 |
| **Cessation treatments** | **Nondaily** | - | 37 | 46 | 329 | 175 | 8,850 |
|  | **Daily** | - | 603 | 736 | 3,622 | 3,068 | 109,693 |
|  | **Total** | - | 641 | 783 | 3,951 | 3,243 | 118,543 |
| **Health warnings** | **Nondaily** | - | 59 | 65 | 457 | 296 | 11,356 |
|  | **Daily** | - | 969 | 1,049 | 6,050 | 5,167 | 161,782 |
|  | **Total** | - | 1,028 | 1,114 | 6,507 | 5,462 | 173,138 |
| **Marketing bans** | **Nondaily** | - | 25 | 27 | 146 | 144 | 4,025 |
|  | **Daily** | - | 405 | 427 | 1,995 | 2,526 | 57,324 |
|  | **Total** | - | 429 | 453 | 2,142 | 2,670 | 61,348 |

| **Female** | | | | | | | |
| --- | --- | --- | --- | --- | --- | --- | --- |
| **SADs** | **Type of smokers** | **2002** | **2018** | **2019** | **2060** | **Sum by 2018** | **Sum by 2060** |
| **Counterfactual*** | **Nondaily** | 1,648 | 2,167 | 2,240 | 5,596 | 31,325 | 204,932 |
|  | **Daily** | 15,454 | 17,779 | 18,282 | 24,778 | 269,139 | 1,254,719 |
|  | **Total** | 17,102 | 19,946 | 20,522 | 30,374 | 300,464 | 1,459,651 |
| **Status quo**** | **Nondaily** | 1,648 | 2,067 | 2,127 | 4,574 | 30,813 | 181,730 |
|  | **Daily** | 15,454 | 16,202 | 16,523 | 16,495 | 260,402 | 1,024,591 |
|  | **Total** | 17,102 | 18,270 | 18,650 | 21,069 | 291,214 | 1,206,321 |
| **Lives saved†** |  |  |  |  |  |  |  |
| **Status quo** | **Nondaily** | - | 100 | 113 | 1,022 | 512 | 23,202 |
|  | **Daily** | - | 1,577 | 1,759 | 8,283 | 8,737 | 230,128 |
|  | **Total** | - | 1,676 | 1,872 | 9,305 | 9,249 | 253,330 |
| **Cigarette price** | **Nondaily** | - | 48 | 52 | 533 | 246 | 10,754 |
|  | **Daily** | - | 733 | 785 | 4,873 | 4,051 | 113,004 |
|  | **Total** | - | 781 | 837 | 5,406 | 4,296 | 123,758 |
| **Smoke-free air laws** | **Nondaily** | - | 10 | 14 | 97 | 52 | 2,458 |
|  | **Daily** | - | 174 | 235 | 817 | 972 | 26,058 |
|  | **Total** | - | 184 | 248 | 914 | 1,024 | 28,516 |
| **Media campaigns** | **Nondaily** | - | 7 | 7 | 40 | 42 | 1,053 |
|  | **Daily** | - | 117 | 112 | 335 | 770 | 11,356 |
|  | **Total** | - | 124 | 119 | 374 | 812 | 12,409 |
| **Cessation treatments** | **Nondaily** | - | 10 | 13 | 136 | 48 | 3,317 |
|  | **Daily** | - | 179 | 220 | 1,021 | 884 | 32,556 |
|  | **Total** | - | 189 | 233 | 1,157 | 932 | 35,873 |
| **Health warnings** | **Nondaily** | - | 16 | 17 | 171 | 78 | 3,874 |
|  | **Daily** | - | 270 | 295 | 1,408 | 1,427 | 41,235 |
|  | **Total** | - | 286 | 313 | 1,579 | 1,505 | 45,109 |
| **Marketing bans** | **Nondaily** | - | 6 | 7 | 52 | 37 | 1,309 |
|  | **Daily** | - | 109 | 115 | 456 | 684 | 14,105 |
|  | **Total** | - | 115 | 122 | 508 | 720 | 15,414 |

* The counterfactual is determined by keeping the policies at their 2002 levels

** The status quo is obtained incorporating policies implemented between 2002 and 2018

**†** Lives saved are calculated based on the difference in smoking-attributable deaths in the status quo and smoking-attributable deaths with the policy implemented. †No change in policy level since 2002

**Table 2.3 Validation of daily and nondaily smoking prevalence, ages 15-65 by gender, from Mexico *SimSmoke* and various national surveys*, 2002-2016. Scenario 3, with 100-cigarettes screen and full policies effect.**

|  | | | **Male** | | | | | **Relative difference** | |
| --- | --- | --- | --- | --- | --- | --- | --- | --- | --- |
| **Daily** | **Age groups** | | **2002** | **2006** | **2011** | **2012** | **2016** | **2002-2011** | **2002-2016** |
|  | 15-65 | SimSmoke | 19.6% | 18.3% | 15.2% | 14.9% | 14.0% | -22.7% | -28.6% |
|  |  | Surveys | 19.8% | 20.6% | 11.4% | 15.0% | 10.5% | -42.4% | -47.0% |
|  |  | 95% CI | 17.7%,22.0% | 19.6%,21.5% | 10.2%,12.7% | 14.2%,15.8% | 9.7%,11.4% |  |  |
|  | 15-24 | SimSmoke | 12.7% | 11.3% | 8.8% | 8.7% | 8.3% | -30.6% | -34.4% |
|  |  | Surveys | 12.8% | 16.2% | 6.4% | 10.8% | 6.5% | -50.0% | -49.2% |
|  |  | 95% CI | 10.0%,16.3% | 14.8%,17.7% | 5.0%,8.1% | 9.7%,12.0% | 5.5%,7.8% |  |  |
|  | 25-44 | SimSmoke | 24.2% | 22.2% | 18.3% | 18.0% | 16.6% | -24.2% | -31.4% |
|  |  | Surveys | 24.7% | 22.3% | 12.7% | 18.5% | 12.0% | -48.6% | -51.4% |
|  |  | 95% CI | 21.3%,28.4% | 21.0%,23.7% | 10.9%,14.6% | 17.3%,19.8% | 10.5%,13.5% |  |  |
|  | 45-65 | SimSmoke | 20.0% | 19.5% | 16.8% | 16.6% | 15.6% | -16.2% | -22.1% |
|  |  | Surveys | 20.0% | 22.8% | 15.2% | 18.4% | 12.6% | -24.0% | -37.0% |
|  |  | 95% CI | 16.4%,24.2% | 21.1%,24.5% | 12.5%,18.4% | 16.9%,20.0% | 11.3%,14.0% |  |  |
| **Nondaily** | **Age groups** | | **2002** | **2006** | **2011** | **2012** | **2016** | **2002-2011** | **2002-2016** |
|  | 15-65 | SimSmoke | 6.8% | 6.2% | 5.0% | 4.9% | 4.5% | -26.2% | -33.9% |
|  |  | Surveys | 6.9% | 9.5% | 10.0% | 10.7% | 12.3% | 44.9% | 78.3% |
|  |  | 95% CI | 5.9%,8.0% | 8.9%,10.2% | 9.0%,11.1% | 10.0%,11.3% | 11.5%,13.2% |  |  |
|  | 15-24 | SimSmoke | 3.6% | 3.2% | 2.5% | 2.4% | 2.4% | -31.0% | -34.7% |
|  |  | Surveys | 3.6% | 10.9% | 9.3% | 8.7% | 11.1% | 158.3% | 208.3% |
|  |  | 95% CI | 2.6%,5.0% | 9.7%,12.2% | 7.6%,11.4% | 7.7%,9.7% | 9.7%,12.6% |  |  |
|  | 25-44 | SimSmoke | 7.6% | 6.8% | 5.6% | 5.5% | 5.0% | -26.7% | -34.5% |
|  |  | Surveys | 7.7% | 10.4% | 12.8% | 14.8% | 14.7% | 66.2% | 90.9% |
|  |  | 95% CI | 6.0%,9.8% | 9.4%,11.5% | 11.1%,14.6% | 13.8%,15.8% | 13.5%,16.1% |  |  |
|  | 45-65 | SimSmoke | 9.6% | 8.7% | 6.8% | 6.6% | 5.8% | -28.8% | -39.4% |
|  |  | Surveys | 9.9% | 6.6% | 6.3% | 8.6% | 10.4% | -36.4% | 5.1% |
|  |  | 95% CI | 7.2%,13.5% | 5.7%,7.8% | 5.0%,8.0% | 7.6%,9.6% | 9.0%,12.1% |  |  |

|  | | | **Female** | | | | | **Relative difference** | |
| --- | --- | --- | --- | --- | --- | --- | --- | --- | --- |
| **Daily** | **Age groups** | | **2002** | **2006** | **2011** | **2012** | **2016** | **2002-2011** | **2002-2016** |
|  | 15-65 | SimSmoke | 6.1% | 5.7% | 4.6% | 4.5% | 4.2% | -24.6% | -32.3% |
|  |  | Surveys | 6.3% | 6.3% | 3.7% | 5.0% | 3.2% | -41.3% | -49.2% |
|  |  | 95% CI | 5.2%,7.6% | 5.7%,6.8% | 3.1%,4.3% | 4.6%,5.4% | 2.8%,3.5% |  |  |
|  | 15-24 | SimSmoke | 5.4% | 4.8% | 3.8% | 3.7% | 3.6% | -29.7% | -34.0% |
|  |  | Surveys | 5.6% | 5.1% | 2.1% | 2.5% | 1.9% | -62.5% | -66.1% |
|  |  | 95% CI | 3.7%,8.4% | 4.2%,6.3% | 1.3%,3.3% | 2.0%,3.1% | 1.4%,2.6% |  |  |
|  | 25-44 | SimSmoke | 6.2% | 5.7% | 4.7% | 4.6% | 4.2% | -24.5% | -31.7% |
|  |  | Surveys | 6.2% | 6.4% | 4.1% | 5.9% | 3.2% | -33.9% | -48.4% |
|  |  | 95% CI | 4.9%,7.9% | 5.8%,7.2% | 3.4%,5.1% | 5.2%,6.7% | 2.7%,3.8% |  |  |
|  | 45-65 | SimSmoke | 7.0% | 6.8% | 5.4% | 5.2% | 4.5% | -22.9% | -34.8% |
|  |  | Surveys | 7.1% | 7.2% | 4.5% | 7.5% | 4.2% | -36.6% | -40.8% |
|  |  | 95% CI | 5.4%,9.3% | 6.2%,8.4% | 3.4%,5.9% | 6.6%,8.5% | 3.6%,4.9% |  |  |
| **Nondaily** | **Age groups** | | **2002** | **2006** | **2011** | **2012** | **2016** | **2002-2011** | **2002-2016** |
|  | 15-65 | SimSmoke | 1.6% | 1.5% | 1.3% | 1.3% | 1.2% | -21.8% | -28.3% |
|  |  | Surveys | 1.7% | 3.5% | 3.4% | 3.4% | 3.6% | 100.0% | 111.8% |
|  |  | 95% CI | 1.2%,2.2% | 3.1%,4.0% | 2.8%,4.1% | 3.1%,3.8% | 3.2%,4.0% |  |  |
|  | 15-24 | SimSmoke | 1.2% | 1.1% | 0.8% | 0.8% | 0.8% | -29.6% | -33.6% |
|  |  | Surveys | 1.2% | 4.3% | 4.1% | 3.2% | 3.5% | 241.7% | 191.7% |
|  |  | 95% CI | 0.7%,2.1% | 3.7%,5.1% | 2.9%,5.8% | 2.6%,4.0% | 2.8%,4.5% |  |  |
|  | 25-44 | SimSmoke | 2.0% | 1.8% | 1.4% | 1.4% | 1.3% | -26.5% | -34.3% |
|  |  | Surveys | 2.0% | 3.8% | 3.3% | 4.0% | 4.4% | 65.0% | 120.0% |
|  |  | 95% CI | 1.3%,3.1% | 3.1%,4.6% | 2.5%,4.2% | 3.4%,4.7% | 3.8%,5.1% |  |  |
|  | 45-65 | SimSmoke | 1.6% | 1.6% | 1.4% | 1.4% | 1.3% | -7.4% | -14.9% |
|  |  | Surveys | 1.4% | 2.2% | 3.0% | 2.8% | 2.3% | 114.3% | 64.3% |
|  |  | 95% CI | 0.9%,2.3% | 1.7%,2.9% | 2.0%,4.3% | 2.3%,3.4% | 1.9%,2.8% |  |  |

*The survey point estimates (indicated by dots) and 95% confidence intervals were reported from various surveys, including the Encuesta Nacional de Adicciones (ENA) in 2002 and 2011, the Encuesta Nacional de Salud y Nutricion (ENSANUT) in 2006 and 2012, and the Encuesta Nacional de Consumo de Drogas, Alcohol y Tabaco (ENCODAT) in 2016.

**Table 3.3 Prevalence by smoking status and gender, from Mexico *SimSmoke* under various scenarios in 2002-2060. Scenario 3, with 100-cigarettes screen and full policies effect.**

| **Male** | | | | | | | |
| --- | --- | --- | --- | --- | --- | --- | --- |
| **Scenarios** | **Type of smoker** | **2002** | **2018** | **2019** | **2060** | **Relative difference** | |
|  |  |  |  |  |  | **in 2018** | **in 2060** |
| **Counterfactual*** | **Nondaily** | 6.7% | 6.0% | 5.9% | 4.8% | - | - |
|  | **Daily** | 19.2% | 18.0% | 18.0% | 15.7% | - | - |
| **Status quo**** | **Nondaily** | 6.7% | 4.5% | 4.4% | 3.2% | -25.1% | -34.6% |
|  | **Daily** | 19.2% | 13.4% | 13.2% | 10.2% | -25.8% | -35.4% |
| **Individual policy effect** | | | | | | | |
| **Cigarette Price** | **Nondaily** | 6.7% | 5.1% | 5.1% | 3.8% | -13.6% | -20.5% |
|  | **Daily** | 19.2% | 15.4% | 15.3% | 12.3% | -14.5% | -21.5% |
| **Smoke-free air laws** | **Nondaily** | 6.7% | 5.8% | 5.7% | 4.7% | -2.8% | -3.3% |
|  | **Daily** | 19.2% | 17.5% | 17.4% | 15.2% | -2.8% | -3.4% |
| **Media campaigns** | **Nondaily** | 6.7% | 5.9% | 5.8% | 4.8% | -1.2% | -1.3% |
|  | **Daily** | 19.2% | 17.8% | 17.7% | 15.5% | -1.2% | -1.3% |
| **Cessation treatments** | **Nondaily** | 6.7% | 5.8% | 5.8% | 4.7% | -2.2% | -2.9% |
|  | **Daily** | 19.2% | 17.7% | 17.6% | 15.3% | -2.0% | -2.6% |
| **Health warnings** | **Nondaily** | 6.7% | 5.7% | 5.7% | 4.5% | -4.1% | -6.5% |
|  | **Daily** | 19.2% | 17.3% | 17.2% | 14.7% | -4.2% | -6.6% |
| **Marketing bans** | **Nondaily** | 6.7% | 5.9% | 5.8% | 4.7% | -1.6% | -2.1% |
|  | **Daily** | 19.2% | 17.7% | 17.6% | 15.4% | -1.7% | -2.2% |
| **Female** | | | | | | | |
| **Scenarios** | **Type of smoker** | **2002** | **2018** | **2019** | **2060** | **Relative difference** | |
|  |  |  |  |  |  | **in 2018** | **in 2060** |
| **Counterfactual*** | **Nondaily** | 1.7% | 1.5% | 1.5% | 1.3% | - | - |
|  | **Daily** | 5.9% | 5.4% | 5.4% | 4.2% | - | - |
| **Status quo**** | **Nondaily** | 1.7% | 1.1% | 1.1% | 0.8% | -26.5% | -37.3% |
|  | **Daily** | 5.9% | 4.0% | 3.9% | 2.6% | -26.6% | -37.6% |
| **Individual policy effect** | | | | | | | |
| **Cigarette Price** | **Nondaily** | 1.7% | 1.3% | 1.3% | 1.0% | -14.8% | -22.5% |
|  | **Daily** | 5.9% | 4.6% | 4.5% | 3.2% | -15.3% | -23.9% |
| **Smoke-free air laws** | **Nondaily** | 1.7% | 1.5% | 1.5% | 1.2% | -2.8% | -3.6% |
|  | **Daily** | 5.9% | 5.3% | 5.2% | 4.1% | -2.8% | -3.5% |
| **Media campaigns** | **Nondaily** | 1.7% | 1.5% | 1.5% | 1.2% | -1.2% | -1.4% |
|  | **Daily** | 5.9% | 5.4% | 5.3% | 4.2% | -1.2% | -1.4% |
| **Cessation treatments** | **Nondaily** | 1.7% | 1.5% | 1.5% | 1.2% | -2.3% | -3.3% |
|  | **Daily** | 5.9% | 5.3% | 5.3% | 4.1% | -2.1% | -2.7% |
| **Health warnings** | **Nondaily** | 1.7% | 1.5% | 1.5% | 1.2% | -4.3% | -7.1% |
|  | **Daily** | 5.9% | 5.2% | 5.1% | 4.0% | -4.1% | -6.7% |
| **Marketing bans** | **Nondaily** | 1.7% | 1.5% | 1.5% | 1.2% | -1.7% | -2.3% |
|  | **Daily** | 5.9% | 5.3% | 5.3% | 4.1% | -1.7% | -2.3% |

* The counterfactual is determined by keeping the policies at their 2002 levels

** The status quo is obtained incorporating policies implemented between 2002 and 2018**Table 4.3 Smoking-attributable deaths (SADs) and lives saved† by smoking status and gender, from Mexico *SimSmoke* under various scenarios in 2002-2060. Scenario 3, with 100-cigarettes screen and full policies effect.**

| **Male** | | | | | | | |
| --- | --- | --- | --- | --- | --- | --- | --- |
| **SADs** | **Type of smokers** | **2002** | **2018** | **2019** | **2060** | **Sum by 2018** | **Sum by 2060** |
| **Counterfactual*** | **Nondaily** | 4,522 | 6,042 | 6,194 | 9,056 | 87,732 | 427,164 |
|  | **Daily** | 48,499 | 56,540 | 57,775 | 100,313 | 857,988 | 4,283,718 |
|  | **Total** | 53,021 | 62,582 | 63,969 | 109,369 | 945,720 | 4,710,883 |
| **Status quo**** | **Nondaily** | 4,522 | 5,504 | 5,590 | 6,267 | 84,829 | 348,151 |
|  | **Daily** | 48,499 | 51,008 | 51,624 | 68,245 | 826,975 | 3,431,647 |
|  | **Total** | 53,021 | 56,512 | 57,214 | 74,512 | 911,804 | 3,779,797 |
| **Lives saved†** |  |  |  |  |  |  |  |
| **Status quo** | **Nondaily** | - | 538 | 604 | 2,789 | 2,903 | 79,014 |
|  | **Daily** | - | 5,532 | 6,151 | 32,068 | 31,013 | 852,072 |
|  | **Total** | - | 6,070 | 6,755 | 34,857 | 33,916 | 931,085 |
| **Cigarette price** | **Nondaily** | - | 263 | 284 | 1,460 | 1,408 | 37,037 |
|  | **Daily** | - | 2,653 | 2,855 | 18,070 | 14,699 | 429,608 |
|  | **Total** | - | 2,915 | 3,139 | 19,530 | 16,106 | 466,646 |
| **Smoke-free air laws** | **Nondaily** | - | 57 | 78 | 293 | 309 | 9,146 |
|  | **Daily** | - | 611 | 811 | 3,258 | 3,424 | 96,062 |
|  | **Total** | - | 669 | 889 | 3,551 | 3,734 | 105,208 |
| **Media campaigns** | **Nondaily** | - | 40 | 39 | 118 | 254 | 3,966 |
|  | **Daily** | - | 406 | 396 | 1,317 | 2,736 | 41,443 |
|  | **Total** | - | 446 | 435 | 1,435 | 2,990 | 45,408 |
| **Cessation treatments** | **Nondaily** | - | 56 | 69 | 363 | 274 | 10,917 |
|  | **Daily** | - | 572 | 696 | 3,405 | 2,931 | 101,578 |
|  | **Total** | - | 628 | 766 | 3,768 | 3,204 | 112,495 |
| **Health warnings** | **Nondaily** | - | 89 | 97 | 531 | 459 | 14,418 |
|  | **Daily** | - | 927 | 1,001 | 5,970 | 4,981 | 155,907 |
|  | **Total** | - | 1,016 | 1,098 | 6,501 | 5,440 | 170,325 |
| **Marketing bans** | **Nondaily** | - | 37 | 39 | 168 | 223 | 5,091 |
|  | **Daily** | - | 388 | 409 | 1,979 | 2,442 | 55,471 |
|  | **Total** | - | 425 | 448 | 2,147 | 2,665 | 60,562 |

| **Female** | | | | | | | |
| --- | --- | --- | --- | --- | --- | --- | --- |
| **SADs** | **Type of smokers** | **2002** | **2018** | **2019** | **2060** | **Sum by 2018** | **Sum by 2060** |
| **Counterfactual*** | **Nondaily** | 1,316 | 1,366 | 1,387 | 2,248 | 22,406 | 100,529 |
|  | **Daily** | 14,069 | 15,967 | 16,393 | 24,128 | 243,950 | 1,148,728 |
|  | **Total** | 15,385 | 17,333 | 17,780 | 26,376 | 266,356 | 1,249,257 |
| **Status quo**** | **Nondaily** | 1,316 | 1,247 | 1,255 | 1,526 | 21,752 | 82,108 |
|  | **Daily** | 14,069 | 14,536 | 14,799 | 16,099 | 235,967 | 934,537 |
|  | **Total** | 15,385 | 15,784 | 16,054 | 17,626 | 257,719 | 1,016,646 |
| **Lives saved†** |  |  |  |  |  |  |  |
| **Status quo** | **Nondaily** | - | 119 | 132 | 721 | 654 | 18,421 |
|  | **Daily** | - | 1,431 | 1,594 | 8,029 | 7,983 | 214,190 |
|  | **Total** | - | 1,550 | 1,726 | 8,750 | 8,637 | 232,611 |
| **Cigarette price** | **Nondaily** | - | 59 | 64 | 377 | 331 | 8,574 |
|  | **Daily** | - | 675 | 722 | 4,568 | 3,752 | 104,700 |
|  | **Total** | - | 734 | 786 | 4,944 | 4,083 | 113,274 |
| **Smoke-free air laws** | **Nondaily** | - | 12 | 16 | 77 | 66 | 2,118 |
|  | **Daily** | - | 157 | 211 | 815 | 880 | 24,366 |
|  | **Total** | - | 168 | 227 | 892 | 946 | 26,484 |
| **Media campaigns** | **Nondaily** | - | 8 | 9 | 32 | 54 | 924 |
|  | **Daily** | - | 106 | 101 | 333 | 700 | 10,591 |
|  | **Total** | - | 114 | 110 | 365 | 754 | 11,515 |
| **Cessation treatments** | **Nondaily** | - | 13 | 16 | 103 | 63 | 2,766 |
|  | **Daily** | - | 159 | 195 | 981 | 791 | 29,504 |
|  | **Total** | - | 172 | 210 | 1,084 | 854 | 32,270 |
| **Health warnings** | **Nondaily** | - | 20 | 21 | 140 | 101 | 3,379 |
|  | **Daily** | - | 243 | 265 | 1,443 | 1,291 | 39,172 |
|  | **Total** | - | 263 | 285 | 1,582 | 1,392 | 42,550 |
| **Marketing bans** | **Nondaily** | - | 8 | 8 | 43 | 47 | 1,160 |
|  | **Daily** | - | 98 | 104 | 467 | 621 | 13,435 |
|  | **Total** | - | 106 | 112 | 510 | 668 | 14,596 |

* The counterfactual is determined by keeping the policies at their 2002 levels

** The status quo is obtained incorporating policies implemented between 2002 and 2018

**†** Lives saved are calculated based on the difference in smoking-attributable deaths in the status quo and smoking-attributable deaths with the policy implemented. †No change in policy level since 2002

**Table 2.4 Validation of daily and nondaily smoking prevalence, ages 15-65 by gender, from Mexico *SimSmoke* and various national surveys*, 2002-2016. Scenario 4,with 100-cigarettes screen and half policies effect for nondaily.**

|  | | | **Male** | | | | | **Relative difference** | |
| --- | --- | --- | --- | --- | --- | --- | --- | --- | --- |
| **Daily** | **Age groups** | | **2002** | **2006** | **2011** | **2012** | **2016** | **2002-2011** | **2002-2016** |
|  | 15-65 | SimSmoke | 19.6% | 18.3% | 15.2% | 14.9% | 14.0% | -22.7% | -28.7% |
|  |  | Surveys | 19.8% | 20.6% | 11.4% | 15.0% | 10.5% | -42.4% | -47.0% |
|  |  | 95% CI | 17.7%,22.0% | 19.6%,21.5% | 10.2%,12.7% | 14.2%,15.8% | 9.7%,11.4% |  |  |
|  | 15-24 | SimSmoke | 12.7% | 11.3% | 8.8% | 8.6% | 8.3% | -30.7% | -34.5% |
|  |  | Surveys | 12.8% | 16.2% | 6.4% | 10.8% | 6.5% | -50.0% | -49.2% |
|  |  | 95% CI | 10.0%,16.3% | 14.8%,17.7% | 5.0%,8.1% | 9.7%,12.0% | 5.5%,7.8% |  |  |
|  | 25-44 | SimSmoke | 24.2% | 22.2% | 18.3% | 18.0% | 16.5% | -24.2% | -31.5% |
|  |  | Surveys | 24.7% | 22.3% | 12.7% | 18.5% | 12.0% | -48.6% | -51.4% |
|  |  | 95% CI | 21.3%,28.4% | 21.0%,23.7% | 10.9%,14.6% | 17.3%,19.8% | 10.5%,13.5% |  |  |
|  | 45-65 | SimSmoke | 20.0% | 19.5% | 16.8% | 16.6% | 15.6% | -16.2% | -22.1% |
|  |  | Surveys | 20.0% | 22.8% | 15.2% | 18.4% | 12.6% | -24.0% | -37.0% |
|  |  | 95% CI | 16.4%,24.2% | 21.1%,24.5% | 12.5%,18.4% | 16.9%,20.0% | 11.3%,14.0% |  |  |
| **Nondaily** | **Age groups** | | **2002** | **2006** | **2011** | **2012** | **2016** | **2002-2011** | **2002-2016** |
|  | 15-65 | SimSmoke | 6.8% | 6.4% | 5.6% | 5.5% | 5.2% | -17.3% | -23.6% |
|  |  | Surveys | 6.9% | 9.5% | 10.0% | 10.7% | 12.3% | 44.9% | 78.3% |
|  |  | 95% CI | 5.9%,8.0% | 8.9%,10.2% | 9.0%,11.1% | 10.0%,11.3% | 11.5%,13.2% |  |  |
|  | 15-24 | SimSmoke | 3.6% | 3.4% | 2.9% | 2.9% | 2.8% | -18.7% | -20.9% |
|  |  | Surveys | 3.6% | 10.9% | 9.3% | 8.7% | 11.1% | 158.3% | 208.3% |
|  |  | 95% CI | 2.6%,5.0% | 9.7%,12.2% | 7.6%,11.4% | 7.7%,9.7% | 9.7%,12.6% |  |  |
|  | 25-44 | SimSmoke | 7.6% | 7.1% | 6.3% | 6.2% | 5.8% | -17.7% | -23.6% |
|  |  | Surveys | 7.7% | 10.4% | 12.8% | 14.8% | 14.7% | 66.2% | 90.9% |
|  |  | 95% CI | 6.0%,9.8% | 9.4%,11.5% | 11.1%,14.6% | 13.8%,15.8% | 13.5%,16.1% |  |  |
|  | 45-65 | SimSmoke | 9.6% | 9.0% | 7.5% | 7.2% | 6.5% | -22.1% | -32.2% |
|  |  | Surveys | 9.9% | 6.6% | 6.3% | 8.6% | 10.4% | -36.4% | 5.1% |
|  |  | 95% CI | 7.2%,13.5% | 5.7%,7.8% | 5.0%,8.0% | 7.6%,9.6% | 9.0%,12.1% |  |  |

|  | | | **Female** | | | | | **Relative difference** | |
| --- | --- | --- | --- | --- | --- | --- | --- | --- | --- |
| **Daily** | **Age groups** | | **2002** | **2006** | **2011** | **2012** | **2016** | **2002-2011** | **2002-2016** |
|  | 15-65 | SimSmoke | 6.1% | 5.7% | 4.6% | 4.5% | 4.2% | -24.6% | -32.3% |
|  |  | Surveys | 6.3% | 6.3% | 3.7% | 5.0% | 3.2% | -41.3% | -49.2% |
|  |  | 95% CI | 5.2%,7.6% | 5.7%,6.8% | 3.1%,4.3% | 4.6%,5.4% | 2.8%,3.5% |  |  |
|  | 15-24 | SimSmoke | 5.4% | 4.8% | 3.8% | 3.7% | 3.6% | -29.8% | -34.0% |
|  |  | Surveys | 5.6% | 5.1% | 2.1% | 2.5% | 1.9% | -62.5% | -66.1% |
|  |  | 95% CI | 3.7%,8.4% | 4.2%,6.3% | 1.3%,3.3% | 2.0%,3.1% | 1.4%,2.6% |  |  |
|  | 25-44 | SimSmoke | 6.2% | 5.7% | 4.7% | 4.6% | 4.2% | -24.5% | -31.7% |
|  |  | Surveys | 6.2% | 6.4% | 4.1% | 5.9% | 3.2% | -33.9% | -48.4% |
|  |  | 95% CI | 4.9%,7.9% | 5.8%,7.2% | 3.4%,5.1% | 5.2%,6.7% | 2.7%,3.8% |  |  |
|  | 45-65 | SimSmoke | 7.0% | 6.8% | 5.4% | 5.2% | 4.5% | -22.9% | -34.8% |
|  |  | Surveys | 7.1% | 7.2% | 4.5% | 7.5% | 4.2% | -36.6% | -40.8% |
|  |  | 95% CI | 5.4%,9.3% | 6.2%,8.4% | 3.4%,5.9% | 6.6%,8.5% | 3.6%,4.9% |  |  |
| **Nondaily** | **Age groups** | | **2002** | **2006** | **2011** | **2012** | **2016** | **2002-2011** | **2002-2016** |
|  | 15-65 | SimSmoke | 1.6% | 1.6% | 1.4% | 1.4% | 1.4% | -11.9% | -16.6% |
|  |  | Surveys | 1.7% | 3.5% | 3.4% | 3.4% | 3.6% | 100.0% | 111.8% |
|  |  | 95% CI | 1.2%,2.2% | 3.1%,4.0% | 2.8%,4.1% | 3.1%,3.8% | 3.2%,4.0% |  |  |
|  | 15-24 | SimSmoke | 1.2% | 1.1% | 1.0% | 1.0% | 1.0% | -17.0% | -19.6% |
|  |  | Surveys | 1.2% | 4.3% | 4.1% | 3.2% | 3.5% | 241.7% | 191.7% |
|  |  | 95% CI | 0.7%,2.1% | 3.7%,5.1% | 2.9%,5.8% | 2.6%,4.0% | 2.8%,4.5% |  |  |
|  | 25-44 | SimSmoke | 2.0% | 1.9% | 1.6% | 1.6% | 1.5% | -17.3% | -22.9% |
|  |  | Surveys | 2.0% | 3.8% | 3.3% | 4.0% | 4.4% | 65.0% | 120.0% |
|  |  | 95% CI | 1.3%,3.1% | 3.1%,4.6% | 2.5%,4.2% | 3.4%,4.7% | 3.8%,5.1% |  |  |
|  | 45-65 | SimSmoke | 1.6% | 1.7% | 1.6% | 1.6% | 1.5% | 1.5% | -4.5% |
|  |  | Surveys | 1.4% | 2.2% | 3.0% | 2.8% | 2.3% | 114.3% | 64.3% |
|  |  | 95% CI | 0.9%,2.3% | 1.7%,2.9% | 2.0%,4.3% | 2.3%,3.4% | 1.9%,2.8% |  |  |

*The survey point estimates (indicated by dots) and 95% confidence intervals were reported from various surveys, including the Encuesta Nacional de Adicciones (ENA) in 2002 and 2011, the Encuesta Nacional de Salud y Nutricion (ENSANUT) in 2006 and 2012, and the Encuesta Nacional de Consumo de Drogas, Alcohol y Tabaco (ENCODAT) in 2016.

**Table 3.4 Prevalence by smoking status and gender, from Mexico *SimSmoke* under various scenarios in 2002-2060. Scenario 4 ,with 100-cigarettes screen and half policies effect for nondaily.**

| **Male** | | | | | | | |
| --- | --- | --- | --- | --- | --- | --- | --- |
| **Scenarios** | **Type of smoker** | **2002** | **2018** | **2019** | **2060** | **Relative difference** | |
|  |  |  |  |  |  | **in 2018** | **in 2060** |
| **Counterfactual*** | **Nondaily** | 6.7% | 6.0% | 5.9% | 4.8% | - | - |
|  | **Daily** | 19.2% | 18.0% | 18.0% | 15.7% | - | - |
| **Status quo**** | **Nondaily** | 6.7% | 5.2% | 5.1% | 4.0% | -13.0% | -17.9% |
|  | **Daily** | 19.2% | 13.4% | 13.2% | 10.1% | -25.9% | -35.6% |
| **Individual policy effect** | | | | | | | |
| **Cigarette Price** | **Nondaily** | 6.7% | 5.5% | 5.5% | 4.3% | -6.8% | -10.0% |
|  | **Daily** | 19.2% | 15.4% | 15.3% | 12.3% | -14.6% | -21.7% |
| **Smoke-free air laws** | **Nondaily** | 6.7% | 5.9% | 5.8% | 4.8% | -1.4% | -1.5% |
|  | **Daily** | 19.2% | 17.5% | 17.4% | 15.2% | -2.8% | -3.4% |
| **Media campaigns** | **Nondaily** | 6.7% | 5.9% | 5.9% | 4.8% | -0.6% | -0.6% |
|  | **Daily** | 19.2% | 17.8% | 17.7% | 15.5% | -1.2% | -1.3% |
| **Cessation treatments** | **Nondaily** | 6.7% | 5.9% | 5.8% | 4.8% | -1.1% | -1.5% |
|  | **Daily** | 19.2% | 17.7% | 17.6% | 15.3% | -2.1% | -2.6% |
| **Health warnings** | **Nondaily** | 6.7% | 5.8% | 5.8% | 4.7% | -2.0% | -3.1% |
|  | **Daily** | 19.2% | 17.3% | 17.2% | 14.7% | -4.2% | -6.7% |
| **Marketing bans** | **Nondaily** | 6.7% | 5.9% | 5.9% | 4.8% | -0.8% | -1.0% |
|  | **Daily** | 19.2% | 17.7% | 17.6% | 15.4% | -1.7% | -2.3% |
| **Female** | | | | | | | |
| **Scenarios** | **Type of smoker** | **2002** | **2018** | **2019** | **2060** | **Relative difference** | |
|  |  |  |  |  |  | **in 2018** | **in 2060** |
| **Counterfactual*** | **Nondaily** | 1.7% | 1.5% | 1.5% | 1.3% | - | - |
|  | **Daily** | 5.9% | 5.4% | 5.4% | 4.2% | - | - |
| **Status quo**** | **Nondaily** | 1.7% | 1.3% | 1.3% | 1.0% | -14.1% | -20.3% |
|  | **Daily** | 5.9% | 4.0% | 3.9% | 2.6% | -26.6% | -37.6% |
| **Individual policy effect** | | | | | | | |
| **Cigarette Price** | **Nondaily** | 1.7% | 1.4% | 1.4% | 1.1% | -7.7% | -11.7% |
|  | **Daily** | 5.9% | 4.6% | 4.5% | 3.2% | -15.4% | -24.0% |
| **Smoke-free air laws** | **Nondaily** | 1.7% | 1.5% | 1.5% | 1.2% | -1.4% | -1.8% |
|  | **Daily** | 5.9% | 5.3% | 5.2% | 4.1% | -2.8% | -3.5% |
| **Media campaigns** | **Nondaily** | 1.7% | 1.5% | 1.5% | 1.3% | -0.6% | -0.7% |
|  | **Daily** | 5.9% | 5.4% | 5.3% | 4.2% | -1.2% | -1.4% |
| **Cessation treatments** | **Nondaily** | 1.7% | 1.5% | 1.5% | 1.2% | -1.2% | -1.7% |
|  | **Daily** | 5.9% | 5.3% | 5.3% | 4.1% | -2.1% | -2.7% |
| **Health warnings** | **Nondaily** | 1.7% | 1.5% | 1.5% | 1.2% | -2.1% | -3.5% |
|  | **Daily** | 5.9% | 5.2% | 5.1% | 4.0% | -4.1% | -6.7% |
| **Marketing bans** | **Nondaily** | 1.7% | 1.5% | 1.5% | 1.2% | -0.8% | -1.1% |
|  | **Daily** | 5.9% | 5.3% | 5.3% | 4.1% | -1.7% | -2.3% |

* The counterfactual is determined by keeping the policies at their 2002 levels

** The status quo is obtained incorporating policies implemented between 2002 and 2018**Table 4.4 Smoking-attributable deaths (SADs) and lives saved† by smoking status and gender, from Mexico *SimSmoke* under various scenarios in 2002-2060. Scenario 4, with 100-cigarettes screen and half policies effect for nondaily.**

| **Male** | | | | | | | |
| --- | --- | --- | --- | --- | --- | --- | --- |
| **SADs** | **Type of smokers** | **2002** | **2018** | **2019** | **2060** | **Sum by 2018** | **Sum by 2060** |
| **Counterfactual*** | **Nondaily** | 4,522 | 6,043 | 6,195 | 9,059 | 87,736 | 427,258 |
|  | **Daily** | 48,499 | 56,540 | 57,775 | 100,313 | 857,988 | 4,283,709 |
|  | **Total** | 53,021 | 62,583 | 63,970 | 109,372 | 945,724 | 4,710,968 |
| **Status quo**** | **Nondaily** | 4,522 | 5,761 | 5,879 | 7,604 | 86,228 | 385,889 |
|  | **Daily** | 48,499 | 51,000 | 51,616 | 68,082 | 826,937 | 3,428,766 |
|  | **Total** | 53,021 | 56,761 | 57,494 | 75,686 | 913,164 | 3,814,655 |
| **Lives saved†** |  |  |  |  |  |  |  |
| **Status quo** | **Nondaily** | - | 282 | 316 | 1,456 | 1,509 | 41,369 |
|  | **Daily** | - | 5,540 | 6,159 | 32,230 | 31,051 | 854,944 |
|  | **Total** | - | 5,821 | 6,476 | 33,686 | 32,560 | 896,313 |
| **Cigarette price** | **Nondaily** | - | 135 | 146 | 718 | 719 | 18,575 |
|  | **Daily** | - | 2,652 | 2,855 | 18,228 | 14,692 | 432,041 |
|  | **Total** | - | 2,787 | 3,000 | 18,945 | 15,411 | 450,616 |
| **Smoke-free air laws** | **Nondaily** | - | 29 | 39 | 140 | 155 | 4,501 |
|  | **Daily** | - | 611 | 811 | 3,278 | 3,424 | 96,358 |
|  | **Total** | - | 640 | 850 | 3,419 | 3,579 | 100,860 |
| **Media campaigns** | **Nondaily** | - | 20 | 20 | 56 | 127 | 1,934 |
|  | **Daily** | - | 406 | 396 | 1,327 | 2,737 | 41,605 |
|  | **Total** | - | 426 | 416 | 1,383 | 2,864 | 43,539 |
| **Cessation treatments** | **Nondaily** | - | 28 | 35 | 185 | 137 | 5,532 |
|  | **Daily** | - | 577 | 702 | 3,418 | 2,964 | 102,062 |
|  | **Total** | - | 606 | 737 | 3,602 | 3,101 | 107,594 |
| **Health warnings** | **Nondaily** | - | 45 | 49 | 258 | 230 | 7,144 |
|  | **Daily** | - | 931 | 1,005 | 6,013 | 4,997 | 156,687 |
|  | **Total** | - | 975 | 1,054 | 6,271 | 5,227 | 163,831 |
| **Marketing bans** | **Nondaily** | - | 18 | 19 | 79 | 112 | 2,474 |
|  | **Daily** | - | 388 | 409 | 1,995 | 2,440 | 55,693 |
|  | **Total** | - | 406 | 428 | 2,074 | 2,552 | 58,167 |

| **Female** | | | | | | | |
| --- | --- | --- | --- | --- | --- | --- | --- |
| **SADs** | **Type of smokers** | **2002** | **2018** | **2019** | **2060** | **Sum by 2018** | **Sum by 2060** |
| **Counterfactual*** | **Nondaily** | 1,316 | 1,366 | 1,387 | 2,249 | 22,407 | 100,552 |
|  | **Daily** | 14,069 | 15,967 | 16,393 | 24,128 | 243,949 | 1,148,727 |
|  | **Total** | 15,385 | 17,333 | 17,780 | 26,377 | 266,356 | 1,249,279 |
| **Status quo**** | **Nondaily** | 1,316 | 1,304 | 1,317 | 1,855 | 22,066 | 90,662 |
|  | **Daily** | 14,069 | 14,535 | 14,797 | 16,102 | 235,956 | 934,515 |
|  | **Total** | 15,385 | 15,838 | 16,115 | 17,957 | 258,022 | 1,025,176 |
| **Lives saved†** |  |  |  |  |  |  |  |
| **Status quo** | **Nondaily** | - | 62 | 69 | 394 | 341 | 9,890 |
|  | **Daily** | - | 1,433 | 1,596 | 8,026 | 7,993 | 214,212 |
|  | **Total** | - | 1,495 | 1,665 | 8,420 | 8,334 | 224,102 |
| **Cigarette price** | **Nondaily** | - | 31 | 33 | 197 | 170 | 4,455 |
|  | **Daily** | - | 674 | 721 | 4,568 | 3,748 | 104,695 |
|  | **Total** | - | 705 | 754 | 4,765 | 3,918 | 109,150 |
| **Smoke-free air laws** | **Nondaily** | - | 6 | 8 | 38 | 33 | 1,062 |
|  | **Daily** | - | 157 | 211 | 814 | 880 | 24,350 |
|  | **Total** | - | 163 | 219 | 853 | 913 | 25,412 |
| **Media campaigns** | **Nondaily** | - | 4 | 4 | 16 | 27 | 460 |
|  | **Daily** | - | 106 | 101 | 333 | 700 | 10,584 |
|  | **Total** | - | 110 | 106 | 349 | 727 | 11,045 |
| **Cessation treatments** | **Nondaily** | - | 6 | 8 | 53 | 32 | 1,408 |
|  | **Daily** | - | 161 | 197 | 983 | 801 | 29,604 |
|  | **Total** | - | 167 | 204 | 1,036 | 833 | 31,011 |
| **Health warnings** | **Nondaily** | - | 10 | 10 | 70 | 50 | 1,707 |
|  | **Daily** | - | 244 | 266 | 1,444 | 1,296 | 39,220 |
|  | **Total** | - | 254 | 276 | 1,514 | 1,346 | 40,927 |
| **Marketing bans** | **Nondaily** | - | 4 | 4 | 21 | 24 | 578 |
|  | **Daily** | - | 98 | 104 | 466 | 620 | 13,422 |
|  | **Total** | - | 102 | 108 | 488 | 644 | 14,001 |

* The counterfactual is determined by keeping the policies at their 2002 levels

** The status quo is obtained incorporating policies implemented between 2002 and 2018

**†** Lives saved are calculated based on the difference in smoking-attributable deaths in the status quo and smoking-attributable deaths with the policy implemented. †No change in policy level since 2002

**SCENARIO 1 (same policy effects of daily and nondaily smokers without 100-cigarettes screen)**

**Figure 6.1 Validation of Daily Smoking Prevalence, Ages 15-65 by gender, from *Mexico* *SimSmoke* and Various National Surveys*, 2002-2016**

* The survey point estimates (indicated by dots) and 95% confidence intervals were reported from various surveys, including the National Addictions Survey (ENA) in 2002 and 2011, the Global Adult Tobacco Survey (GATS) in 2009 and 2015, the National Health and Nutrition Survey (ENSANUT) in 2012, and the National Survey of Drugs, Alcohol, and Tobacco Consumption (ENCODAT) in 2016.

**Figure 7.1 Validation of Nondaily Smoking Prevalence, Ages 15-65 by gender, from *Mexico* *SimSmoke* and Various National Surveys*, 2002-2016**

* The survey point estimates (indicated by dots) and 95% confidence intervals were reported from various surveys, including the National Addictions Survey (ENA) in 2002 and 2011, the Global Adult Tobacco Survey (GATS) in 2009 and 2015, the National Health and Nutrition Survey (ENSANUT) in 2012, and the National Survey of Drugs, Alcohol, and Tobacco Consumption (ENCODAT) in 2016.

**SCENARIO 2 (half policy effects of ED for SD smokers without 100-cigarettes screen)**

**Figure 6.2 Validation of Daily Smoking Prevalence, Ages 15-65 by gender, from *Mexico* *SimSmoke* and Various National Surveys*, 2002-2016**

* The survey point estimates (indicated by dots) and 95% confidence intervals were reported from various surveys, including the National Addictions Survey (ENA) in 2002 and 2011, the Global Adult Tobacco Survey (GATS) in 2009 and 2015, the National Health and Nutrition Survey (ENSANUT) in 2012, and the National Survey of Drugs, Alcohol, and Tobacco Consumption (ENCODAT) in 2016

**Figure 7.2 Validation of Nondaily Smoking Prevalence, Ages 15-65 by gender, from *Mexico* *SimSmoke* and Various National Surveys*, 2002-2016**

* The survey point estimates (indicated by dots) and 95% confidence intervals were reported from various surveys, including the National Addictions Survey (ENA) in 2002 and 2011, the Global Adult Tobacco Survey (GATS) in 2009 and 2015, the National Health and Nutrition Survey (ENSANUT) in 2012, and the National Survey of Drugs, Alcohol, and Tobacco Consumption (ENCODAT) in 2016.

**SCENARIO 3 (same policy effects of ED and SD smokers considering 100-cigarettes screen)**

**Figure 6.3 Validation of Daily Smoking Prevalence, Ages 15-65 by gender, from *Mexico* *SimSmoke* and Various National Surveys*, 2002-2016**

*The survey point estimates (indicated by dots) and 95% confidence intervals were reported from various surveys, including the National Addictions Survey (ENA) in 2002 and 2011, the Global Adult Tobacco Survey (GATS) in 2009 and 2015, the National Health and Nutrition Survey (ENSANUT) in 2012, and the National Survey of Drugs, Alcohol, and Tobacco Consumption (ENCODAT) in 2016

**Figure 7.3 Validation of Nondaily Smoking Prevalence, Ages 15-65 by gender, from *Mexico* *SimSmoke* and Various National Surveys*, 2002-2016**

* The survey point estimates (indicated by dots) and 95% confidence intervals were reported from various surveys, including the National Addictions Survey (ENA) in 2002 and 2011, the Global Adult Tobacco Survey (GATS) in 2009 and 2015, the National Health and Nutrition Survey (ENSANUT) in 2012, and the National Survey of Drugs, Alcohol, and Tobacco Consumption (ENCODAT) in 2016

**SCENARIO 4 (half policy effects of ED for SD smokers considering 100-cigarettes screen)**

**Figure 6.4 Validation of Daily Smoking Prevalence, Ages 15-65 by gender, from *Mexico* *SimSmoke* and Various National Surveys*, 2002-2016**

* The survey point estimates (indicated by dots) and 95% confidence intervals were reported from various surveys, including the National Addictions Survey (ENA) in 2002 and 2011, the Global Adult Tobacco Survey (GATS) in 2009 and 2015, the National Health and Nutrition Survey (ENSANUT) in 2012, and the National Survey of Drugs, Alcohol, and Tobacco Consumption (ENCODAT) in 2016.

**Figure 7.4 Validation of Nondaily Smoking Prevalence, Ages 15-65 by gender, from *Mexico* *SimSmoke* and Various National Surveys*, 2002-2016**

* The survey point estimates (indicated by dots) and 95% confidence intervals were reported from various surveys, including the National Addictions Survey (ENA) in 2002 and 2011, the Global Adult Tobacco Survey (GATS) in 2009 and 2015, the National Health and Nutrition Survey (ENSANUT) in 2012, and the National Survey of Drugs, Alcohol, and Tobacco Consumption (ENCODAT) in 2016

**Appendix 1.** **Mathematical Formulation of the Revised *Mexico SimSmoke***

*SimSmoke* begins with the population in a baseline year divided into current, former, and never smokers. Assuming a discrete, first-order Markov process, population evolves over time through births and deaths, and the smoking population evolves through initiation, cessation, and relapse.

**Demographics Model**

*SimSmoke* is built first on a demographic model. The total population (*Pop)* is distinguished by time period **t** and age **a** (and is further distinguished in the model by gender)**.** Mortality rates (*MortRate)* are distinguished by age and gender and are based on 2009 mortality rates. Newborns depend on first year deaths rates and fertility rates (*Fert)* of females (**f)** by age with equal birth rates for males and females. Births through the first year (age 0) for each gender are:

*Pop*_t,0_ = 0.5*(1-*MortRate*_0_)* Σ^a^ (*Pop*_t,a,f_ * *Fert*_a,t_), where t=1,…,n; a=14,…,49.

After the first year, the population evolves as:

*Pop*_t,a_ = *Pop*_t-1,a-1_ * (1 – *MortRate*_a-1_). a=1,...,85

**Smoking Model**

*SimSmoke* divides the population in the base year into (1) never smokers (**ns)**, (2) smokers (**s)**, and (3) 17 categories of ex-smokers (n=<1, 1, 2, 3,…, 15, 15+ years) corresponding to years since last time smoking. After the base year, individuals are classified as never smokers from birth until they initiate smoking or die, as shown by:

*Neversmokers_t,a_ = Neversmokers*_t-1,a-1_ * (1 – *MortRate_a-1,ns_*)**(1-Initiation rate_a-1_).*

From never smokers, individuals can become smokers through initiation. Once smokers, they may leave smoking through cessation and return to smoking through relapse. The number of current smokers is tracked as:

*Smokers_t,a_ = Neversmokers*_t-1,a-1_*(1 – *MortRate_a-1,ns_*)**Initiation rate_t,a_*

*+ Smokers_t-1,a-1_*(1 – MortRate_t.a,s_)*(1-Cessation rate_t,a_)* +

*+ Σ^16^_n=1_ Ex-smokers_t-1,a-1,n_*(1 – MortRate_,a-1,n_)*(Relapse rate_a,n_).*

First year former smokers are determined by the first year cessation rate applied to surviving smokers in the previous year. After the first year quit, individuals who have been former smokers for n= 2,…, 15 are defined as:

*Former smokers_t,a,n =_ Former smokers_t-1,a-1,n-1_*(1 – MortRate_a-1,n-1_)*(1-Relapse rate_a-1,n-1_).*

For those who have ceased smoking for more than fifteen years, we add the former smokers from the previous year who have quit for more than fifteen years and who have not died or relapsed in the previous year.

Because of the large prevalence of former smokers at early ages, we specifically modelled the initial former smoker rate. To estimate the number of initial former daily smokers population for all quit years in future years, we assumed that the ratio of age 30 former daily smokers’ prevalence and current daily smokers’ prevalence from 2003 onwards was the same as the former-current daily smoking ratio for 2002 as below:

(Prev-D-Fmr_a=30,t,qt,g_ / Prev-D-Curr_a=30,t,g_) = (Prev-D-Fmr_a=30,t=2002,qt,g_ / Prev-D-Curr_a=30,t=2002,g_),

where Prev-D-Fmr_a=30,t,qt,g_ and Prev-D-Curr_a=30,t,qt,g_ are the former and current daily smoking prevalence at age *a*, year *t*, quit time *qt*, and of gender *g*.

We transformed the above equation into the number of individuals by multiplying the total projected population (Popn) (i.e., never, former and current smokers) by the prevalence of daily former and current smokers for their corresponding years (2002 and year t):

(Popn _a=30,t,g_ * Prev-D-Fmr_a=30,t,qt,g_) / (Popn _a=30,t,g_ * Prev-D-Curr_a=30,t,g_)

= ((Popn _a=30,t=2002,g_ * Prev-D-Fmr_a=30,t=2002,qt,g_) / (Popn _a=30,t=2002,g_ * Prev-D-Curr_a=30,t=2002,g_)),

which equals:

(D-Fmr_a=30,t,qt,g_ / D-Curr_a=30,t,g_) = (D-Fmr_a=30,t=2002,qt,g_ / D-Curr_a=30,t=2002,g_),

where D-Fmr_a=30,t,qt,g_ and D-Fmr_a=30,t,qt,g_ are the number of current daily smokers at age *a*, year *t*, quit time *qt*, and of gender *g*. Therefore, the number of age 30 former daily smokers who quit *qt* year at year *t* was estimated as:

D-Fmr_a=30,t,qt,g_ = (D-Fmr_a=30,t=2002,qt,g_ / D-Curr_a=30,t=2002,g_) * D-Curr_a=30,t,g,_

and the total number of former daily smokers at age 30, regardless of quit years, is:

∑_qt in <1, 1,…, 16+_ D-Fmr_a=30,t,qt,g_.

We used the same method to estimate the initial number of former nondaily smokers population for each quit-year (ND-Fmr_a=30,t,qt,g_).

When the initial former smokers were modeled from age 30 in future years, an equivalent number of former daily and nondaily smokers at age 30 in the same year and by the same gender is subtracted from the number of never smokers who survived from the previous year. Therefore, the projected number of never smokers at age a, time t, of gender after subtraction was estimated as:

Never_a,t,g_ = Never_a-1,t-1,g_ * (1-Mortality rate_a-1,t-1,g,ns_)

- (∑_qt in <1, 1,…, 16+_ D-Fmr_a=30,t,qt,g_ + ∑_qt in <1, 1,…, 16+_ ND-Fmr_a=30,t,qt,g_).

**Smoking Model Assumptions***:*

Cessation

- Cessation rates vary by age and gender
- Quitting behavior only depends on current cessation rates and do not depend on past cessation behaviors
- Cessation rates allow for the transition from current to former smokers and are assumed constant over time subject to changes in policy. They do not depend on past cessation rates (Markov assumption) or vary by socio demographic factors.
- Cessation rates are applied from age 28, based on the assumption that cessation before that age does not affect future health.

Initiation

- Initiation rates vary by age and gender
- Initiation rates vary by age and gender initiation rates are assumed constant over time in the absence of changes in policy. They do not depend on past initiation rates.
- Initiation rates are measured by changes in baseline prevalence from year to year; they incorporate initiation minus cessation, thus reflecting net initiation.

.

Relapse

- Former smokers (FS) and relapse rates are distinguished by age, gender and years quit. As such, relapse rates vary by age and gender and years quit.
- Relapse rates are assumed constant over time, independent of changes in policy. They do not depend on past cessation behaviors.

**Smoking-Attributable Death Model**

Using standard attribution formulas (Shultz et al. 2000), smoking-attributable deaths are estimated for each age and smoking group by multiplying the number of smokers in that group by the difference between the death rate of that smoking group and the death rate of never smokers. To estimate the age and smoking group specific death rate *DR_a,s_*, we used the age and gender specific prevalence (*Prev*), relative risks (*RR_a,s_*) and death rates (*DR*_a_). The death rate of an age group can be expressed as:

*DR*_a_ = *PrevNeversmokers*_a,ns_**DR*_a,ns +_ *PrevSmokers*_a,s_**DR* _a,s_ + Σ^n^ (*PrevEx-smokers*_a,n_ **DR* _a,n_),

Dividing both sides by *DR*_a,ns_, we obtain:

*DR*_a_ /*DR*_a,ns_ = *PrevNeversmokers*_a,ns +_ *PrevSmokers*_a,s_**RR* _a,s_ + Σ^n^ (*PrevEx-smokers*_a,n_ **RR* _a,n_),

because *RR_a,s_= DR_a,s_/DR_a,ns_* and similarly for ex-smokers, and *RR _a,ns_*=1. Rearranging terms, the death rate for never smokers becomes:

*DR_a,ns_ = DR*_a_ / [*PrevNeversmokers*_a,ns_ + *PrevSmokers*_a,s_**RR* _a,s_ + Σ^n^ (*PrevEx-smokers*_a,n_**RR* _a,n_].

For any smoking group s^*^ (of either smokers or ex-smokers), we multiply both sides by *DR_a,s*_ / DR_a,ns_* to obtain the death rate:

*DR_a,s*_ = DR_a_* RR _a.s_*_*_ /

[*PrevNeversmokers_a,ns_+PrevSmokers_a,s_*RR _a,s_+Σ^n^ (PrevEx-smokers_a,n_*RR _a,n_)*].

**Smoking-Attributable Death Model Assumptions:**

- Current, former or never smoker are derived using a formula that depends on the relative risks of total mortality of current smoking and of former smoking (distinguished by years quit) and smoking prevalence rates in 2002. The relative risks are based on the CPS-II, and hence the risks and biases inherent in those measures.
- The relative risks assumed to be constant over time.

**Policy Effects**

The effects of policies are calculated as percent reductions, PR, relative to the initial rates of smoking, initiation and cessation, i.e., [PR= (post-policy rate - initial rate)/initial rate, where PR< 0]. Policies generally have the greatest effect in the first years. The effects are modeled as a permanent additive effect on smoking prevalence in the first year that the policy is implemented, i.e., *Smokers_t,a_ * (1+PR_i,t,a_)* for policy **i** at time period **t** and which may vary by age **a**.

After the first year, policies affect initiation and cessation rates. If the policy affects initiation, the effects of the policy are sustained through lower initiation rates. Throughout the years in which the policy **i** is in effect, the percentage reduction lowers the initiation rate by *(1+PR_i,a_).* The effects of a policy **i** may also be augmented over the same time period through increases in the first year cessation rate by *(1-PR_i,a_).* First-year quit rates remain elevated for each of the policies (except youth access policies), as justified by the higher propensity to quit among individuals who smoke less ([113-115](#_ENREF_113)) as a result of policies and other factors (e.g., economic and informational) creating incentives to quit. It is assumed that the proportion of individuals who relapse increases in direct proportion with any added cessation, implying that the rates of relapse are unaffected by policy changes. Thus, policies have their greatest effect on cessation (directly through the prevalence rate) in the first year that the policy is in effect. Each of the policies also continues to affect initiation and first-year cessation rates during the period over which a policy is in effect.

When more than one policy is in effect, there may be synergies built into the model as described below. Otherwise, there are constant proportional reductions, i.e., *(1+PR_i_)*(1+PR_j_)* for policies **i** and **j,** unless otherwise specified. This formulation implies that the relative effect of a policy is independent of other policies in effect, but the absolute reduction is smaller when another policy is in effect (due to the reduction in the smoking rate from the other policy).

**Taxation Policies**

The tax policy module in *SimSmoke* follows the MPOWER Report and specifies the tax in percentage terms relative to the retail price. In keeping with the recent recommendations under MPOWER, we consider the effect of increasing cigarette excise taxes to 70% of the retail price (Earlier WHO recommended taxes at 75% of price, but included the non-cigarette specific value added tax).

The taxation effect in *SimSmoke* works through price. In *SimSmoke*, the effect of price depends on price elasticities (obtained from relevant studies), which are assumed to be constant in *SimSmoke*. The formula for constant price elasticity, *E*, is defined in terms of the price, P, and quantity, Q, both distinguished by their initial level, designated by subscript t, and their new level, designated by subscript t+1. We use the arc elasticity formula, written as:

*E = [(Q_t+1_ – Q_t_ )/(Q_t+1_ + Q_t_)]/[ (P_t+1_– P_t_)/(P_t+1_ + P_t_)],*  *E < 0.*

To solve for *Q_t_*, the equation is rewritten as:

*(Q_t+1_ – Q_t_ )/(Q_t+1_ + Q_t_) =E*[ (P_t+1_– P_t_)/(P_t+1_ + P_t_)]*

Denoting *E* [ (P_t+1_– P_t_)/(P_t+1_ + P_t_)] by Δ,* the equation can be solved for

*Q_t+1_ – Q_t_ = Δ( Q_t+1_ + Q_t_),* or *Q_t+1_ = Q_t_ *(1 + Δ)/(1 - Δ).*

Since we focus on participation rates (smoking prevalence), Q translates to the number of smokers are Δ_t_ denote the effects of a price change between periods t and t+1. Let *δ_t_ = (1+Δ_t_)/(1-Δ_t_),* then *Q_t+1_ = δ_t_ * Q_t._*

The elasticities usually in terms of overall elasticity (participation and conditional quantity), depending on the type of elasticity available. For example, the average overall elasticity for the US is 0.4. We use prevalence elasticities that vary by age and, based on studies from Mexico, are set equal to those in the US.

Actual cigarette prices adjusted for inflation are used for the tracking period. Future price changes occur through tax increases, which are specified as a portion of price. Future inflation adjusted prices are assumed constant in the absence of a tax change.

The model assumes that prices increase in absolute terms with the amount of the cigarette tax, based on Sung ([116](#_ENREF_116)) and evidence from other countries ([117](#_ENREF_117)). Let *T_t_* be the tax rate as expressed as a percent of price in period **t**. To derive future prices, we first estimate the price net of taxes, PNT, as the retail price, P_t,_ multiplied by (1-*T*) for the last year before projection. Assuming that the factory price (after adjusting for inflation), the import and value added tax and the average percent markup by the manufacturers and foreign tobacco firms remains constant, then the amount of the total tax will be equal to *T*/ (1-*T*) times the price net of taxes ([118](#_ENREF_118)). Then the retail price can be re-written as: P_t_ = PNT *+* PNT **T_t_ /(1-T_t_ )*, where the second term is the amount of price that is taxed. We assume that the net of tax price remains constant and that prices increase by the amount of the tax, so that the new price is obtained by substituting the new tax ***T_t +1_*** for ***T_t_***  in the second term.

Taxes on cigarettes may include value added, import and excise taxes. We do not consider import taxes in our analysis, since they are not implemented in Mexico. Excise taxes apply only to cigarettes and may be implemented in percentage terms, i.e., ad valorem taxes, or fixed amounts per unit, i.e. specific taxes. Mexico has traditionally had an ad valorem tax, but has added specific tax since 2010. In our analysis, the value added tax (in percentage terms) is not considered a tax on cigarettes, since it applies to all goods, not just cigarettes. However, it is applied to excise taxes as well as the price net of taxes, and, consequently, it amplifies the effect of an excise tax increase, so that the excise tax as a percent of price diminishes when price adjusts to a new level. Consequently, we consider two cases: 1) where the excise tax is increased to 70%, but after adjustment of the value added tax no longer equals 70%, and 2) where the excise tax is further adjusted, after allowing for the effect of the value added tax, so that it equals 70% of the final retail price. When the effect of the value added tax is incorporated, the value added tax is included in **T_t_** in calculating the price net of tax and amount of the tax. To exclude the amplifying effect of total taxes, we only consider excise taxes in **T_t_** in calculating both the price net of tax and amount of the tax.

**Smoke-Free Air Laws**

Three types of smoke-free air policies (worksite, restaurant, and bars) are included in MPOWER *SimSmoke*, with the effect of worksite further distinguished by its stringency. Worksite bans are distinguished corresponding to data provided in the MPOWER Report as: 1) partial as designated by a ban in 2 of the 3 types of facilities: health, university, and government facilities, 2) ban in indoor offices only, and 3) ban in all indoor workplaces (including offices and other indoor workplaces, universities, and government). These policies are cumulative, i.e., inclusive of the previous policy, with the MPOWER target policy a complete ban. Consistent with the MPOWER Report, the model distinguishes only a total restaurant ban and a total ban in bars and restaurants. The MPOWER model includes a ban in pubs and bars. The model includes bans in other public places, based on the corresponding MPOWER variables, public transport, and supplementary information. An enforcement index is based on the MPOWER Report, whereby enforcement is scored between 1 and 10, with 10 as the highest level. In addition, publicity is directly dependent on the level of tobacco control campaigns.

The effects with a high level of publicity and enforcement are: worksites = 6%, restaurants = 2%, and bars = 1%. The effects are adjusted downward by using the URBAN index to account for the percent of the population not affected. In addition, while half of the effects occur automatically through passage of the law (e.g., due to a change in norms), the other half of the effects depend on enforcement (0<ENF<1, using the MPOWER index=1,…10 divided by 10 to be scaled to 1) and a publicity index based on tobacco control spending (= 0.5 if spending is low or non-existent, 0.75 if spending is moderate, and 1 if spending is high). Letting smoking-free laws *SFL’* equal the effect size of type k with high enforcement and publicity, the effect *SFL_i_* for country i is:

*SFL_i,k_ = SFL_~~k~~_’ * URBAN_i_ * LABOR_i_ *0.5 (1+0.5*ENF_i_+0.5*PUB_i_).*

**Marketing Bans**

Four levels of marketing restriction policies are distinguished: none, minimal, moderate, and comprehensive. The effects differ for prevalence, cessation, and initiation, and depend on enforcement. As for smoke-free laws, a total lack of enforcement reduces the impact by half (0<ENF<1, using the MPOWER index = 1…10 divided by 10 to be scaled to 1). For marketing restrictions at level k with high enforcement designated as MR’, the effect for country i will be:

*MR_i,k_ = MR_k_’ * 0.5 (1+ENF_i_).*

**Health Warnings**

*SimSmoke* distinguishes four levels of policy (none, mild, moderate, and strong). For effect size *HW*’ at level k for health warning, the effect for country i is:

*HW_i,k_ = HW_k_’*

**Tobacco Control Campaigns**

Three levels of a campaign are specified: high, medium, and low. The degree of urbanization affects the ability to reach rural populations through the media and even local campaigns, and is taken into consideration in examining the effect of tobacco control spending. The effects depend on the other policies in effect, due to the added publicity surrounding these policies. For tobacco control spending at level k at effect size *TC,’* the effect for country i is:

*TC_i,k_ = TC_~~k~~_’ *OTHER_POLICIES_i_*

**Cessation Treatment Policy**

The cessation treatment policy module includes pharmacotherapy (PT) availability, treatment coverage, quitlines and health care provider brief interventions. The effects occur through prevalence and cessation rates, and there is no effect on initiation.

The PT sub-policy option corresponds to the information in the MPOWER Report regarding whether nicotine replacement treatment (NRT) and/or Varenecline are available and where they may be obtained. When pharmacotherapy is available, the MPOWER Report distinguishes whether NRT is available in a general store or pharmacy and if a prescription (Rx) is required. We do not distinguish effect sizes (they are each assigned a value 1) except when NRT is only available by prescription. Since access is thus more limited, the NRT variable is multiplied by 0.5, indicating that the effect is reduced by 50%. To get an overall effect, the indicators for NRT and Varenecline are summed and divided by 3 to obtain an overall indicator with a value between 0 and 1 that is used to scale the percentage effect of the new treatment availability sub-policy. If the value of the sub-policy is 1 (the policy is effect in full), then prevalence is reduced by 1.0% in the first year of the policy (which is roughly equivalent to the effect of 15% of smokers using either or both of the PTs with a 10% average success rate net of relapse in the first year) and the pre-policy cessation rate is increased by 6% in all years after the first (equivalent to quit attempts increased by 30% due to new PT users with those users having a 20% first year success rate).

Treatment coverage does not distinguish pharmacotherapy and behavioral therapy, but rather focuses on where the treatment is provided. We followed the MPOWER Report that distinguishes place of provision of cessation treatments by the following: primary care facilities, hospitals, offices of health professionals, community and other. For each location, we designate a value of score for each of the above locations: 0 = None, 1 = Yes in some, and 2 = Yes in most. We then sum the scores. The highest possible score is 10, but a full effect is designated if at least 4 of the 5 places have indicated yes, whereby a score of 8 is for the full effect. To scale to 1, we multiply by 0.125 (1/8). That indicator is used to scale the effect of the financial coverage of treatment sub-policy.

Previously the financial access sub-policy in *SimSmoke* included the level of publicity, which is no longer an option in this module. The level of publicity is now automatically set based on the level of tobacco control campaigns. The effect of publicity on financial coverage of treatment is (1- 0.25 *(1- publicity)); where publicity = 1 if a high level tobacco control campaign, 0.5 if medium level, and 0.25 if low level)) so that the effects of treatment availability are scaled as much as a 25% reduction by this variable if publicity is less than high (equivalent to an additional 15% of smokers using treatment with a 15% success rate net of relapse) in the first year of the policy and the cessation rate is increased by 12% (equivalent to quit attempts increased by 40% with the new treatment users having a 30% first year success rate) in all years after the first.

In the MPOWER Report, quitlines are distinguished only by whether the population has access to a toll free quitline. In previous versions of *SimSmoke*, quitlines were distinguished by TYPE of quitline: passive, active without follow-up or active with follow-up, with corresponding values of 1, 2 and 3. The default value for TYPE 2 (an active quitline without follow-up) is designated if no information is provided. In previous versions of *SimSmoke*, we also distinguished whether there was no cost NRT provided to callers, which is now excluded. The effect of quitlines also depends on publicity, which uses the same equation as for financial access sub-policy.

If an active quitline with follow-up is implemented and the program is well publicized through a tobacco control campaign, then prevalence is reduced by 0.75% (equivalent to 5% of smokers using the quitline with a 15% success rate net of relapse) in the first year of the policy and the cessation rate is increased by 7.5% (quit attempts increased by 25% with users having a 30% first year success rate) in all future years. When the quitline is not active with follow-up, effective-ness is reduced by (1- 0.5*TYPE/3). The MPOWER Report best case is only active quitlines.

Brief interventions are by health care providers and are evaluated based on a scale. The scale reaches 100% when the provider asks each patient about their smoking status, advises current smokers to quit, provides advice on quitting methods and follows up with the patient.

When more than one of the sub-policies is implemented, the effects are additive with the following exceptions. As in previous versions of *SimSmoke*, the effect of quitlines with NRT is reduced by 25% if there is also a policy of complete financial coverage of treatment, because NRT is then made available at no cost through other sources. In addition, a synergistic effect occurs between sub-policies 1-3 (which all provide for more treatment use) and brief interventions, as health care providers encourage treatment uses that have become more readily available. Brief intervention then increases the effect of sub-policies 1, 2 and 3 on prevalence by 10% and on the cessation rate by 30%. With all sub-policies implemented, smoking prevalence is reduced by 4.75% and the first year cessation rate is increased by 39.3%. We allow for less effect if the country is rural because of less access to health care in rural areas, but we allow for a 50% greater effect in countries where awareness of health dangers is low. Thus, with effect size *CTP*’ for cessation treatment policies, the effect in country i is

*CTP_i,k_ = CTP_~~k~~_’ * URBAN_i_*

**Policy Models** **Assumptions:**

- The policy effects are as constant percentage reductions, through prevalence in the first year of implementation, and through initiation and cessation in future years.
- When more than one policy is applied, the effects are assumed to be multiplicative through their respective percentage reductions. An exception is synergies (as described above) through publicity from media campaigns.
- Policies are applied to all smokers with no distinctions by cigarette type smoked or whether there is dual use.
- Policies as implemented are applied to the tracking period (2002 to 2013).

In the absence of policy change, future smoking prevalence and smoking-attributable deaths are projected assuming that policies are assumed to remain constant after 2013 at their levels in 2013.

**Appendix 2. Sensitivity Analysis of the Different Measures of Smoking**

Data on daily (ED) and nondaily (SD) smoking prevalence is available from ENA (2002, 2011), GATS (2009, 2015), ENA/ENCODAT (2016). Those surveys all covered population aged 15-65 and were grouped into three age groups (15-24, 25-44, 45-65). Two measures of ED and SD smokers were available from 2002 and 2011 ENA and from 2016 (ENA/ENCODAT). The first definition is based on “having smoked at least 100 cigarettes in his/her lifetime and having smoked daily/occasionally during the last 30 days” (Def 1), and the second definition is “having smoked daily/occasionally during the last 30 days” (Def 2). The former is generally accepted in high-income nations and the latter is less stringent. The sum of SD and ED smokers according to the two definitions is the value of All smokers under two definitions. The relative difference of the two definitions is defined as the percentage increase between the prevalence by Def 1 and Def 2, i.e., (Def 2- Def 1)/Def 1.

For the 2002 ENA (Table1), the relative differences for ED smokers decreased from 18% for males (20% for females) among ages 15-24 to 4.4% for males (11% for females) among ages 45-65; relative differences for SD smokers decreased from 244% for males (292% for females) among ages 15-24 to 32% for males (47% for females) among ages 45-65; relative differences for all smokers decreased from 67% for males (65% for females) among ages 15-24 to 13% for males (17% for females) among ages 45-65. For ages 15-65, the average prevalence was 9% (13%) higher in relative terms for male (female) daily smokers, but 78% (153%) higher for male (female) nondaily smokers, yielding a difference of 27% (46%) for combined smokers.

**Appendix 2: Table 1: 2002 ENA Daily, Nondaily, and All Current Smoking Prevalence, by Gender and Age, for Two Definitions of Prevalence with Their Relative Differences (RD)**

|  | **Males (2002)** | | | | | | | | | |
| --- | --- | --- | --- | --- | --- | --- | --- | --- | --- | --- |
|  |  | **Daily smokers** | | | **Nondaily smokers** | | | **All smokers** | | |
| **Age** | **N** | Def 1 | Def 2 | RD | Def 1 | Def 2 | RD | Def 1 | Def 2 | RD |
| **15-24 years** | 1,200 | 12.8% | 15.1% | 18.0% | 3.6% | 12.4% | 244.4% | 16.5% | 27.5% | 66.7% |
| **25-44 years** | 2,226 | 24.7% | 26.9% | 8.9% | 7.7% | 11.8% | 53.2% | 32.4% | 38.7% | 19.4% |
| **45-65 years** | 1,193 | 20.0% | 20.9% | 4.4% | 9.8% | 12.9% | 31.6% | 29.9% | 33.8% | 12.9% |
| **Total** | 4,619 | 19.8% | 21.6% | 9.1% | 6.9% | 12.3% | 78.3% | 26.7% | 33.9% | 27.0% |
|  | **Females (2002)** | | | | | | | | | |
|  |  | **Daily smokers** | | | **Nondaily smokers** | | | **All smokers** | | |
| **Age** | **N** | Def 1 | Def 2 | RD | Def 1 | Def 2 | RD | Def 1 | Def 2 | RD |
| **15-24 years** | 1,409 | 5.6% | 6.7% | 19.6% | 1.2% | 4.7% | 291.7% | 6.9% | 11.4% | 65.2% |
| **25-44 years** | 2,919 | 6.2% | 7.0% | 12.9% | 2.0% | 5.2% | 160.0% | 8.3% | 12.3% | 48.2% |
| **45-65 years** | 1,471 | 7.1% | 7.8% | 10.9% | 1.5% | 2.1% | 46.7% | 8.5% | 10.0% | 17.0% |
| **Total** | 5,799 | 6.3% | 7.1% | 12.7% | 1.7% | 4.3% | 152.9% | 7.9% | 11.5% | 45.6% |

Notes: RD= relative difference, Def 1 = with 100 cigarettes lifetime screen, Def 2 = without 100 cigarettes lifetime screen, N= sample size

For the 2011 ENA (Table 2), the relative differences of ED/SD/All smokers decreased across age groups from 15-24 to 45-65 for both genders in most cases. Relative differences for male ED smokers decreased from 3.1% among ages 15-24 to 1.3% among ages 45-65, and for females decreased from 10% among ages 15-24 to 4.9% among ages 25-44, then increased to 8.5% in 45-65 for females; relative differences for SD smokers decreased from 104% among ages 15-24 to 36% among ages 25-44, then slightly increased to 38% among ages 45-65 for males, but it decreased from 100% among ages 15-24 to 62% among ages 45-65 for females; relative differences for all smokers decreased from 63% for males (71% for females) among ages 15-24 to 12% for males (30% for females) among ages 45-65. For ages 15-65, the total male (female) daily smoking prevalence is 1.8% (2.7%) higher in relative terms for Def 2 compared with Def 1, but the total male (female) nondaily smoking prevalence is 56% (79%) higher for Def 2, yielding a difference of 27% (41%) for combined smokers.

**Appendix 2: Table 2: 2011 ENA Daily, Nondaily, and All Current Smoking Prevalence, by Gender and Age, for Two Definitions of Prevalence with Their Relative Differences**

|  | **Males (2011)** | | | | | | | | | |
| --- | --- | --- | --- | --- | --- | --- | --- | --- | --- | --- |
|  |  | **Daily smokers** | | | **Nondaily smokers** | | | **All smokers** | | |
| **Age** | **N** | Def 1 | Def 2 | RD | Def 1 | Def 2 | RD | Def 1 | Def 2 | RD |
| **15-24 years** | 1,921 | 6.4% | 6.6% | 3.1% | 9.3% | 19.0% | 104.3% | 15.7% | 25.6% | 63.1% |
| **25-44 years** | 2,697 | 12.6% | 13.0% | 3.2% | 12.8% | 17.4% | 35.9% | 25.4% | 30.3% | 19.3% |
| **45-65 years** | 1,623 | 15.3% | 15.5% | 1.3% | 6.3% | 8.7% | 38.2% | 21.6% | 24.2% | 12.1% |
| **Total** | 6,241 | 11.4% | 11.6% | 1.8% | 10.0% | 15.6% | 56.0% | 21.4% | 27.2% | 27.1% |
|  | **Females (2011)** | | | | | | | | | |
|  |  | **Daily smokers** | | | **Nondaily smokers** | | | **All smokers** | | |
| **Age** | **N** | Def 1 | Def 2 | RD | Def 1 | Def 2 | RD | Def 1 | Def 2 | RD |
| **15-24 years** | 2,125 | 2.0% | 2.2% | 10.0% | 4.1% | 8.2% | 100.0% | 6.1% | 10.4% | 70.5% |
| **25-44 years** | 3,673 | 4.1% | 4.3% | 4.9% | 3.3% | 5.6% | 69.7% | 7.4% | 9.9% | 33.8% |
| **45-65 years** | 2,259 | 4.5% | 4.9% | 8.5% | 3.0% | 4.9% | 61.5% | 7.5% | 9.8% | 29.8% |
| **Total** | 8,057 | 3.7% | 3.8% | 2.7% | 3.4% | 6.1% | 79.4% | 7.1% | 10.0% | 40.8% |

Notes: RD= relative difference, Def 1 = with 100 cigarettes lifetime screen, Def 2 = without 100 cigarettes lifetime screen, N= sample size

For the 2016 ENA/ENCODAT (Table 3), most relative differences of SD/All smokers decreased across age groups from 15-24 to 45-65 for both genders. Relative differences for SD smokers decreased from 87% for males (100% for females) among ages 15-24 to 27% for males (58% for females) among ages 45-65; relative differences for All smokers decreased from 57% for males (64% for females) among ages 15-24 to 15% for males (27% for females) among ages 45-65; relative differences for ED smokers decreased from 6.2% for males (5.3% for females) among ages 15-24 to 4.2% for males (3.1% for females) among ages 25-44, but increased to 5.3% (10%) among ages 45-65. Therefore, different definitions have large influence on young ED/SD/All smoker prevalence. For ages 15-65, the total male (female) daily smoking prevalence is 4.8% (3.1%) higher in relative terms for Def 2 compared with Def 1, but the total male (female) nondaily smoking prevalence is 50% (67%) higher for Def 2, yielding a difference of 29% (39%) for combined smokers.

**Appendix 2: Table 3: 2016 ENA/ENCODAT Daily, Nondaily, and All Current Smoking Prevalence, by Gender and Age, for Two Definitions of Prevalence with Their Relative Differences (RD)**

|  | **Males (2016)** | | | | | | | | | |
| --- | --- | --- | --- | --- | --- | --- | --- | --- | --- | --- |
|  |  | **Daily smokers** | | | **Nondaily smokers** | | | **All smokers** | | |
| **Age** | **N** | Def 1 | Def 2 | RD | Def 1 | Def 2 | RD | Def 1 | Def 2 | RD |
| **15-24 years** | 6,045 | 6.5% | 6.9% | 6.2% | 11.1% | 20.7% | 86.5% | 17.6% | 27.6% | 56.8% |
| **25-44 years** | 7,958 | 12.0% | 12.5% | 4.2% | 14.7% | 20.7% | 40.8% | 26.7% | 33.1% | 24.0% |
| **45-65 years** | 6,499 | 12.5% | 13.2% | 5.3% | 10.4% | 13.2% | 26.5% | 22.9% | 26.3% | 14.9% |
| **Total** | 20,502 | 10.5% | 11.0% | 4.8% | 12.3% | 18.4% | 49.6% | 22.8% | 29.4% | 28.9% |
|  | **Females (2016)** | | | | | | | | | |
|  |  | **Daily smokers** | | | **Nondaily smokers** | | | **All smokers** | | |
| **Age** | **N** | Def 1 | Def 2 | RD | Def 1 | Def 2 | RD | Def 1 | Def 2 | RD |
| **15-24 years** | 6,890 | 1.9% | 2.0% | 5.3% | 3.5% | 7.0% | 100.0% | 5.5% | 9.0% | 63.6% |
| **25-44 years** | 13,251 | 3.2% | 3.3% | 3.1% | 4.4% | 6.9% | 56.8% | 7.6% | 10.2% | 34.2% |
| **45-65 years** | 9,609 | 4.2% | 4.6% | 10.4% | 2.3% | 3.7% | 57.8% | 6.5% | 8.3% | 27.4% |
| **Total** | 29,750 | 3.2% | 3.3% | 3.1% | 3.6% | 6.0% | 66.7% | 6.7% | 9.3% | 38.8% |

Notes: RD= relative difference, Def 1 = with 100 cigarettes lifetime screen, Def 2 = without 100 cigarettes lifetime screen, N= sample size

In summary, the relative differences are greater for SD than ED smokers at most ages and for both genders. These differences decrease with age from 15-24 to 45-65, and tend to be greater for females than males. i.e., the definitions have larger influence on young than older smoker prevalence. For each year and age group, the prevalence of male smokers is higher than females, but the relative differences for males and females usually follow the same pattern.

**References**

1. Collaborators GBDRF. Global, regional, and national comparative risk assessment of 84 behavioural, environmental and occupational, and metabolic risks or clusters of risks, 1990-2016: a systematic analysis for the Global Burden of Disease Study 2016. Lancet. 2017;390(10100):1345-422.

2. World Health Organization. History of the Who Framework Convention on Tobacco Control. Geneva: World Health Organization; 2009. v, 51 p. p.

3. World Health Organization. WHO Report on the Global Tobacco Epidemic, 2008: The MPOWER package. Geneva: World Health Organization, 2008.

4. Hopkins DP, Briss PA, Ricard CJ, Husten CG, Carande-Kulis VG, Fielding JE, et al. Reviews of evidence regarding interventions to reduce tobacco use and exposure to environmental tobacco smoke. Am J Prev Med. 2001;20(2 Suppl):16-66.

5. U.S. DHHS. Preventing tobacco use among young people: a report of the Surgeon General. Atlanta, GA: Centers for Disease Control and Prevention, National Center for Chronic Disease Prevention and Health Promotion, Office on Smoking and Health, 1994 1994. Report No.

6. U.S. DHHS. Healthy people 2010. Atlanta: Centers for Disease Control, Office of Disease Prevention and Health Promotion; 2000.

7. Levy DT, Tam J, Kuo C, Fong GT, Chaloupka F. The Impact of Implementing Tobacco Control Policies: The 2017 Tobacco Control Policy Scorecard. J Public Health Manag Pract. 2018;24(5):448-57.

8. Taylor DH, Jr., Hasselblad V, Henley SJ, Thun MJ, Sloan FA. Benefits of smoking cessation for longevity. Am J Public Health. 2002;92(6):990-6.

9. U.S. DHHS. The Health Benefits of Smoking Cessation: a report of the Surgeon General. Atlanta Georgia: U.S. Department of Health and Human Services, Public Health Service, Centers for Disease Control, Office on Smoking and Health., 1990.

10. Hu TW, Sung HY, Keeler TE. Reducing cigarette consumption in California: tobacco taxes vs an anti-smoking media campaign. Am J Public Health. 1995;85(9):1218-22.

11. Hu TW, Sung HY, Keeler TE. The state antismoking campaign and the industry response: the effects of advertising on cigarette consumption in California. Am Econ Rev. 1995;85(2):85-90.

12. Farrelly MC, Pechacek TF, Chaloupka FJ. The impact of tobacco control program expenditures on aggregate cigarette sales: 1981-2000. J Health Econ. 2003;22(5):843-59.

13. Homer JB, Hirsch GB. System dynamics modeling for public health: background and opportunities. Am J Public Health. 2006;96(3):452-8.

14. Levy DT, Bauer JE, Lee HR. Simulation modeling and tobacco control: creating more robust public health policies. Am J Public Health. 2006;96(3):494-8.

15. Mendez D, Warner KE, Courant PN. Has smoking cessation ceased? Expected trends in the prevalence of smoking in the United States. Am J Epidemiol. 1998;148(3):249-58.

16. Mendez D, Warner KE. Adult cigarette smoking prevalence: declining as expected (not as desired). Am J Public Health. 2004;94(2):251-2.

17. Tengs TO, Ahmad S, Moore R, Gage E. Federal policy mandating safer cigarettes: a hypothetical simulation of the anticipated population health gains or losses. J Policy Anal Manage. 2004;23(4):857-72.

18. Tengs TO, Osgood ND, Chen LL. The cost-effectiveness of intensive national school-based anti-tobacco education: results from the tobacco policy model. Prev Med. 2001;33(6):558-70.

19. Tengs TO, Osgood ND, Lin TH. Public health impact of changes in smoking behavior: results from the Tobacco Policy Model. Med Care. 2001;39(10):1131-41.

20. Ahmad S. Increasing excise taxes on cigarettes in California: a dynamic simulation of health and economic impacts. Prev Med. 2005;41(1):276-83.

21. Ahmad S, Billimek J. Estimating the health impacts of tobacco harm reduction policies: a simulation modeling approach. Risk Anal. 2005;25(4):801-12.

22. Ahmad S, Billimek J. Limiting youth access to tobacco: Comparing the long-term health impacts of increasing cigarette excise taxes and raising the legal smoking age to 21 in the United States. Health Policy. 2006.

23. Currie LM, Blackman K, Clancy L, Levy DT. The effect of tobacco control policies on smoking prevalence and smoking-attributable deaths in Ireland using the IrelandSS simulation model. Tob Control. 2012. Epub 2012/05/29.

24. Levy D, de Almeida LM, Szklo A. The Brazil SimSmoke policy simulation model: the effect of strong tobacco control policies on smoking prevalence and smoking-attributable deaths in a middle income nation. PLoS Med. 2012;9(11):e1001336. Epub 2012/11/10.

25. Levy D, Gallus S, Blackman K, Carreras G, La Vecchia C, Gorini G. Italy SimSmoke: the effect of tobacco control policies on smoking prevalence and smoking attributable deaths in Italy. BMC Public Health. 2012;12:709.

26. Levy D, Rodriguez-Buno RL, Hu TW, Moran AE. The potential effects of tobacco control in China: projections from the China SimSmoke simulation model. BMJ. 2014;348:g1134. Epub 2014/02/20.

27. Levy DT, Bales. S, Nikolayev L. The Role of Public Policies in Reducing Smoking and Deaths Caused by Smoking in Vietnam: Results from the Vietnam Tobacco Policy Simulation Model. Social Sciences and Medicine. 2006;62(7):1819-30.

28. Levy DT, Blackman K, Currie LM, Mons U. Germany SimSmoke: the effect of tobacco control policies on future smoking prevalence and smoking-attributable deaths in Germany. Nicotine Tob Res. 2013;15(2):465-73.

29. Levy DT, Cho SI, Kim YM, Park S, Suh MK, Kam S. SimSmoke model evaluation of the effect of tobacco control policies in Korea: the unknown success story. Am J Public Health. 2010;100(7):1267-73. Epub 2010/05/15.

30. Levy DT, Meza R, Zhang Y, Holford TR. Gauging the Effect of U.S. Tobacco Control Policies From 1965 Through 2014 Using SimSmoke. Am J Prev Med. 2016;50(4):535-42.

31. Nagelhout GE, Levy DT, Blackman K, Currie L, Clancy L, Willemsen MC. The effect of tobacco control policies on smoking prevalence and smoking-attributable deaths. Findings from the Netherlands SimSmoke Tobacco Control Policy Simulation Model. Addiction. 2012;107(2):407-16.

32. Levy D, Fergus C, Rudov L, McCormick-Ricket I, Carton T. Tobacco Policies in Louisiana: Recommendations for Future Tobacco Control Investment from SimSmoke, a Policy Simulation Model. Prev Sci. 2016;17(2):199-207.

33. Levy D, Tworek C, Hahn E, Davis R. The Kentucky SimSmoke Tobacco Policy Simulation Model: Reaching Healthy People 2010 Goals Through Policy Change. Southern Medical Journal. 2008;101(5):503-7.

34. Levy DT, Boyle RG, Abrams DB. The role of public policies in reducing smoking: the Minnesota SimSmoke tobacco policy model. Am J Prev Med. 2012;43(5 Suppl 3):S179-86. Epub 2012/10/25.

35. Levy DT, Huang AT, Havumaki JS, Meza R. The role of public policies in reducing smoking prevalence: results from the Michigan SimSmoke tobacco policy simulation model. Cancer Causes Control. 2016;27(5):615-25.

36. Levy DT, Nikolayev N, Mumford EA. The Role of Public Policies in Reducing Smoking Prevalence and Deaths Caused by Smoking in California:Results from the California Tobacco Policy Simulation Model. Calverton Maryland: Pacific Institute, 2004.

37. Levy DT, Ross H, Powell L, Bauer JE, Lee HR. The role of public policies in reducing smoking prevalence and deaths caused by smoking in Arizona: results from the Arizona tobacco policy simulation model. J Public Health Manag Pract. 2007;13(1):59-67.

38. Levy DT, Tworek C, Hahn EJ, Davis RE. The Kentucky SimSmoke tobacco policy simulation model: reaching Healthy People 2010 goals through policy change. South Med J. 2008;101(5):503-7.

39. Fleischer NL, Thrasher JF, Reynales-Shigematsu LM, Cummings KM, Meza R, Zhang Y, et al. Mexico SimSmoke: how changes in tobacco control policies would impact smoking prevalence and smoking attributable deaths in Mexico. Glob Public Health. 2016:1-16.

40. Reynales-Shigematsu LM, Fleischer NL, Thrasher JF, Zhang Y, Meza R, Cummings KM, et al. Effects of tobacco control policies on smoking prevalence and tobacco-attributable deaths in Mexico: the SimSmoke model. Rev Panam Salud Publica. 2015;38(4):316-25.

41. México: INSP/PanAmerican Health Organization. Global Adult Tobacco Survey, México 2015. Cuernavaca: México: INSP/PanAmerican Health Organization, 2017.

42. Instituto Nacional de Psiquiatría Ramón de la Fuente Muñiz; Instituto Nacional de Salud Pública CNClA, Secretaría de Salud. . Encuesta Nacional de Consumo de Drogas, Alcohol y Tabaco 2016-2017: Reporte de Tabaco. Reynales- Shigematsu LM. Zavala-Arciniega L, Paz-Ballesteros WC, Gutiérrez-Torres DS, García-Buendía JC, Rodriguez-Andrade MA, Gutiérrez-Reyes, J., Franco-Núñez, A., Romero-Martínez, M. y Mendoza-Alvarado, L. Ciudad de México, México: INPRFM, 2017.

43. Levy DT, Nikolayev N, Mumford EA. Recent Trends in Smoking and the Role of Public Policies: Results from the SimSmoke Tobacco Control Policy Simulation Model. Addiction. 2005;10(10):1526-37.

44. Levy DT, Nikolayev N, Mumford EA. The Healthy People 2010 Smoking Prevalence and Tobacco Control Objectives: Results from the SimSmoke Tobacco Control Policy Simulation Model. Cancer Causes and Control. 2005;16(4):359-71.

45. Waters H, Saenz de Miera B, Ross H, Reynales Shigematsu LM. The economics of tobacco and tobacco taxation in Mexico. . Paris, France: International Union Against Tuberculosis and Lung Disease. : 2010.

46. Shultz JM, Novotny TE, Rice DP. Quantifying the disease impact of cigarette smoking with SAMMEC II software. Public Health Rep. 1991;106(3):326-33.

47. CDC. Cigarette smoking among adults--United States, 1998. MMWR Morb Mortal Wkly Rep. 2000;49(39):881-4.

48. CONAPO. Consejo Nacional de Populacion. Mexico City, Mexico2019 [cited 2019 March 15]; Available from: [www.conapo.gob.mx](http://www.conapo.gob.mx).

49. Kuri-Morales PA, Gonzalez-Roldan JF, Hoy MJ, Cortes-Ramirez M. [Epidemiology of tobacco use in Mexico]. Salud Publica Mex. 2006;48 Suppl 1:S91-8. Epub 2007/08/09. Epidemiologia del tabaquismo en Mexico.

50. Levy DT, Zavala-Arciniega LR, Reynales-Shigematsu L, Fleischer N, Li Y, Yuan Z, et al. Measuring Smoking Prevalence in a Middle Income Nation: An Examination of the 100 Cigarettes Lifetime Screen. 2019.

51. U.S. Bureau of the Census. Current Population Survey, September 1993: Tobacco Use Supplement File, Technical Documentation CPS-01. Internet website. Washington, DC: U.S. Bureau of the Census, 2001.

52. Thrasher JF, Villalobos V, Barnoya J, Sansores R, O'Connor R. Consumption of single cigarettes and quitting behavior: a longitudinal analysis of Mexican smokers. BMC Public Health. 2011;11:134. Epub 2011/03/01.

53. Swayampakala K, Thrasher J, Carpenter MJ, Shigematsu LM, Cupertio AP, Berg CJ. Level of cigarette consumption and quit behavior in a population of low-intensity smokers--longitudinal results from the International Tobacco Control (ITC) survey in Mexico. Addict Behav. 2013;38(4):1958-65.

54. McWhorter WP, Boyd GM, Mattson ME. Predictors of quitting smoking: the NHANES I followup experience. J Clin Epidemiol. 1990;43(12):1399-405.

55. Gilpin EA, Pierce JP, Farkas AJ. Duration of smoking abstinence and success in quitting. J Natl Cancer Inst. 1997;89(8):572-6.

56. U.S. DHHS. Reducing the health consequences of smoking: 25 years of progress: a report of the Surgeon General. Atlanta, GA: Centers for Disease Control and Prevention, National Center for Chronic Disease Prevention and Health Promotion, Office on Smoking and Health, 1989 1989. Report No.: DHHS Publication No. [CDC] 89-8411.

57. Hughes JR, Keely J, Naud S. Shape of the relapse curve and long-term abstinence among untreated smokers. Addiction. 2004;99(1):29-38.

58. National Cancer Institute. Cigarette smoking behavior in the United States. In: Burns D, Lee L, Shen L, Gilpin E, Tolley H, Vaughn J, et al., editors. Changes in Cigarette-Related Disease Risks and Their Implication for Prevention and Control, Smoking and Tobacco Control Monograph 8. Bethesda, MD: National Cancer Institute, National Institutes of Health; 1997. p. 13-112.

59. Doll R, Peto R, Boreham J, Sutherland I. Mortality in relation to smoking: 50 years' observations on male British doctors. Bmj. 2004;328(7455):1519.

60. Doll R, Peto R, Wheatley K, Gray R, Sutherland I. Mortality in relation to smoking: 40 years' observations on male British doctors. Bmj. 1994;309(6959):901-11.

61. Reynales-Shigematsu LM. [Literature review of health care costs of diseases attributable to tobacco consumption in the Americas]. Salud Publica Mex. 2006;48 Suppl 1:S190-200. Epub 2007/08/09. Costos de atencion medica de las enfermedades atribuibles al consumo de tabaco en America: revision de la literatura.

62. Reynales-Shigematsu LM, Campuzano-Rincon JC, Sesma-Vasquez S, Juarez-Marquez SA, Valdes-Salgado R, Lazcano-Ponce E, et al. Costs of medical care for acute myocardial infarction attributable to tobacco consumption. Archives of medical research. 2006;37(7):871-9. Epub 2006/09/15.

63. Reynales-Shigematsu LM, Rodriguez-Bolanos Rde L, Jimenez JA, Juarez-Marquez SA, Castro-Rios A, Hernandez-Avila M. [Health care costs attributable to tobacco consumption on a national level in the Mexican Social Security Institute]. Salud Publica Mex. 2006;48 Suppl 1:S48-64. Epub 2007/08/09. Costos de la atencion medica atribuibles al consumo de tabaco en el Instituto Mexicano del Seguro Social.

64. Burns D, Garfinkel L, Samet J, editors. Changes in Cigarette-Related Disease Risks and Their Implication for Prevention and Control. Bethesda, MD: National Institutes of Health, National Cancer Institute; 1997.

65. Inoue-Choi M, McNeel TS, Hartge P, Caporaso NE, Graubard BI, Freedman ND. Non-Daily Cigarette Smokers: Mortality Risks in the U.S. Am J Prev Med. 2019;56(1):27-37.

66. Schane RE, Glantz SA, Ling PM. Nondaily and social smoking: an increasingly prevalent pattern. Arch Intern Med. 2009;169(19):1742-4.

67. Levy DT, Gitchell JG, Chaloupka F. The Effects of Tobacco Control Policies on Smoking Rates: A Tobacco Control Scorecard. J Public Health Manag Pract. 2004;10:338-51.

68. World Health Organization. WHO Report on the Global Tobacco Epidemic, 2009: Implementing smoke-free environments. Geneva: 2009.

69. World Health Organization. WHO report on the global tobacco epidemic, 2011: warning about the dangers of tobacco. Geneva: World Health Organization; 2011. Available from: <http://www.who.int/tobacco/global_report/2011/en/index.html>.

70. World Health Organization. WHO report on the global tobacco epidemic, 2013: Enforcing bans on tobacco advertising, promotion and sponsorship. Geneva: World Health Organization; 2013.

71. Organización Panamericana de la Salud. Informe sobre Control de Tabaco para la Región de las Américas. In: OPS, editor. Washington, DC2013.

72. Levy DT, Cummings KM, Hyland A. Increasing taxes as a strategy to reduce cigarette use and deaths: results of a simulation model. Prev Med. 2000;31(3):279-86.

73. Chaloupka F, Hu T, Warner KE, Yurekli A. The taxation of tobacco products. In: Jha P, Chaloupka F, editors. Tobacco control in developing countries. New York: Oxford University Press; 2000. p. 237-72.

74. Saenz de Miera Juarez B, Guerrero Lopez C, Zuniga Ramiro J, Ruiz Velasco Acosta s. Impuesos al tabaco y politicas para el control tobaco: Resultados Para Mexico. Cuauhtemoc: Fundacio InterAmericana del Corazon Mexico; 2013.

75. Insitituto Nacional de Estadistica y Geografia. Indice Nacional de Precios al Consumidor (INPC). 2019; Available from: <https://www.inegi.org.mx/programas/inpc/2018/default.html#Metadatos>.

76. Fichtenberg CM, Glantz SA. Effect of smoke-free workplaces on smoking behaviour: systematic review. British Medical Journal. 2002;325(7357):188.

77. WHO. Report on the Global Tobacco Epidemic, The MPOWER package. Geneva,: World Health Organization, 2008.

78. Zavala-Arciniega L, Gutiérrez-Torres D, Paz-Ballesteros W, Reynales-Shigematsu L, Fleischer N. Leyes de Ambientes Libres de Humo de Tabaco y Factores asociados a la Exposición al Humo de Tabaco. México 2016. Salud Publica de México. 2018;under review.

79. Thrasher JF, Huang L, Perez-Hernandez R, Niederdeppe J, Arillo-Santillan E, Alday J. Evaluation of a social marketing campaign to support Mexico City's comprehensive smoke-free law. Am J Public Health. 2011;101(2):328-35. Epub 2010/12/18.

80. Thrasher JF, Murukutla N, Perez-Hernandez R, Alday J, Arillo-Santillan E, Cedillo C, et al. Linking mass media campaigns to pictorial warning labels on cigarette packages: a cross-sectional study to evaluate effects among Mexican smokers. Tob Control. 2013;22(e1):e57-65. Epub 2012/07/04.

81. Congreso-de-la-Unión-LIX-Legislatura. Decreto por el que se reforman y adicionan diversas disposiciones de la Ley General de Salud en relación a la publicidad del tabaco Diario Oficial de la Federación.19/01/2004 ed. México, DF. Congreso General2004 [cited March 15 2019]; Available from: dof.gob.mx/nota_detalle.php?codigo=676735&fecha=19/01/2004.

82. Ramirez-Barba EJ, Saro-Boardman E, Vazquez-Guerrero A, Vazquez-Guerrero MA. [The General Law on Smoking Control in Mexico]. Salud Publica Mex. 2008;50 Suppl 3:S372-83. Ley General para el Control del Tabaco en Mexico.

83. Saffer H, Chaloupka F. The effect of tobacco advertising bans on tobacco consumption. J Health Econ. 2000;19(6):1117-37.

84. Blecher E. The impact of tobacco advertising bans on consumption in developing countries. J Health Econ. 2008;27(4):930-42.

85. Perez-Hernandez R, Thrasher JF, Rodriguez-Bolanos R, Barrientos-Gutierrez I, Ibanez-Hernandez NA. [Tobacco advertising and promotions: changes in reported exposure in a cohort of Mexican smokers]. Salud Publica Mex. 2012;54(3):204-12. Epub 2012/06/13. Autorreporte de exposicion a publicidad y promocion de tabaco en una cohorte de fumadores mexicanos.

86. Instituto Nacional de Salud Pública. Global Adult Tobacco Survey (GATS) Mexico. [Adobe file] Mexico City2009 [updated March 31, 2010; cited 2010 June 18]; Available from: <http://www.cdc.gov/tobacco/global/gats/countries/amr/fact_sheets/brazil/>.

87. Levy DT, Mays D, Yuan Z, Hammond D, Thrasher JF. Public health benefits from pictorial health warnings on US cigarette packs: a SimSmoke simulation. Tob Control. 2017;26(6):649-55.

88. Thrasher JF, Hammond D, Fong GT, Arillo-Santillan E. Smokers' reactions to cigarette package warnings with graphic imagery and with only text: a comparison between Mexico and Canada. Salud Publica Mex. 2007;49 Suppl 2:S233-40.

89. Thrasher JF, Perez-Hernandez R, Arillo-Santillan E, Barrientos-Gutierrez I. [Impact of cigarette package health warnings with pictures in Mexico: results from a survey of smokers in Guadalajara]. Salud Publica Mex. 2012;54(3):254-63. Epub 2012/06/13. Impacto de las advertencias con pictogramas en las cajetillas de cigarrillos en Mexico: resultados de una encuesta en fumadores de Guadalajara.

90. Thrasher JF, Villalobos V, Szklo A, Fong GT, Perez C, Sebrie E, et al. Assessing the impact of cigarette package health warning labels: a cross-country comparison in Brazil, Uruguay and Mexico. Salud Publica Mex. 2010;52 Suppl 2:S206-15. Epub 2011/02/03.

91. Abrams D, Graham A, Levy D, Mabry P, Orleans C. Boosting Population Quits through Evidence-Based Cessation Treatment and Policy. Am J Prev Med. 2010;38(38):S351-63.

92. Levy D, Graham A, Mabry P, Abrams D, CT O. Modeling the Impact of Smoking Cessation Treatment Policies on Quit Rates. Am J Prev Med. 2010;38(38):S364-72.

93. Brasil Ministério da Saúde. Brasil. Ministério da Saúde. Coordenação de prevenção e vigilância do câncer. Instituto Nacional de Câncer. Relatório preliminar da implantação do tratamento do fumante no Sistema Único de Saúde - SUS. Rio de Janeiro: Brasil Ministério da Saúde, Coordenação de prevenção e vigilância do câncer, 2007.

94. Bauld L, Bell K, McCullough L, Richardson L, Greaves L. The effectiveness of NHS smoking cessation services: a systematic review. J Public Health (Oxf). 2010;32(1):71-82. Epub 2009/07/30.

95. Bauld L, Boyd KA, Briggs AH, Chesterman J, Ferguson J, Judge K, et al. One-year outcomes and a cost-effectiveness analysis for smokers accessing group-based and pharmacy-led cessation services. Nicotine Tob Res. 2011;13(2):135-45. Epub 2011/01/05.

96. Bauld L, Chesterman J, Ferguson J, Judge K. A comparison of the effectiveness of group-based and pharmacy-led smoking cessation treatment in Glasgow. Addiction. 2009;104(2):308-16. Epub 2009/01/20.

97. Bauld L, Chesterman J, Judge K, Pound E, Coleman T, English Evaluation of Smoking Cessation S. Impact of UK National Health Service smoking cessation services: variations in outcomes in England. Tob Control. 2003;12(3):296-301. Epub 2003/09/06.

98. Tappin DM, Bauld L, Tannahill C, de Caestecker L, Radley A, McConnachie A, et al. The cessation in pregnancy incentives trial (CPIT): study protocol for a randomized controlled trial. Trials. 2012;13:113. Epub 2012/07/24.

99. SectretarÍa d Salud. Programa ds AccIon Espifico 2007-2012 Prevención y Tratamiento de las Adicciones. 2014 [cited 2019 March 20]; Available from: <https://www.paho.org/mex/index.php?option=com_docman&view=download&alias=373-programa-de-accion-especifico-2007-2012-prevencion-y-tratamiento-de-las-adicciones&category_slug=promocion-de-la-salud-y-reduccion-de-riesgos&Itemid=493>.

100. Borland R, Li L, Driezen P, Wilson N, Hammond D, Thompson ME, et al. Cessation assistance reported by smokers in 15 countries participating in the International Tobacco Control (ITC) policy evaluation surveys. Addiction. 2012;107(1):197-205. Epub 2011/09/03.

101. Centers for Disease C, Prevention. Health-care provider screening for tobacco smoking and advice to quit - 17 countries, 2008-2011. MMWR Morb Mortal Wkly Rep. 2013;62(46):920-7. Epub 2013/11/22.

102. Centers for Disease Control. Mexico Global Youth Tobacco Survey. 2012 [cited January 10, 2018]; Available from: <https://www.paho.org/hq/index.php?option=com_docman&view=download&category_slug=2011-9343&alias=41147-mexico-2011-gyts-147&Itemid=270&lang=en>.

103. Hall MG, Fleischer NL, Reynales-Shigematsu LM, Arillo-Santillan E, Thrasher JF. Increasing availability and consumption of single cigarettes: trends and implications for smoking cessation from the ITC Mexico Survey. Tob Control. 2014.

104. Abad-Vivero EN, Thrasher JF, Arillo-Santillan E, Perez-Hernandez R, Barrientos-Gutierrez I, Kollath-Cattano C, et al. Recall, appeal and willingness to try cigarettes with flavour capsules: assessing the impact of a tobacco product innovation among early adolescents. Tob Control. 2016;25(e2):e113-e9.

105. Thrasher JF, Abad-Vivero EN, Moodie C, O'Connor RJ, Hammond D, Cummings KM, et al. Cigarette brands with flavour capsules in the filter: trends in use and brand perceptions among smokers in the USA, Mexico and Australia, 2012-2014. Tob Control. 2016;25(3):275-83.

106. Barrientos-Gutierrez I, Lozano P, Arillo-Santillan E, Morello P, Mejia R, Thrasher JF. "Technophilia": A new risk factor for electronic cigarette use among early adolescents? Addict Behav. 2019;91:193-200.

107. Gravely S, Driezen P, Ouimet J, Quah ACK, Cummings KM, Thompson ME, et al. Prevalence of awareness, ever-use and current use of nicotine vaping products (NVPs) among adult current smokers and ex-smokers in 14 countries with differing regulations on sales and marketing of NVPs: cross-sectional findings from the ITC Project. Addiction. 2019.

108. Lozano P, Arillo-Santillan E, Barrientos-Gutierrez I, Reynales Shigematsu LM, Thrasher JF. E-Cigarette Social Norms and Risk Perceptions Among Susceptible Adolescents in a Country That Bans E-Cigarettes. Health Educ Behav. 2019;46(2):275-85.

109. Lozano P, Barrientos-Gutierrez I, Arillo-Santillan E, Morello P, Mejia R, Sargent JD, et al. A longitudinal study of electronic cigarette use and onset of conventional cigarette smoking and marijuana use among Mexican adolescents. Drug Alcohol Depend. 2017;180:427-30.

110. Thrasher JF, Abad-Vivero EN, Barrientos-Gutierrez I, Perez-Hernandez R, Reynales-Shigematsu LM, Mejia R, et al. Prevalence and Correlates of E-Cigarette Perceptions and Trial Among Early Adolescents in Mexico. J Adolesc Health. 2016;58(3):358-65.

111. Zavala-Arciniega L, Reynales-Shigematsu LM, Lozano P, Rodriguez-Andrade MA, Arillo-Santillan E, Thrasher JF. Patterns of awareness and use of electronic cigarettes in Mexico, a middle-income country that bans them: Results from a 2016 national survey. Prev Med. 2018;116:211-8.

112. Ryan H, Trosclair A, Gfroerer J. Adult current smoking: differences in definitions and prevalence estimates--NHIS and NSDUH, 2008. J Environ Public Health. 2012;2012:918368.

113. Hughes JR. Reduced smoking: an introduction and review of the evidence. Addiction. 2000;95(Suppl 1):S3-7.

114. Hymowitz N, Cummings KM, Hyland A, Lynn WR, Pechacek TF, Hartwell TD. Predictors of smoking cessation in a cohort of adult smokers followed for five years. Tobacco Control. 1997;6 Suppl 2:S57-S62.

115. Hymowitz N, Sexton M, Ockene J, Grandits G. Baseline factors associated with smoking cessation and relapse. MRFIT Research Group. Prev Med. 1991;20(5):590-601.

116. Sung H, Hu T, Keeler T. Cigarette taxation and demand: An empirical model. Contemporary Economic Policy. 1994;12:91-100.

117. Chaloupka FJ, Hu T, Warner KE, Jacobs R, Yurekli A. The taxation of tobacco products. In: Jha P, Chaloupka F, editors. Tobacco control in developing countries: Oxford University Press; 2000. p. 237-72.

118. Sarntisart I. An Economic Analysis of Tobacco Control in Thailand. Washington, D.C.: World Bank, 2003 Economics of Tobacco Control Paper No. 15.
